# Intrinsic Photo‐Crosslinkable Semiconductive Small‐Molecule Crystals (i‐PSSCs) for Patterning Electronic Devices

**DOI:** 10.1002/advs.202504711

**Published:** 2025-08-30

**Authors:** Huaqing Li, Xiaoguang Hu, Lei Zhang, Qingqing Sun, Chuan Liu, Linlin Zhang, Takeo Minari, Xuying Liu

**Affiliations:** ^1^ School of Materials Science and Engineering Zhengzhou University Zhengzhou 450001 China; ^2^ Beijing Advanced Innovation Center for Soft Matter Science and Engineering Beijing University of Chemical Technology Beijing 100029 China; ^3^ State Key Laboratory of Optoelectronic Materials and Technologies School of Electronics and Information Technology Sun Yat‐sen University Guangzhou 510275 China; ^4^ Printed Electronics Group, Research Center for Functional Materials National Institute for Materials Science (NIMS) Tsukuba Ibaraki 305‐0044 Japan

**Keywords:** intrinsic crosslinking, organic phototransistors, photopatterning, semiconductive small‐molecule crystals

## Abstract

Precise patterning of small‐molecule semiconductive crystals without external chemical additives remains a significant challenge. Herein, intrinsic photo‐crosslinkable semiconductive small‐molecule crystals (i‐PSSCs) are designed and synthesized by associating [1]benzothieno[3,2‐b]benzothiophene core with diacetylene‐ended groups. The i‐PSSCs undergo self‐crosslinking directly upon UV light irradiation to yield micron‐scale patterned crystalline films through a combination of photo‐crosslinking and solvent rinsing. The molecular packing remains intact before and after patterning. Therefore, the electrical performance of the organic thin‐film transistors fabricated from both pristine and patterned i‐PSSCs films shows minimal difference, with maximum field‐effect mobilities of 0.46 and 0.25 cm^2^ V^−1^ s^−1^, respectively. Moreover, the i‐PSSCs in a transistor array exhibit high sensitivity and selective response to UV patterns, enabling bio‐inspired vision systems that mimic human retinal extraction of image descriptors. This work offers a valuable strategy for developing i‐PSSCs for UV‐selective artificial vision applications.

## Graphical abstract

A novel intrinsic photo‐crosslinkable semiconductive small molecule is designed and synthesized. The solution‐processed crystalline film can be patterned by in situ photopolymerization under UV light irradiation without external crosslinking agents, and the crystalline order and charge transport properties are preserved, demonstrating potential for high‐precision organic electronic devices.

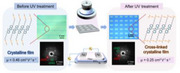

## 1 Introduction

Organic semiconductors have garnered significant attention for their potential in flexible electronics,^[^ ^1^ ^]^ organic optoelectronics,^[^ ^2^, ^3^ ^]^ and sensor technologies^[^ ^4^ ^]^ due to their mechanical flexibility, cost‐effectiveness, and tunable chemical structures. These materials play a crucial role in the active layers of electronic devices, where precise patterning with controlled position and thickness is essential for minimizing crosstalk and leakage currents, enhancing device integration, and improving performance consistency.^[^ ^5^ ^]^ As such, the development of effective patterning technologies has become a critical aspect of fabricating high‐performance organic semiconductor devices.

Traditional patterning methods, such as photolithography, are ill‐suited for organic semiconductors due to the harsh conditions required, including high temperatures, photoresist residues, and chemical etching treatments.^[^ ^6^ ^]^ Solution‐based processes,^[^ ^7^ ^]^ on the other hand, offer several advantages, including low‐temperature fabrication and scalability, which are crucial for producing large‐area flexible devices.^[^ ^8^ ^]^ Techniques like inkjet printing,^[^ ^9^ ^]^ microcontact printing,^[^ ^10^ ^]^ and selective surface energy engineering^[^ ^11^, ^12^ ^]^ have been explored, enabling the creation of high‐resolution patterns.^[^ ^13^ ^]^ However, a significant challenge persists: organic semiconductors are inherently vulnerable to solvent‐induced degradation, particularly during subsequent solution‐based deposition steps. This instability can lead to partial dissolution of patterned films, compromising device performance, reproducibility, and reliability. One promising strategy to address this issue is the photo‐crosslinking of organic small molecules and polymers, which enhances their resistance to solvents.

In situ photo‐crosslinking not only improves the solvent resistance of organic semiconductor films but also enables patterning through solvent rinsing, achieving both improved stability and precision. Several photo‐crosslinkers, such as azide,^[^ ^14^ ^]^ diazirine,^[^ ^15^ ^]^ thiol‐ene,^[^ ^16^ ^]^ and diacetylene,^[^ ^17^ ^]^ have been incorporated into organic semiconductors to enable photolithographic patterning. Recent studies have demonstrated that blending polymer semiconductors with these photo‐crosslinkers allows for the formation of patterned films with channel lengths as small as 2 µm.^[^ ^18^ ^]^ Azide‐ and diazirine‐based photo‐crosslinkers, in particular, can crosslink both semiconducting and dielectric polymers, as well as nanoparticles, through light‐induced carbene insertions into molecular chains.^[^ ^19^ ^]^ However, multi‐component photoresists for polymeric semiconductors can suffer from phase separation, which compromises material stability and device reproducibility in large‐area arrays. To address these challenges, photo‐crosslinkable single‐component semiconductors have been developed by integrating photoactive groups such as aliphatic azides^[^ ^20^ ^]^ and cinnamates^[^ ^21^ ^]^ into the side chains of conjugated polymers. These materials undergo crosslinking under UV or electron‐beam irradiation, enabling the formation of insoluble submicron or micron‐scale patterns. In contrast to polymer‐based systems,^[^ ^14^, ^22^ ^]^ small‐molecule semiconductors provide superior molecular uniformity and crystalline order, which not only improve film quality and reproducibility but also enable higher charge carrier mobility.^[^ ^23^ ^]^ Crucially, preserving the molecular packing during photo‐crosslinking is essential for maintaining the high performance of the patterned films.

In this work, we introduce a class of intrinsic photo‐crosslinkable semiconductive small‐molecule crystals (i‐PSSCs). The π‐conjugated core [1]benzothieno[3,2‐b]benzothiophene (BTBT) is used to ensure high crystallinity and mobility, while diacetylene (DA) photo‐crosslinkable groups are incorporated to facilitate crosslinking under UV light. Blade‐coated crystalline thin films of molecule **6** were successfully photo‐patterned upon exposure to UV light (254 nm, 1 mW cm^−^^2^) and subsequent developing treatment, and their tight intermolecular herringbone packing was retained. Notably, organic thin‐film transistors (OTFTs) fabricated from molecule **6** films exhibited similar performance before and after photopatterning, with maximum charge mobilities of 0.46 and 0.25 cm^2^ V^−1^ s^−1^, respectively. Furthermore, the OTFT array exhibited high sensitivity and selectivity to UV (365 nm) patterns, effectively extracting image descriptors from “H”‐shaped characters, demonstrating its potential for UV‐selective bio‐inspired vision systems, such as artificial retinal imaging.

## 2 Results and Discussion

### 2.1 Synthesis of 6, 7, and 8

**Scheme 1:**
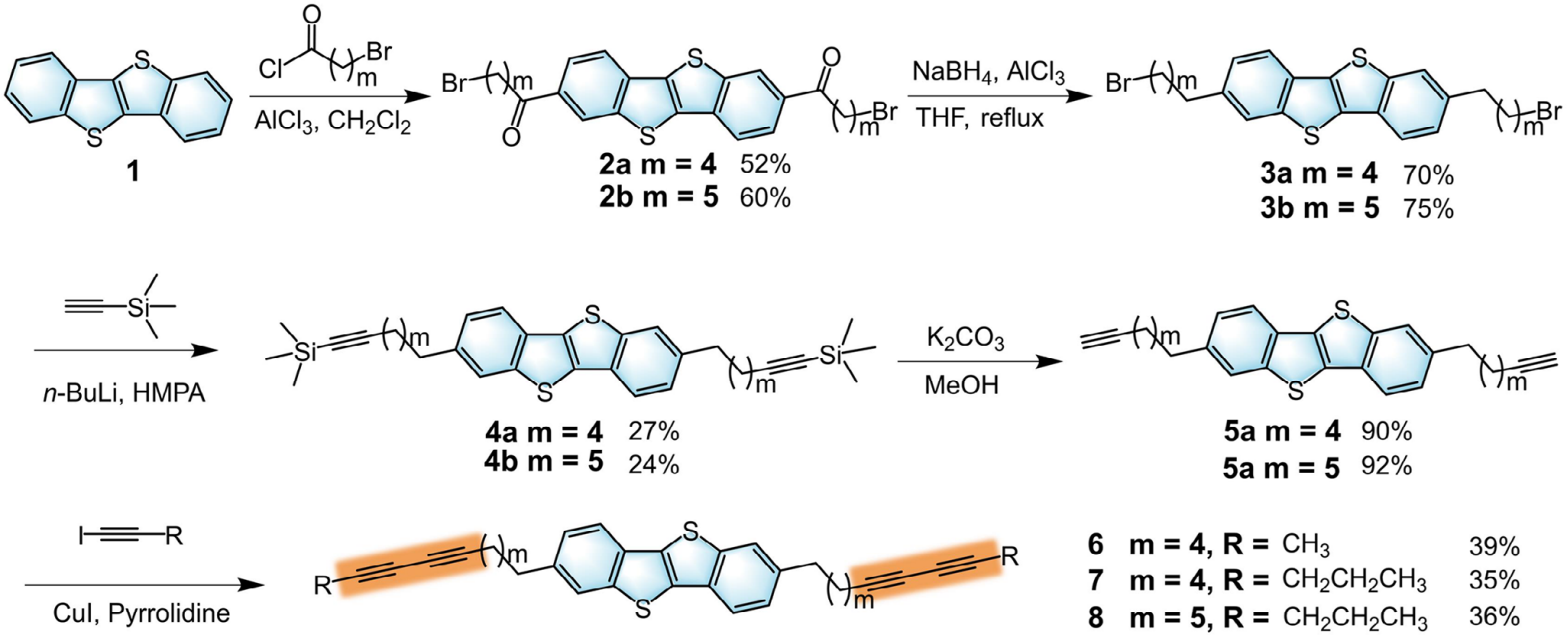
Synthetic routes of compounds 6, 7, and 8.

The target compounds were synthesized from BTBT (**1**) via a five‐step process as shown in **Scheme** **1**. First, a Friedel‐Crafts acylation reaction was conducted to introduce two bromoacyl chains at positions 2 and 7 of the BTBT core. The keto group in compound **2** was then reduced using a sodium borohydride/anhydrous aluminum chloride (III) system, yielding compound **3**.^[^ ^24^ ^]^ Subsequently, the alkyne precursors **5** were synthesized in a two‐step sequence^[^ ^25^ ^]^ beginning with the alkylation of compound **3** with trimethylsilylacetylene, followed by desilylation with potassium carbonate in methanol. Finally, compounds **6**, **7,** and **8** containing DA units were obtained through Sonogashira coupling reaction of compound 5 with iodoalkyne.^[^ ^26^ ^]^ The synthetic details are provided in the Supporting Information (Figures S1–S33, Supporting Information). Thermal gravimetric analysis revealed high decomposition temperatures of 453, 446, and 432 °C for **6**, **7,** and **8**, respectively (**Table** **1**), indicating their excellent thermal stability (Figure S36, Supporting Information).

**Table 1:** Summary of the optical and electrochemical properties of **6**, **7**, and **8**. Charge mobilities, threshold voltages, and on‐off current ratios of OTFTs with the pristine thin film of **6** and **8**, and the corresponding patterned thin film of **6**.

Compound	*E*_HOMO_ ^a)^ [eV]	*E*_g_^opt^ ^b)^ [eV]	*E*_LUMO_ ^c)^ [eV]	UV irradiation	*µ*_h, adv_ [*µ*_h, max_]^d)^ [cm^2 ^v^−1^ s^−1^]	*V*_th_ [V]	*I*_on_/*I*_off_
**6**	−5.69	3.63	−2.06	Before	0.29 ± 0.081 (0.46)	−30.68 ± 2.611	10^6^–10^7^
				After	0.10 ± 0.048 (0.25)	−44.7 ± 2.352	10^5^–10^6^
**7**	−5.69	3.63	−2.06	–	–	–	–
**8**	−5.68	3.63	−2.05	Before	0.01 ± 0.004 (0.015)	−25.33 ± 2.132	10^4^–10^5^

^a)^ HOMO energy calculated by *E*_HOMO_ =−(*E* ^ox^_sample_−*E* ^ox^_Fc/Fc+_ + 4.8);

^b)^ Band‐gap energy obtained from the edge of the thin film absorption spectra;

^c)^ LUMO energy calculated by subtracting the optical bandgap from HOMO;

^d)^ All average mobilities are based on 36 devices, and maximum values are shown in parentheses.

### 2.2 Crystal Packing

**Figure 1:**
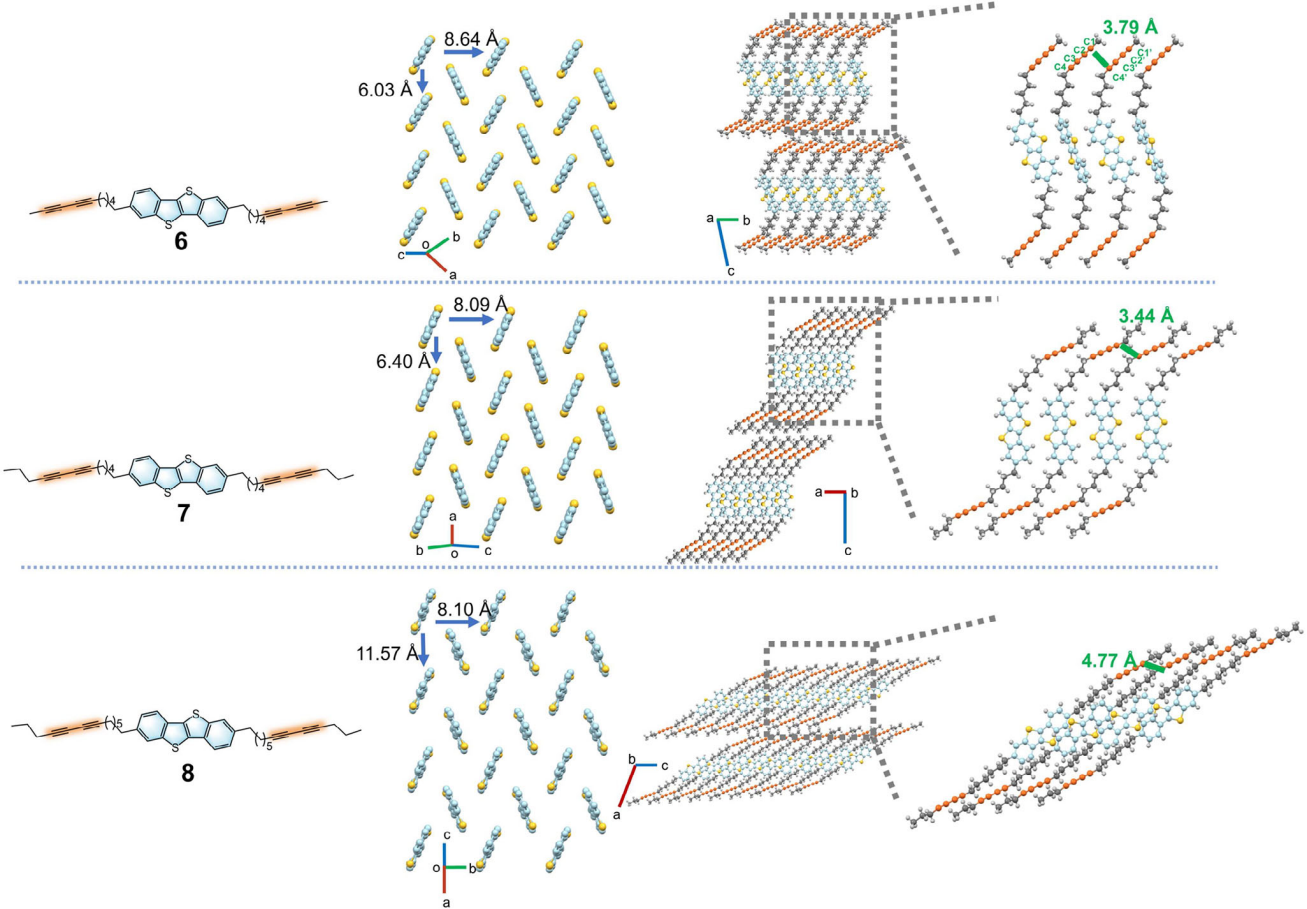
Single‐crystal X‐ray structures and partial solid‐state packing of 6, 7, and 8 with a typical herringbone packing. In the herringbone packing structures, the alkyl chain details are omitted for simplification.

Single crystals of **6**, **7**, and **8** were grown by slow solvent evaporation from THF/Hexane or toluene solutions, and their structures are depicted in **Figure** **1**. Compounds **6** and **7** crystallize in the triclinic P‐1 space group, while compound **8** adopts a monoclinic system with the space group P21/c. All three molecules exhibit layered crystalline packing with each layer adopting a typical herringbone packing. The intermolecular distances between adjacent molecules are measured at 6.03 and 8.64 Å for **6**, and 6.40 and 8.09 Å for **7**. These close intermolecular distances facilitate strong C─H∙∙∙π (2.869–2.872 Å) and S‐π (3.359–3.493 Å) interactions between the neighboring molecules in both compounds (Figure S35, Supporting Information), which is expected to enable efficient charge transfer in these molecules.^[^ ^27^ ^]^ In contrast, longer intermolecular distances (11.57 and 8.10 Å) are observed in compound **8**, which are too wide to support efficient charge transfer. Additionally, the adjacent C≡C─C≡C groups in compounds **6** and **7** overlap with a distance of 3.79 and 3.44 Å between C1 and C4′, which is necessary and favorable for 1,4‐coupling reaction.^[^ ^28^ ^]^ Notably, the distance between C1 and C4′ in compound **8** is particularly long (4.77 Å), making the reaction of adjacent DA segments highly unlikely.

### 2.3 Optical and Electrochemical Properties

The optical property was investigated using UV–vis absorption spectroscopy. Strong absorption bands in the UV region (230–340 nm) were observed for compounds **6**, **7**, and **8** in dichloromethane (DCM, 2 × 10^−^⁵ m), with prominent peaks located at 313, 270, and 241 nm for the three molecules (Figure S38, Supporting Information). The peaks are attributed to the *π*→*π* ^*^ transition of the BTBT conjugated core.^[^ ^29^, ^30^ ^]^ Compared to the solution sample, thin films of **6** and **8** exhibit significant redshifts in absorption, with intense peaks at 252 and 345 nm for film **6**, and 220, 273, and 326 nm for film **8** (Figure S39, Supporting Information). This redshift is strongly associated with the *π*–*π* packing interaction between molecules in the aggregated state. Cyclic voltammetry (CV) measurements reveal the redox behaviors of these compounds. The oxidation potentials of **6**, **7**, and **8** were measured at E1/2 OX = 1.24, 1.24, and 1.23 V, with reduction waves observed at E1/2 red = −0.81, −0.81, and −0.78 V (vs Fc^+^/Fc), respectively (Figure S41, Supporting Information). The highest occupied molecular orbital (HOMO) energy levels of **6**, **7**, and **8** were calculated from CV to be −5.69, −5.69, and −5.68 eV, respectively. Correspondingly, the lowest unoccupied molecular orbital (LUMO) energy levels were derived by subtracting the optical bandgap from HOMO, yielding −2.06, −2.06, and −2.05 eV (Table 1).

### 2.4 Intrinsic Photo‐Crosslinkable Semiconductive Small‐Molecule Crystals (i‐PSSCs)

**Figure 2:**
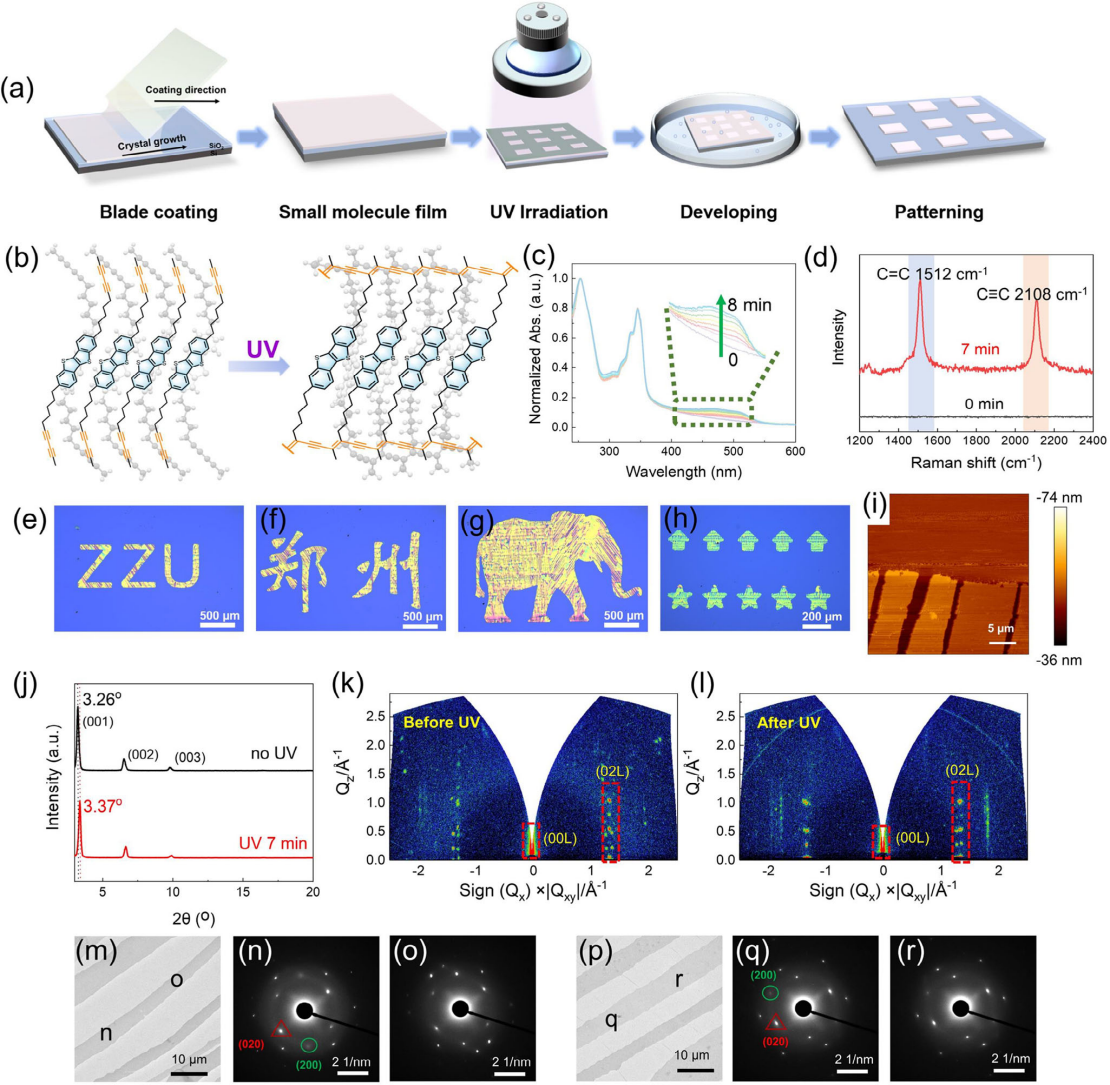
a) The photo‐patterning process of the i‐PSSCs 6. b) Topochemical photochemical reaction of monomer 6. c) UV–vis spectra for illumination times ranging from 0 to 8 min. d) Raman spectra of 6 for 0 and 7 min of illumination. e–h) Optical microscope images of patterned film 6. i) AFM image about the edges of the pattern in Figure S59a (Supporting Information). j) XRD patterns of 6 before and after illumination. k,l) GIWAXS images of 6 with incident X‐ray beams perpendicular to the coating direction of the films. m–o) TEM images of 6 before illumination and its corresponding SAED patterns recorded from the different positions marked in (m). p–r) TEM images of 6 after illumination and its corresponding SAED patterns recorded from the different positions marked in (p).

The processes for preparing i‐PSSCs were illustrated in **Figure** **2**a. Initially, a large‐area crystalline semiconductor thin film was grown on a polystyrene (PS)‐modified^[^ ^31^ ^]^ SiO_2_/Si substrate using the solution shearing method. The film thickness was controlled in the range of 36–300 nm by adjusting the coating speed (Figures S48 and S49, Supporting Information). Due to the anisotropic crystallization behavior during blade coating, the films exhibited well‐aligned ribbon‐like crystalline domains oriented along the coating direction, as observed in atomic force microscopy (AFM), transmission electron microscopy (TEM), and polarizing optical microscope (POM) (Figures S46–S49 and S51, Supporting Information). The patterned thin films of i‐PSSCs were then obtained though successive UV light irradiation with a photomask and solvent rinsing. Notably, compound **7** did not form continuous films on the substrate, likely due to its extended side chains (Figure S34, Supporting Information), which weaken intermolecular interactions and disrupt the ordered molecular arrangement.^[^ ^32^ ^]^

Additionally, long side chains are likely to impact the wettability and molecular spreading ability on the substrate.^[^ ^33^ ^]^ The schematic diagram of ideal topological polymerization (e.g., **6**) is depicted in Figure 2b, where the adjacent DAs undergo topochemical 1,4‐polymerization under UV irradiation, forming poly(ene‐yne) chains that crosslink the molecules while maintaining the ordered packing of the BTBT cores.^[^ ^34^, ^35^ ^]^ UV–vis absorption measurement of the i‐PSSCs films was performed during illumination (Figure 2c). A new absorption in the range of 400–550 nm appeared and gradually increased during the illumination of 254 nm light (1 mW cm^−^^2^), indicating the crosslinking of DA units within the i‐PSSCs film.^[^ ^36^ ^]^ Correspondingly, Raman spectra showed significant stretching bands at 1512 and 2108 cm^−1^ after 7 min of UV irradiation(Figure 2d), attributed to the double and triple bonds in the resulting C═C─C≡C moieties,^[^ ^37^ ^]^ supporting the formation of crosslinked poly(ene‐yne) chains. When the film was covered with a photomask and irradiated with UV light, various patterns—including letters, Chinese characters, animals, and geometric shapes—were successfully generated by immersing the film in acetonitrile for 2 min to remove the unexposed areas (Figure 2e–h). As shown in Figure 2i and Figure S44a,b (Supporting Information), the patterned edges are clearly defined in the AFM height maps, indicating that the i‐PSSC films of compound **6** were effectively crosslinked to resist solvent rinsing. To further evaluate the patterning fidelity, the line‐edge roughness (LER) was analyzed based on AFM profiles across the pattern boundaries, yielding an average LER of ≈65 nm. This result confirms the potential of our system for high patterning precision, which is suitable for phototransistor array fabrication and compares favorably with other organic photopatterning methods (Table S3, Supporting Information). In contrast, no significant change in UV absorption was observed for thin film **8** (Figure S56, Supporting Information) under 254 nm illumination for 20 min. Additionally, no stretching bands appeared in the Raman spectra, confirming that compound **8** did not undergo topological polymerization, consistent with its molecular packing (Figure 1).

To further investigate the film quality of i‐PSSCs after photo‐crosslinking and assess the effect of molecular cross‐linking on the molecule packing, X‐ray diffraction (XRD), POM, and AFM characterizations were carried out. The XRD patterns of films of molecule **6**, before and after UV illumination, both exhibit three diffraction peaks, which correspond to the (00L) plane family. This indicates that the ab plane of the crystal **6** is aligned parallel to the substrate (Figure 2j). After 7 min UV irradiation, the peak corresponding to (00L) shifted to a higher angle (2θ) from 3.26° to 3.37°, accompanied with a decrease in the interlayer stacking distance from 27.1 to 26.1 Å. The reduction in interlayer spacing suggests the occurrence of a polymerization reaction between the molecules.^[^ ^38^ ^]^ Furthermore, no significant change was observed in the regular microstripe crystalline morphology of the film after polymerization under POM (Figure S52, Supporting Information). Additionally, after polymerization, the surface root‐mean‐square (RMS) roughness slightly increased from 0.59 to 0.67 nm, while the step height decreased from 2.66 to 2.54 nm (Figures S48 and S54, Supporting Information), which is consistent with the packing distance observed in the XRD results.

Grazing‐incidence wide‐angle X‐ray scattering (GIWAXS) was also employed to gain insights into the molecular packing in the films of the i‐PSSCs **6** before and after irradiation. The sharp and discrete Bragg diffraction patterns (Figure 2k,l) observed in both out‐of‐plane and in‐plane directions further confirm the high crystallinity and long‐range ordered packing of the i‐PSSCs films, both before and after cross‐linking. When the incident X‐ray is perpendicular to the coating direction, diffraction spots corresponding to the (02L) plane, related to the b‐axis, are observed along the plane direction, revealing their ordered arrangement in the b‐axis direction. Importantly, the d‐spacing of the (001) peak in the out‐of‐plane direction shifted from 26.3 Å (*q_z_* = 0.239 Å^−1^) before illumination to 25.6 Å (*q_z_* = 0.245 Å^−1^) after illumination, which is consistent with the vertical height change of the molecules noted earlier. Thus, during the polymerization process of the i‐PSSCs, the crystal order of the monomers remains intact, while the lattice parameters undergo slight changes. TEM images in Figure 2m,p reveal a smooth surface morphology, and the corresponding selected area electron diffraction (SAED) patterns in Figure 2n,o,q,r show consistent diffraction spots across different areas of the film, further confirming the excellent crystal quality and orientation of the film **6** both before and after crosslinking. In summary, it is evident that the b‐axis is the preferred direction of crystal growth, and the molecule packing in the thin film of molecule **6** remains intact after illumination, providing optimal intermolecular interactions for charge transport.

### 2.5 Charge Transport Properties and Application

**Figure 3:**
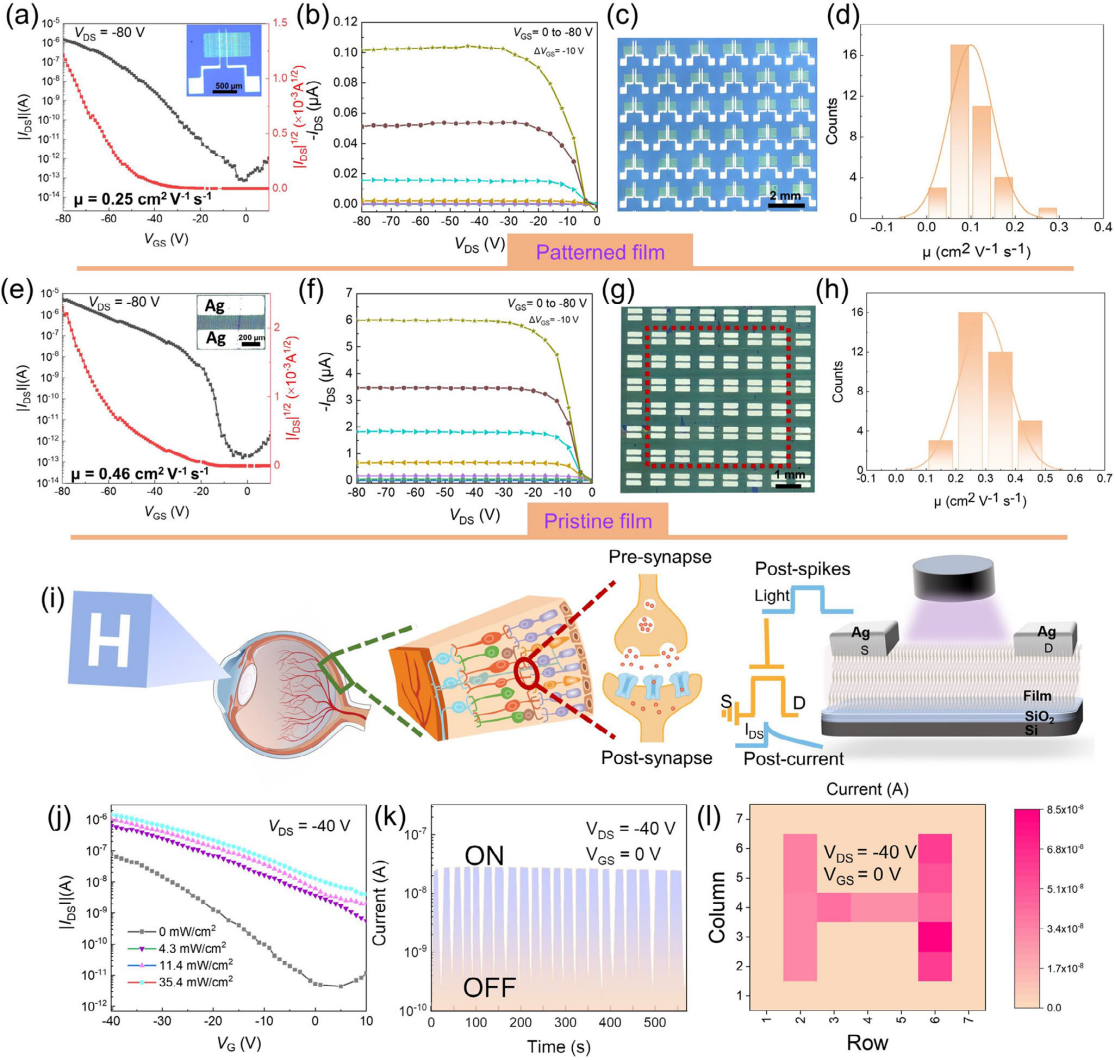
a,b) Typical transfer and output curves, c) the optical image of patterned OTFT array, d) mobility distribution of 36 OTFT devices about the patterned i‐PSSCs 6. e,f) Typical transfer and output curves, g) the optical image of OTFT array, h) mobility distribution of 36 OTFT devices about the pristine film of 6. i) The artificial vision system composed of a detection unit and schematic illustration of organic photodetector device based on patterned film of 6. j) The transfer curves of the OPTs in various illumination intensities. k) Photo‐response measurements of the device in the dark and under illumination. l) Image detection of device array with an input image of letter “H” (under illumination with “H” type mask on top).

To investigate the charge transfer characteristics of the i‐PSSCs, bottom gate and top contact (BGTC) OTFTs (Figure S59, Supporting Information) were fabricated using the corresponding patterned thin films as active layers. Detailed procedures are outlined in the Supporting Information. In brief, a transparent rectangular mask (length: 1000 µm, width: 500 µm) was placed on a crystalline film of molecule **6**, which was scraped onto a PS‐modified SiO_2_/Si substrate and exposed to 254 nm light. After development with acetonitrile, an electrode mask was aligned with the semiconductor position, and thermally evaporated Ag (80 nm) was used as the source and drain electrodes. It is crucial that the crystal growth direction is parallel to the conductive channel. The mobility was measured in air and calculated from the transfer curve in the saturation state. A 6 × 6 rectangular array was selected from the same batch of devices for testing (**Figure** **3**c). After analyzing 36 transfer curves (Figure S66, Supporting Information), the highest mobility of the patterned film was obtained as 0.25 cm^2^ V^−1^ s^−1^ and an on/off current ratio approaching 10⁶. The representative transfer and output *I*–*V* curves of the fabricated OTFT are presented in Figure 3a,b.

For comparison, the same test was carried out on BGTC OTFTs fabricated from the pristine thin film of **6**. Similarly, 36 transfer curves were analyzed (Figure 3g), and the highest mobility of the pristine film was found to be 0.46 cm^2^ V^−1^ s^−1^ and an on/off current ratio approaching 10⁷. The I‐V curves and OTFT image are shown in Figure 3e,f. This suggests that the performance remains well‐preserved after photo‐patterning. Moreover, both pristine and patterned film‐based OTFTs exhibit narrow performance distributions, demonstrating excellent uniformity in device performance (Figure 3d,h). The slight reduction in mobility and other parameters after illumination may be due to the discontinuity of the cross‐linked molecular chain, which generates a large number of defects that affect charge transfer. The maximum mobilities of the OTFTs before and after crosslinking were 0.46 and 0.25 cm^2^ V^−1^ s^−1^, respectively. These values are lower than BTBT derivatives such as C8‐BTBT and Ph‐BTBT.^[^ ^39^, ^40^, ^41^ ^]^ This can be attributed to the influence of diacetylene side chains on molecular packing, increased grain boundaries and misorientation from solution‐based film deposition, and additional defects or disorder introduced during the photo‐crosslinking process. In addition, OTFTs were also fabricated with the film **8** as the active layer, and the typical transfer and output curves are shown in Figure S62 (Supporting Information). Consistent with the single‐crystal analysis (Figure 1), the stacking of **8** hinders charge transfer, resulting in an order‐of‐magnitude lower mobility of 0.015 cm^2^ V^−1^ s^−1^. Therefore, only **6** effectively combine the functions of both a semiconductor and an intrinsic photo‐patternable crystal.

The semiconducting properties of the i‐PSSCs **6**, associated with its photopatterning ability, make it a promising candidate for phototransistor applications. Organic phototransistors (OPTs) integrate both light detection (Figure 3i) and electrical signal amplification functions, with wide‐ranging applications in fields such as biology and communication.^[^ ^42^ ^]^ To assess the feasibility of OTFT devices in simulating visual neurons, source‐drain currents were measured under 365 nm UV light at varying power levels. The typical transmission characteristics of the phototransistor under dark and illuminated conditions are shown in Figure 3j, where the current increases as the light power rises. The current changes result from the accumulation of photogenerated carriers around the source/drain, leading to band bending in the semiconductor and reducing the potential barrier for hole injection into the source electrode.^[^ ^43^, ^44^ ^]^ Compared to the dark condition, more holes participate in the charge transport process at the same driving voltage (Figure S70, Supporting Information), which increases the photocurrent. Light irradiation can independently control the output current of OPTs. The measurement in Figure 3k was performed by periodically switching the UV illumination (4.3 mW cm^−^^2^, V_DS_ = −40 V, V_GS_ = 0 V) on and off, with a total of 21 consistent ON–OFF current cycles observed (ON–OFF ratio ≈10^2^), demonstrating stable photodetector performance. A skeleton mask with the symbol “H” was used to cover the silicon wafer with a 7 × 7 imaging pixel matrix (Figure S72, Supporting Information), and the output current from the light‐exposed areas remained ≈10^−^⁸ A (Figure 3l). By recording the current changes across the matrix, the character shape can be accurately reproduced, highlighting the potential of patterned i‐PSSCs as UV‐selective neuromorphic visual sensors for bio‐inspired electronic systems.

## 3 Conclusion

In conclusion, this work presents a strategy for developing i‐PSSCs by combining a semiconductor BTBT core with photo‐crosslinkable diacetylene groups. The molecule packing is strongly influenced by the alkyl chain and terminal substituents on the DA units, neighboring DAs only overlapping in film **6**, which has a terminal methyl group. As a result, only compound **6** can self‐crosslink under UV light irradiation, and the molecular packing in the highly ordered crystalline film remains intact after photo‐patterning. Importantly, the electrical performance of OTFTs based on the patterned crystalline films is comparable to that of devices made from pristine crystalline films, with maximum mobility values of 0.25 and 0.46 cm^2^ V^−1^ s^−1^. Furthermore, the patterned thin films exhibit high UV responsiveness, opening new avenues for application in UV‐selective, bio‐inspired neuromorphic visual electronics.

## 4 Experimental Section

Experimental details, NMR and HRMS spectra, TGA and DSC spectra, CV and optical absorption spectra, theoretical calculation, POM, AFM, XRD images, and CCDC 2415605 (**6** at 293 K), 2415606 (**7** at 100 K) and 2415608 (**8** at 293 K) contains the crystallographic data can be found in the Supporting Information.

## Conflict of Interest

The authors declare no conflict of interest.

## Supporting information



Supporting Information

Supporting Information

## Data Availability

The data that support the findings of this study are available from the corresponding author upon reasonable request.
