# Supplementary material for: Intrinsic Photo‐Crosslinkable Semiconductive Small‐Molecule Crystals (i‐PSSCs) for Patterning Electronic Devices
Source: Adv Sci (Weinh). 2025 Aug 30;12(44):e04711. doi: 10.1002/advs.202504711 (PMC12667458; doi:10.1002/advs.202504711)
Supplement: Supplementary file 1 — Supporting Information [file ADVS-12-e04711-s001.docx]

Supporting Information

**Intrinsic Photo-crosslinkable Semiconductive Small-Molecule Crystals (i-PSSCs) for Patterning Electronic Devices**

Huaqing Li,^[a]^ Xiaoguang Hu,*^[a]^ Lei Zhang,^[b]^ Qingqing Sun,^[a]^ Chuan Liu,^[c]^ Linlin Zhang,^[a]^ Takeo Minari,^[d]^ Xuying Liu*^[a]^

[a] H. Li, Dr. X. Hu, Dr. Q. Sun, Dr. L. Zhang, Prof. X. Liu,
School of Materials Science and Engineering, Zhengzhou University, Zhengzhou 450001, China
E-mail: xghu@zzu.edu.cn; liuxy@zzu.edu.cn

[b] Prof. L. Zhang
Beijing Advanced Innovation Center for Soft Matter Science and Engineering, Beijing University of Chemical Technology, Beijing 100029, China.

[c] Prof. C. Liu
State Key Laboratory of Optoelectronic Materials and Technologies, School of Electronics and Information Technology, Sun Yat-sen University, Guangzhou 510275, China

[d] Prof. T. Minari
Printed Electronics Group, Research Center for Functional Materials, National Institute for Materials Science (NIMS), Tsukuba, Ibaraki 305-0044, Japan

**Table of contents**

[1. General Methods and Materials 2](#_Toc202946993)

[2. Synthetic details 3](#_Toc202946994)

[Synthesis of 2a 3](#_Toc202946995)

[Synthesis of 2b 3](#_Toc202946996)

[Synthesis of 3a 4](#_Toc202946997)

[Synthesis of 3b 4](#_Toc202946998)

[Synthesis of 4a 4](#_Toc202946999)

[Synthesis of 4b 5](#_Toc202947000)

[Synthesis of 5a 5](#_Toc202947001)

[Synthesis of 5b 6](#_Toc202947002)

[Synthesis of 6 6](#_Toc202947003)

[Synthesis of 7 7](#_Toc202947004)

[Synthesis of 8 7](#_Toc202947005)

[3. NMR and MALDI-TOF-MS spectra 8](#_Toc202947006)

[4. Single crystal X-ray diffraction 22](#_Toc202947007)

[5. TGA and DSC curves 24](#_Toc202947008)

[6. Optical absorption, electrochemical properties and DFT calculations 25](#_Toc202947009)

[7. Preparation and patterning of organic small molecule films 28](#_Toc202947010)

[8. Film quality characterization 30](#_Toc202947011)

[9. Fabrication of the OTFT devices 35](#_Toc202947012)

[10. Supporting references 43](#_Toc202947013)

1. General Methods and Materials

All reagents were purchased from Macklin, Aladdin, TCI and Adamas and used as received. Column chromatography was performed with General-Reagent silica gel (300-400 mesh). All reaction mixtures and column eluents were monitored by TLC using commercial Huanghai glass plates (HSGF 254, 2.5 × 8 cm). The plates were visualized under UV radiation at 254 and 365 nm. UV/vis/NIR absorption spectra were recorded on an Agilent Cary 5000 UV-Vis-NIR spectrophotometer. Fourier transform infrared (FTIR) test on Bruker TENSOR 27. MALDI-TOF mass spectra (MS) were recorded on a SHIMADZU iD plus Performance using anthracene-1, 8, 9-triol as matrix. NMR spectra were obtained on a BRUKER AVANCE HD 400 MHz. Cyclic voltammograms were measured on a Shanghai Chenhua CHI 660E electrochemical workstation. Transmission electron microscopy (TEM) images were measured using a JEM-F200. X-ray diffraction (XRD) images were recorded on Rigaku SmartLab SE, the 2θ angle was scanned from 2^o^ to 30^o^. Two-dimensional grazing-incidence wide-angle X-ray scattering (GIWAXS) measurements were conducted on a Xenocs Xeuss 2.0-SAXS/WAXS system with X-ray wavelength of 1.5418 Å. Thermogravimetric Analysis (TGA) were performed on Shimadzu TG-50 thermal gravimetric analyzer with a scan rate of 10^o^C min^-1^. Differential scanning calorimetry (DSC) were conducted on a Shimadzu DSC-60Plus analyzer at a heating rate of 10^o^C min^−1^ and under a N_2_ flow rate of 100 mL min^−1^. Atomic force microscopy (AFM) measurements were performed using a Bruker AFM nanowizard 4XP instrument. These devices were characterized at room temperature under ambient conditions, using a KEITHLEY 4200A-SCS semiconductor parameter analyzer.

2. Synthetic details

Synthesis of 2a

Aluminum chloride (15.2 g, 114.4 mmol) was added to the stirred dichloromethane (DCM) (250 mL) solution of [1]benzothieno[3,2-b][1]-benzothiophene (BTBT) **1** (5 g, 20.8 mmol) at −10^o^C. Then the temperature was dropped to −84^o^C, 5-bromovaleryl chloride (20.7 g, 104 mmol) was added dropwise. The reaction temperature was set to -60°C and stirred for two hours and then raised to 10°C for an overnight reaction. After warming to room temperature, the reaction mixture was quenched with water, extracted with DCM, dried over anhydrous Na_2_SO_4_ and concentrated to give the residue. The residue was purified by recrystallization with hot toluene, and dried under vacuum to give yellow crystal **2a** (6.1 g, 52％).

^1^H NMR (CDCl_3_, 400 MHz) δ 8.58 (s, 2H), 8.09 (d, *J* = 8.0 Hz, 2H), 7.99 (d, *J* = 8.0 Hz, 2H), 3.49 (t, *J* = 8.0 Hz, 4H), 3.14 (t, *J* = 8.0 Hz, 4H), 2.01-1.99 (m, 8H). ^13^C NMR (CDCl_3_, 100 MHz) δ 198.47, 142.88, 136.37, 135.97, 134.05, 124.81, 124.60, 122.01, 37.70, 33.28, 32.20, 22.87. HRMS: [M+H]+ calcd. For C_24_H_22_Br_2_O_2_S_2_ 564.9506, found 566.0633.

Synthesis of 2b

Aluminum chloride (15.2 g，114.4 mmol) was added to the stirred DCM (250 mL) solution of BTBT **1** (5 g，20.8 mmol) at −10^o^C. Then the temperature was dropped to −84^o^C, 6-bromohexanoyl chloride (22.2 g, 104 mmol) was added dropwise. The reaction temperature was set to -60°C and stirred for two hours and then raised to 10°C for an overnight reaction. After warming to room temperature, the reaction mixture was quenched with water, extracted with DCM, dried over anhydrous Na_2_SO_4_ and concentrated to give the residue. The residue was purified by recrystallization with hot toluene, and dried under vacuum to give yellow crystal **2b** (7.4 g, 60％).

^1^H NMR (CDCl_3_, 400 MHz) δ 8.55 (s, 2H), 8.07 - 7.95 (m, 4H), 3.60 - 3.10 (m, 8H), 1.96 - 1.59 (m, 12H). ^13^C NMR (CDCl_3_, 100 MHz) δ 198.84, 142.82, 136.29, 135.88, 134.13, 124.79, 124.54, 121.93. HRMS: [M+H]+ calcd. For C_26_H_26_Br_2_O_2_S_2_ 592.9819, found 592.1032.

Synthesis of 3a

Compound **2a** (3 g, 5.3 mmol) was added to tetrahydrofuran (THF) (75 mL) under nitrogen atmosphere, followed by the addition of sodium borohydride (2.0 g, 53 mmol) and aluminum chloride (3.5 g, 26.5 mmol) at 0 °C to obtain the mixture, which was heated to 90 °C and reacted at reflux for 12 hours. After cooling to room temperature, the reaction solution was poured into 300 mL of DCM and 150 mL of deionized water, and the reaction mixture was extracted with DCM (100 mL × 3). The combined organic phases were dried over anhydrous Na_2_SO_4_, and concentrated under vacuum. The residue was purified on a silica gel column (DCM: Hexane = 1: 5 to 1: 1) to afford **3a** as a white powder (2.0 g, 70%).

^1^H NMR (CDCl_3_, 400 MHz) δ 7.76 (d, *J* = 8.0 Hz, 2H), 7.70 (s, 2H), 7.26 (d, *J* = 8.0 Hz, 2H), 3.41 (t, *J* = 8.0 Hz, 4H), 2,77 (t, *J* = 8.0 Hz, 4H), 1.95-1.87 (m, 4H), 1.77-1.69 (m, 4H), 1.56-1.48 (m, 4H). ^13^C NMR (CDCl_3_, 100 MHz) δ 142.47, 139.43, 132.63, 131.31, 125.78, 123.35, 121.19, 35.88, 33.76, 32.69, 30.82, 27.82. HRMS: [M+H]+ calcd. For C_24_H_26_Br_2_S_2_ 536.9921, found 538.0633.

Synthesis of 3b

Compound **2b** (3 g, 5 mmol) was added to tetrahydrofuran (THF) (75 mL) under nitrogen atmosphere, followed by the addition of sodium borohydride (1.9 g, 50 mmol) and aluminum chloride (3.4 g, 25 mmol) at 0 °C to obtain the mixture, which was heated to 90 °C and reacted at reflux for 12 hours. After cooling to room temperature, the reaction solution was poured into 300 mL of DCM and 150 mL of deionized water, and the reaction mixture was extracted with DCM (100 mL × 3). The combined organic phases were dried over anhydrous Na_2_SO_4_, and concentrated under vacuum. The residue was purified on a silica gel column (DCM: Hexane = 1: 5 to 1: 1) to afford **3b** as a white powder (2.2 g, 75%).

^1^H NMR (CDCl_3_, 400 MHz) δ 7.76 (d, *J* = 8.0 Hz, 2H), 7.70 (s, 2H), 7.26 (d, *J* = 8.0 Hz, 2H), 3.40 (t, *J* = 8.0 Hz, 4H), 2,76 (t, *J* = 8.0 Hz, 4H), 1.90-1.83 (m, 4H), 1.75-1.68 (m, 4H), 1.53-1.36 (m, 8H). ^13^C NMR (CDCl_3_, 100 MHz) δ 142.44, 139.68, 132.59, 131.26, 125.80, 123.35, 121.14, 35.95, 33.96, 32.72, 31.45, 28.36, 28.03. HRMS: [M+H]+ calcd. For C_26_H_30_Br_2_S_2_ 565.0234, found 566.1167.

Synthesis of 4a

Trimethylsilylacetylene (913.4 mg, 9.3 mmol) was dissolved in THF (20 mL), cooled down to -78°C, stirred for 5 min, then n-butyllithium (1.6 M hexane solution, 5.8 mL, 9.3 mmol) was added dropwise over a period of 20 min and stirred for 1 h at -78°C, followed by the addition of hexamethylphosphoramide (1.6 g, 9.3 mmol). Finally, a THF (20 mL) solution of **3a** (1 g, 1.86 mmol) was added dropwise to the reaction mixture at -78°C, stirred at this temperature for 10 min and then at room temperature for 16 hours. The reaction was quenched with saturated aqueous ammonium chloride solution (50 mL) and the aqueous phase was extracted with DCM (100 mL × 3). The combined organic phases were dried over anhydrous Na_2_SO_4_, and concentrated under vacuum. The residue was purified on a silica gel column (Ethyl acetate: Hexane = 1: 50) to afford **4a** as a white powder (287 mg, 27%).

^1^H NMR (CDCl_3_, 400 MHz) δ 7.63 (d, *J* = 8.0 Hz, 2H), 7.57 (s, 2H), 7.14 (d, *J* = 8.0 Hz, 2H), 2.64 (t, *J* = 8.0 Hz, 4H), 2,10 (t, *J* = 8.0 Hz, 4H), 1.62-1.55 (m, 4H), 1.48-1.41 (m, 4H), 1.38-1.30 (m, 4H), 0 (m, 18H). ^13^C NMR (CDCl_3_, 100 MHz) δ 142.24, 139.55, 132.39, 131.06, 125.62, 123.16, 120.94, 107.29, 84.33, 35.78, 30.98, 28.32, 28.26, 19.64. HRMS: [M-H]- calcd. For C_34_H_44_S_2_Si_2_ 571.2345, found 571.2318.

Synthesis of 4b

Trimethylsilylacetylene (884 mg, 9.0 mmol) was dissolved in THF (20 mL), cooled down to -78°C, stirred for 5 min, then n-butyllithium (1.6 M hexane solution, 5.6 mL, 9.0 mmol) was added dropwise over a period of 20 min and stirred for 1 h at -78°C, followed by the addition of hexamethylphosphoramide (1.6 g, 9.0 mmol). Finally, a THF (20 mL) solution of **3b** (1 g, 1.80 mmol) was added dropwise to the reaction mixture at -78°C, stirred at this temperature for 10 min and then at room temperature for 16 hours. The reaction was quenched with saturated aqueous ammonium chloride solution (50 mL) and the aqueous phase was extracted with DCM (100 mL × 3). The combined organic phases were dried over anhydrous Na_2_SO_4_, and concentrated under vacuum. The residue was purified on a silica gel column (Ethyl acetate: Hexane = 1: 50) to afford **4b** as a white powder (254 mg, 24%).

^1^H NMR (CDCl_3_, 400 MHz) δ 7.60 (d, *J* = 8.0 Hz, 2H), 7.54 (s, 2H), 7.10 (d, *J* = 8.0 Hz, 2H), 2.60 (t, *J* = 8.0 Hz, 4H), 2,07 (t, *J* = 8.0 Hz, 4H), 1.59-1.51 (m, 4H), 1.40-1.21 (m, 12H), 0 (m, 18H). ^13^C NMR (CDCl_3_, 100 MHz) δ 142.39, 139.82, 132.53, 131.19, 125.77, 123.29, 121.05, 107.59, 84.37, 35.97, 31.50, 28.68, 28.59, 28.51, 19.83. HRMS: [M]+ calcd. For C_36_H_48_S_2_Si_2_ 600.2736, found 600.5810.

Synthesis of 5a

To a solution of methanol (5 mL) containing **4a** (200 mg, 0.35 mmol) was added anhydrous potassium carbonate (242 mg, 1.75 mmol). The reaction mixture was stirred at 40 °C overnight. The mixture was poured into deionized water (20 mL), and the aqueous phase was extracted with DCM (100 mL × 3). The combined organic phases were dried over anhydrous Na_2_SO_4_, and concentrated under vacuum. The residue was purified by recrystallization with solvent mixture of DCM and hexane, and dried under vacuum to give white crystal **5a** (134 mg, 90％).

^1^H NMR (CDCl_3_, 400 MHz) δ 7.76 (d, *J* = 8.0 Hz, 2H), 7.70 (s, 2H), 7.26 (d, *J* = 8.0 Hz, 2H), 2.77 (t, *J* = 8.0 Hz, 4H), 2,20 (td, *J* = 8.0 Hz, 4H), 1.95 (t, *J* = 4.0 Hz, 2H), 1.76-1.68 (m, 4H), 1.63-1.47 (m, 8H). ^13^C NMR (CDCl_3_, 100 MHz) δ 142.43, 139.68, 132.58, 131.25, 125.80, 123.35, 121.13, 84.57, 68.28, 35.93, 31.16, 28.37, 28.35, 18.37. HRMS: [M-H]- calcd. For C_28_H_28_S_2_ 427.1554, found 427.2353.

Synthesis of 5b

To a solution of methanol (5 mL) containing **4b** (200 mg, 0.33 mmol) was added anhydrous potassium carbonate (228 mg, 1.65 mmol). The reaction mixture was stirred at 40 °C overnight. The mixture was poured into deionized water (20 mL), and the aqueous phase was extracted with DCM (100 mL × 3). The combined organic phases were dried over anhydrous Na_2_SO_4_, and concentrated under vacuum. The residue was purified by recrystallization with solvent mixture of DCM and hexane, and dried under vacuum to give white crystal **5b** (139 mg, 92％).

^1^H NMR (CDCl_3_, 400 MHz) δ 7.76 (d, *J* = 8.0 Hz, 2H), 7.70 (s, 2H), 7.26 (d, *J* = 8.0 Hz, 2H), 2.76 (t, *J* = 8.0 Hz, 4H), 2.19 (td, *J* = 8.0 Hz, 4H), 1.95 (t, *J* = 4.0 Hz, 2H), 1.75-1.67 (m, 4H), 1.57-1.36 (m, 12H). ^13^C NMR (CDCl_3_, 100 MHz) δ 142.42, 139.84, 132.56, 131.22, 125.81, 123.34, 121.10, 84.67, 68.18, 36.02, 31.53, 28.72, 28.58, 28.39, 18.40. HRMS: [M]+ calcd. For C_30_H_32_S_2_ 456.1945, found 456.4349.

Synthesis of 6

To a solution of pyrrolidine (10 mL) containing **5a** (100 mg, 0.23 mmol) at 0^o^C was added cuprous iodide (11 mg, 0.058 mmol). 1-Iodopropyne (191 mg,1.15 mmol) was added dropwise to the mixture and stirred for 12 h at room temperature under nitrogen atmosphere. Quenching the reaction with saturated aqueous ammonium chloride solution (20 mL), and the aqueous phase was extracted with DCM (20 mL × 3). The combined organic phases were dried over anhydrous Na_2_SO_4_, and concentrated under vacuum. The residue was purified on a silica gel column (Ethyl acetate: Hexane = 1: 50) to afford **6** as a white powder (45.6 mg, 39%).

^1^H NMR (CDCl_3_, 400 MHz) δ 7.76 (d, *J* = 8.0 Hz, 2H), 7.70 (s, 2H), 7.26 (d, *J* = 8.0 Hz, 2H), 2.76 (t, *J* = 8.0 Hz, 4H), 2.26 (t, *J* = 8.0 Hz, 4H), 1.90 (s, 6H), 1.74-1.67 (m, 4H), 1.60-1.54 (m, 4H), 1.52-1.44 (m, 4H). ^13^C NMR (CDCl_3_, 100 MHz) δ 142.44, 139.64, 132.59, 131.26, 125.80, 123.34, 121.13, 73.12, 65.48, 64.52, 35.90, 31.13, 28.41, 28.17, 19.11, 4.19. HRMS: [M]+ calcd. For C_34_H_32_S_2_ 504.1945, found 504.4613. UV/vis/NIR (DCM) λ_max_ nm (ɛ): 241 (26327), 270 (29931), 313 (30145).

Synthesis of 7

To a solution of pyrrolidine (10 mL) containing **5a** (100 mg, 0.23 mmol) at 0^o^C was added cuprous iodide (11 mg, 0.058 mmol). 1-Iodo-1-pentyne (223 mg,1.15 mmol) was added dropwise to the mixture and stirred for 12 h at room temperature under nitrogen atmosphere. Quenching the reaction with saturated aqueous ammonium chloride solution (20 mL), and the aqueous phase was extracted with DCM (20 mL × 3). The combined organic phases were dried over anhydrous Na_2_SO_4_, and concentrated under vacuum. The residue was purified on a silica gel column (Ethyl acetate: Hexane = 1: 50) to afford **7** as a white powder (46.2 mg, 35%).

^1^H NMR (CDCl_3_, 400 MHz) δ 7.76 (d, *J* = 8.0 Hz, 2H), 7.70 (s, 2H), 7.26 (d, *J* = 8.0 Hz, 2H), 2.76 (t, *J* = 8.0 Hz, 4H), 2.28-2.21 (m, 8H), 1.75-1.67 (m, 4H), 1.60-1.48 (m, 12H), 0.98 (t, *J* = 8.0 Hz, 6H). ^13^C NMR (CDCl_3_, 100 MHz) δ 142.44, 139.64, 132.59, 131.26, 125.80, 123.34, 121.13, 65.46, 65.35, 35.90, 31.15, 28.44, 28.19, 21.85, 21.20, 19.17, 13.50. HRMS: [M]+ calcd. For C_38_H_40_S_2_ 560.2571, found 560.5126. UV/vis/NIR (DCM) λ_max_ nm (ɛ): 241 (20499), 270 (23300), 313 (23889).

Synthesis of 8

To a solution of pyrrolidine (10 mL) containing **5b** (100 mg, 0.22 mmol) at 0^o^C was added cuprous iodide (10.5 mg, 0.055 mmol). 1-Iodo-1-pentyne (213 mg,1.10 mmol) was added dropwise to the mixture and stirred for 12 h at room temperature under nitrogen atmosphere. Quenching the reaction with saturated aqueous ammonium chloride solution (20 mL), and the aqueous phase was extracted with DCM (20 mL × 3). The combined organic phases were dried over anhydrous Na_2_SO_4_, and concentrated under vacuum. The residue was purified on a silica gel column (Ethyl acetate: Hexane = 1: 50) to afford **8** as a white powder (45.7 mg, 36%).

^1^H NMR (CDCl_3_, 400 MHz) δ 7.76 (d, *J* = 8.0 Hz, 2H), 7.70 (s, 2H), 7.26 (d, *J* = 8.0 Hz, 2H), 2.75 (t, *J* = 8.0 Hz, 4H), 2.27-2.21 (m, 8H), 1.74-1.66 (m, 4H), 1.57-1.41 (m, 16H), 0.98 (t, *J* = 8.0 Hz, 6H). ^13^C NMR (CDCl_3_, 100 MHz) δ 142.42, 139.82, 132.56, 131.22, 125.80, 123.34, 121.10, 65.38, 36.00, 31.50, 28.69, 28.64, 28.23, 21.85, 21.21, 19.19, 13.50. HRMS: [M]+ calcd. For C_40_H_44_S_2_ 588.2884, found 588.5895. UV/vis/NIR (DCM) λ_max_ nm (ɛ): 241 (23194), 270 (26291), 313 (26536).

3. NMR and MALDI-TOF-MS spectra

**Figure S1**. ^1^H NMR spectrum of **2a** in CDCl_3_.

**Figure S2**. ^13^C NMR spectrum of **2a** in CDCl_3_.

**Figure S3**. ^1^H NMR spectrum of **2b** in CDCl_3_.

**Figure S4**. ^13^C NMR spectrum of **2b** in CDCl_3_.

**Figure S5**. ^1^H NMR spectrum of **3a** in CDCl_3_.

**Figure S6**. ^13^C NMR spectrum of **3a** in CDCl_3_.

**Figure S7**. ^1^H NMR spectrum of **3b** in CDCl_3_.

**Figure S8**. ^13^C NMR spectrum of **3b** in CDCl_3_.

**Figure S9**. ^1^H NMR spectrum of **4a** in CDCl_3_.

**Figure S10**. ^13^C NMR spectrum of **4a** in CDCl_3_.

**Figure S11**. ^1^H NMR spectrum of **4b** in CDCl_3_.

**Figure S12**. ^13^C NMR spectrum of **4b** in CDCl_3_.

**Figure S13**. ^1^H NMR spectrum of **5a** in CDCl_3_.

**Figure S14**. ^13^C NMR spectrum of **5a** in CDCl_3_.

**Figure S15**. ^1^H NMR spectrum of **5b** in CDCl_3_.

**Figure S16**. ^13^C NMR spectrum of **5b** in CDCl_3_.

**Figure S17**. ^1^H NMR spectrum of **6** in CDCl_3_.

**Figure S18**. ^13^C NMR spectrum of **6** in CDCl_3_.

**Figure S19**. ^1^H NMR spectrum of **7** in CDCl_3_.

**Figure S20**. ^13^C NMR spectrum of **7** in CDCl_3_.

**Figure S21**. ^1^H NMR spectrum of **8** in CDCl_3_.

**Figure S22**. ^13^C NMR spectrum of **8** in CDCl_3_.


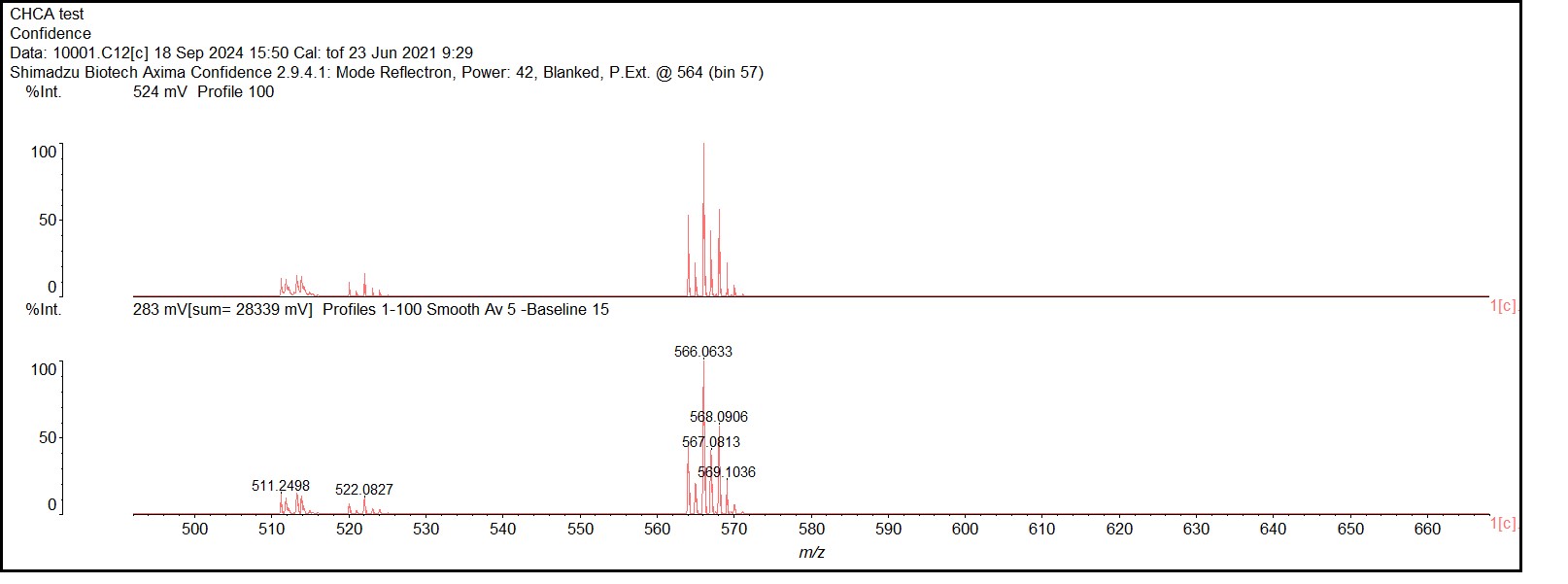


**Figure S23**. HRMS spectrum of **2a**.


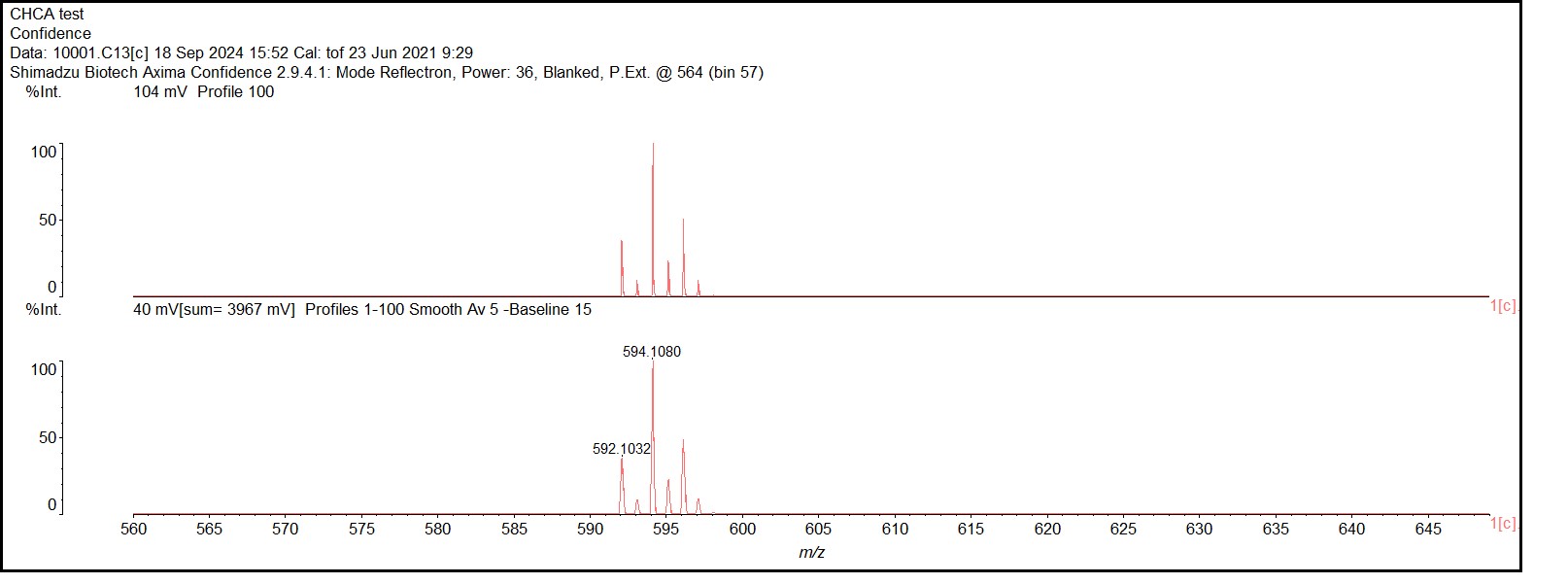


**Figure S24**. HRMS spectrum of **2b**.


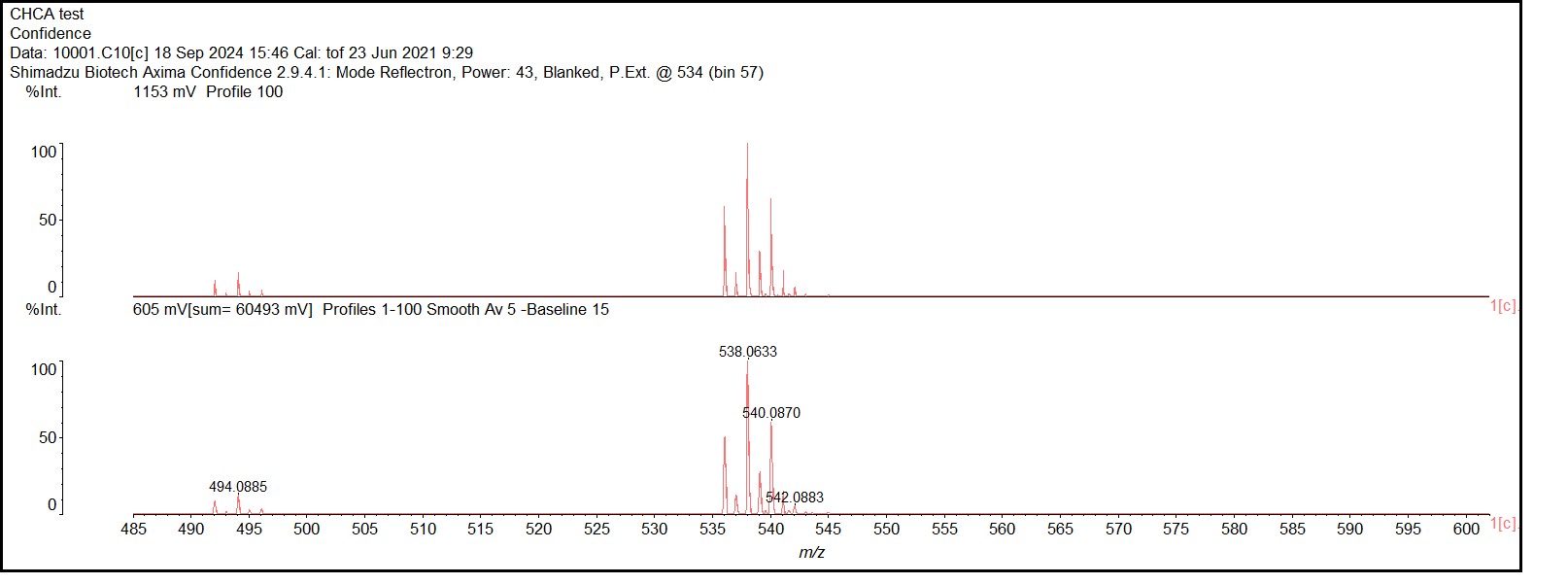


**Figure S25**. HRMS spectrum of **3a**.


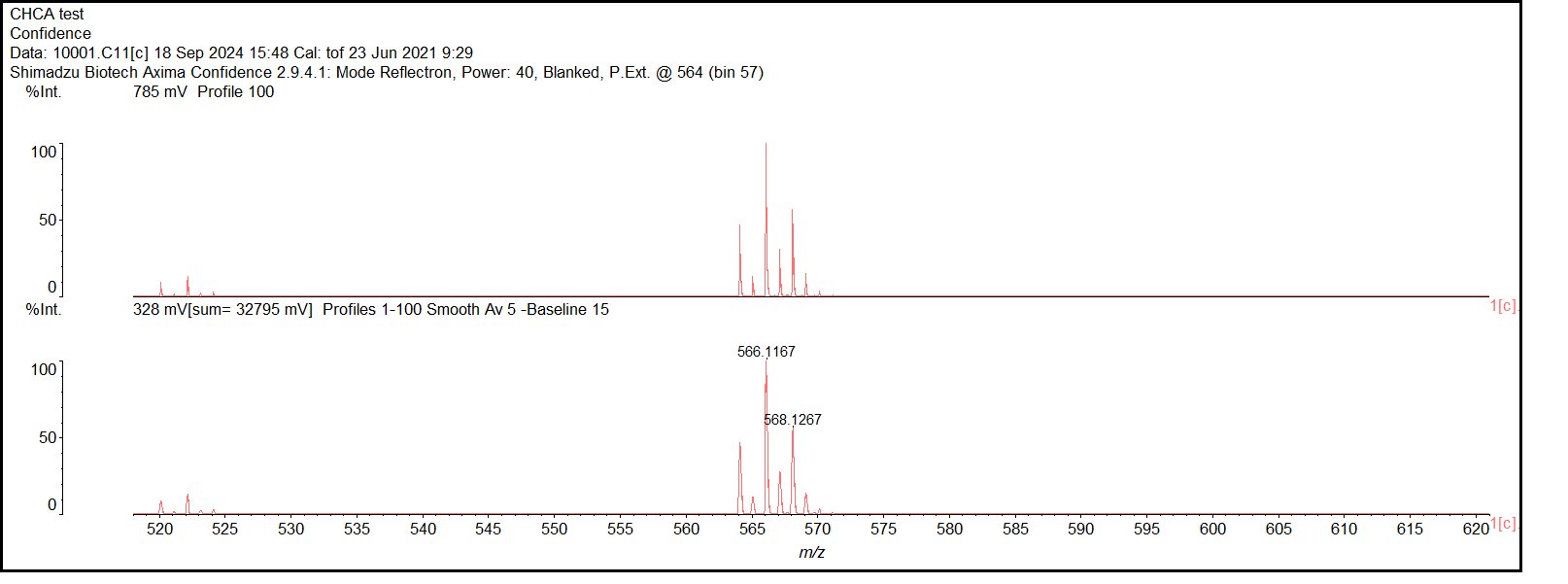


**Figure S26**. HRMS spectrum of **3b**.


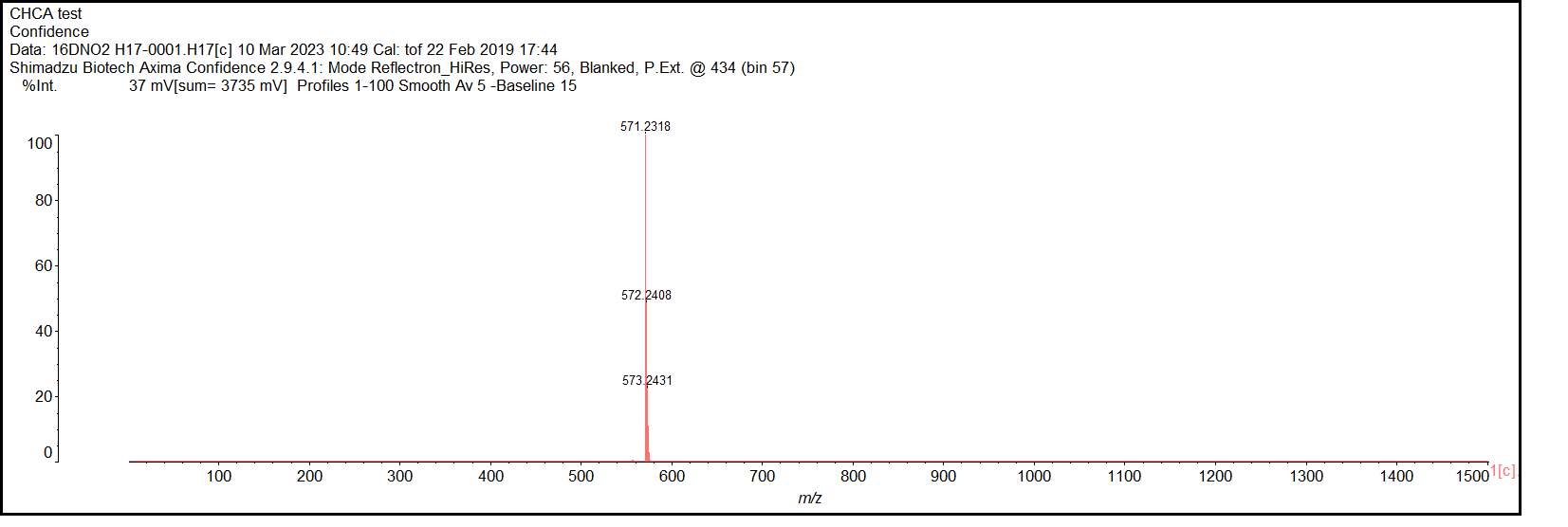


**Figure S27**. HRMS spectrum of **4a**.


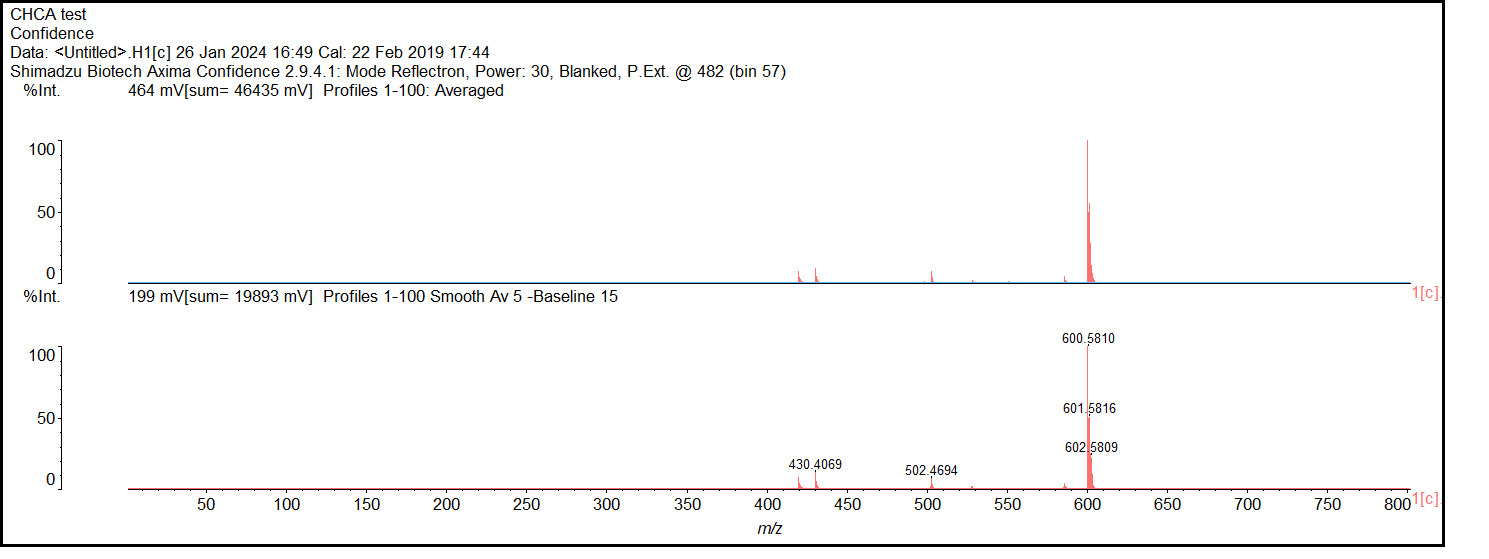


**Figure S28**. HRMS spectrum of **4b**.


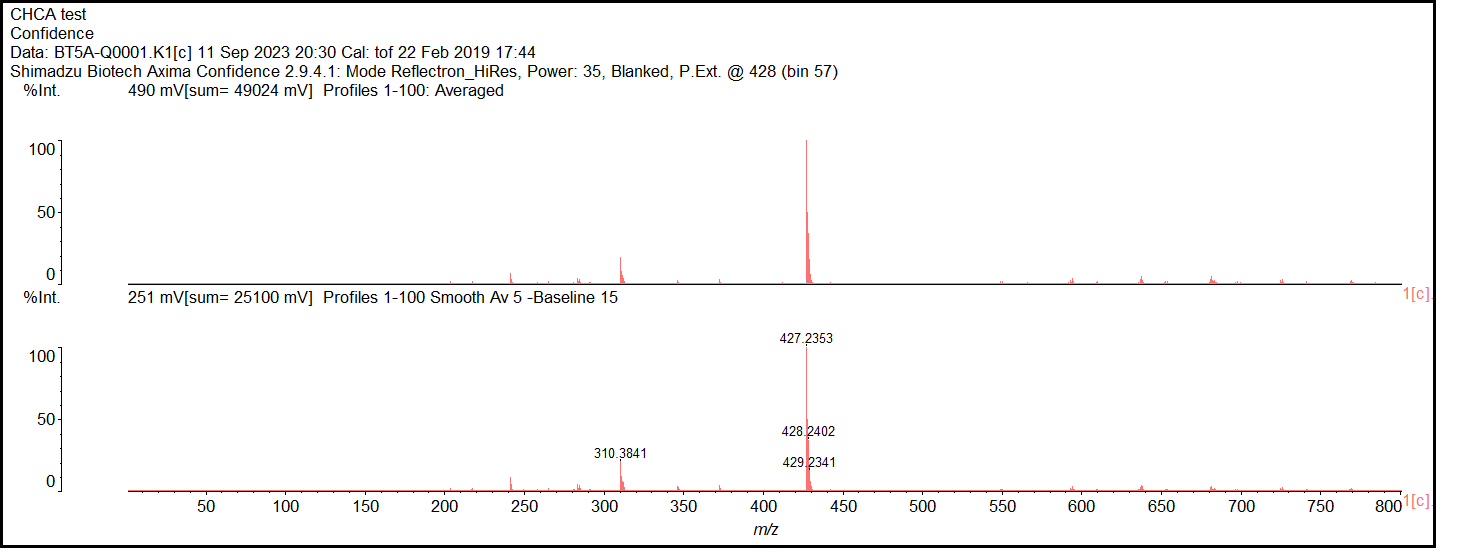


**Figure S29**. HRMS spectrum of **5a**.


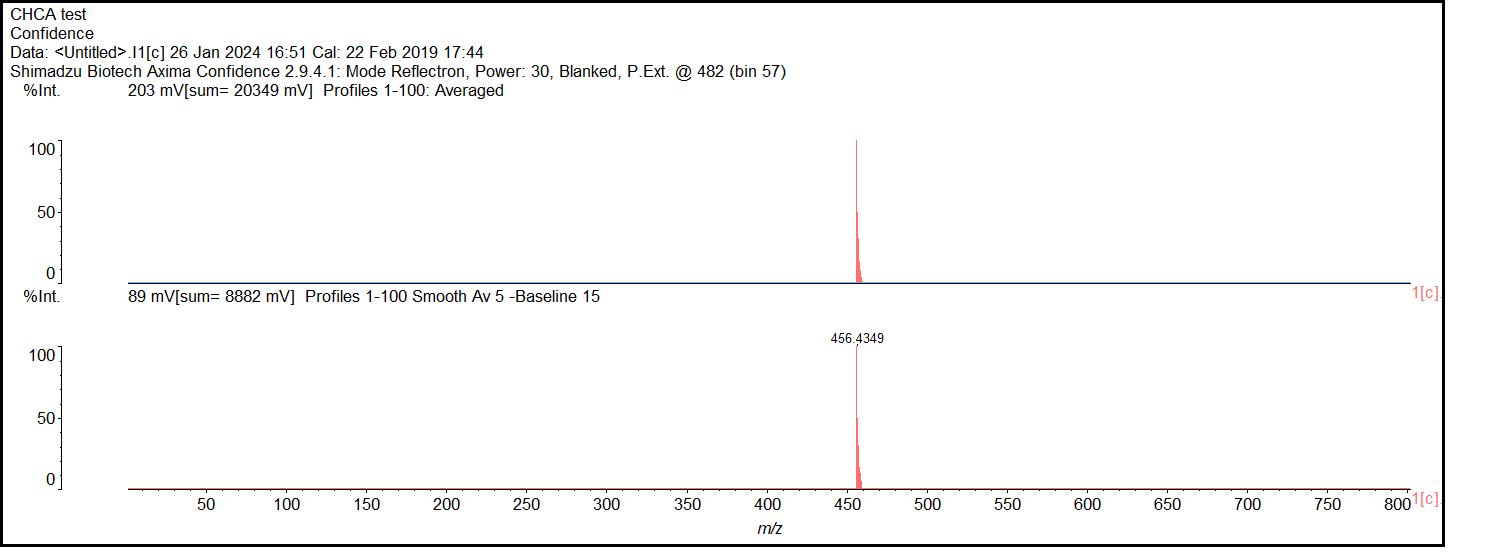


**Figure S30**. HRMS spectrum of **5b**.


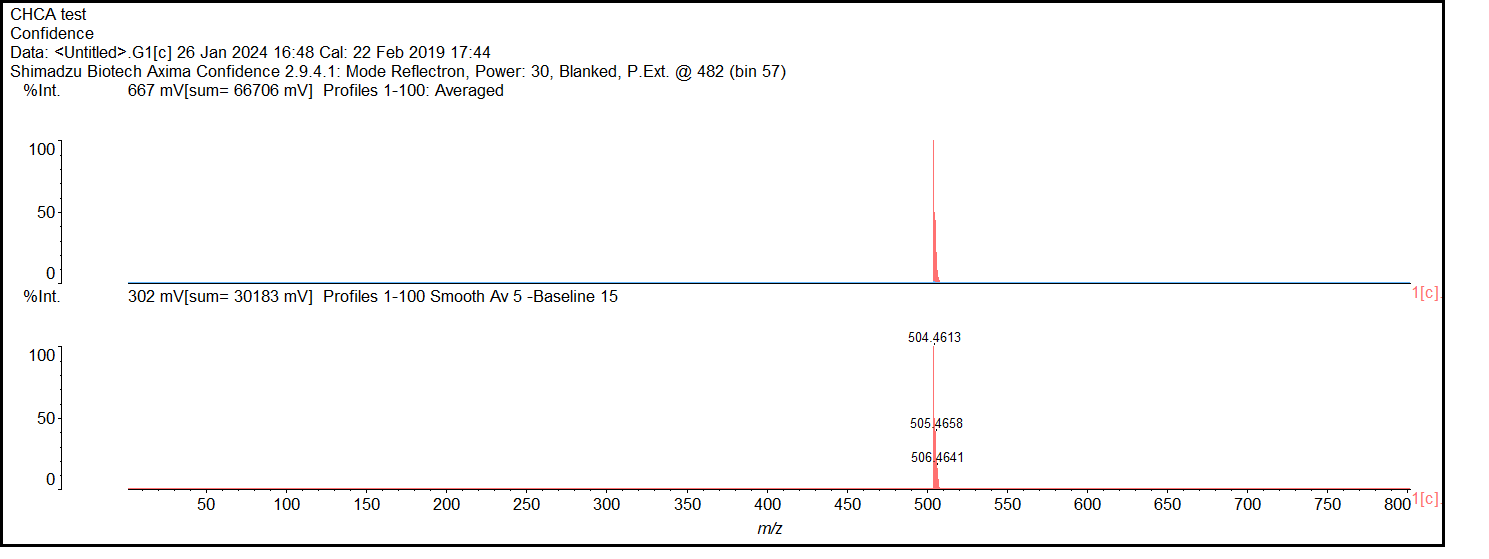


**Figure S31**. HRMS spectrum of **6**.


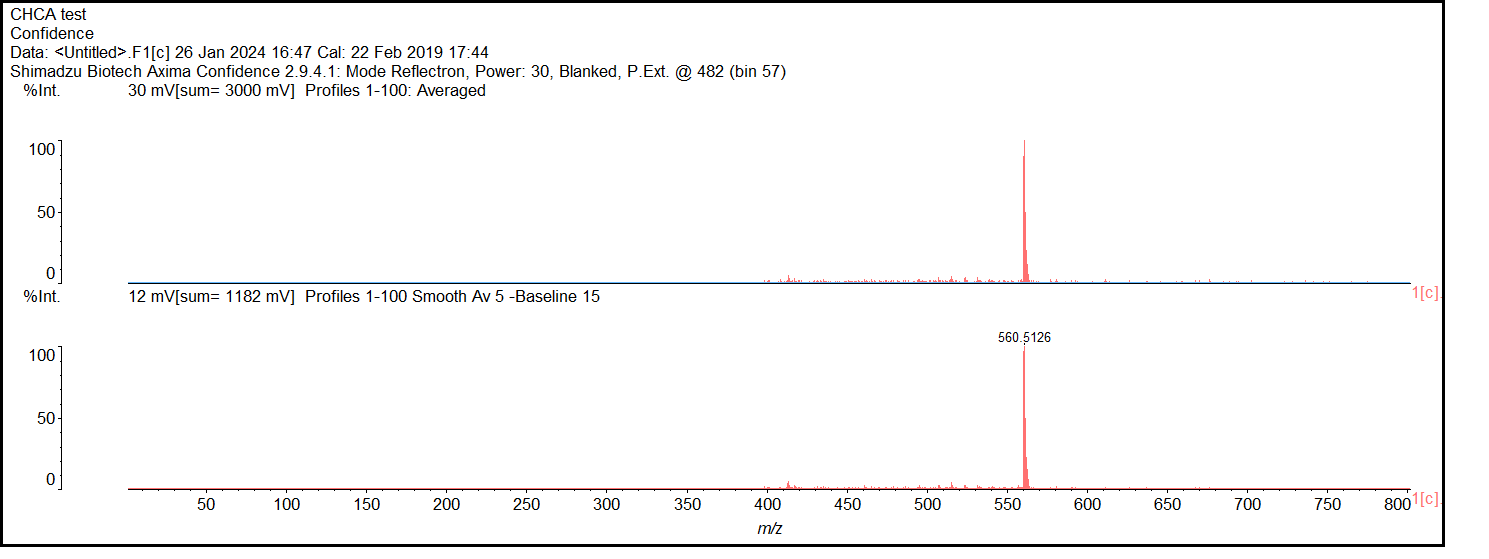


**Figure S32**. HRMS spectrum of **7**.


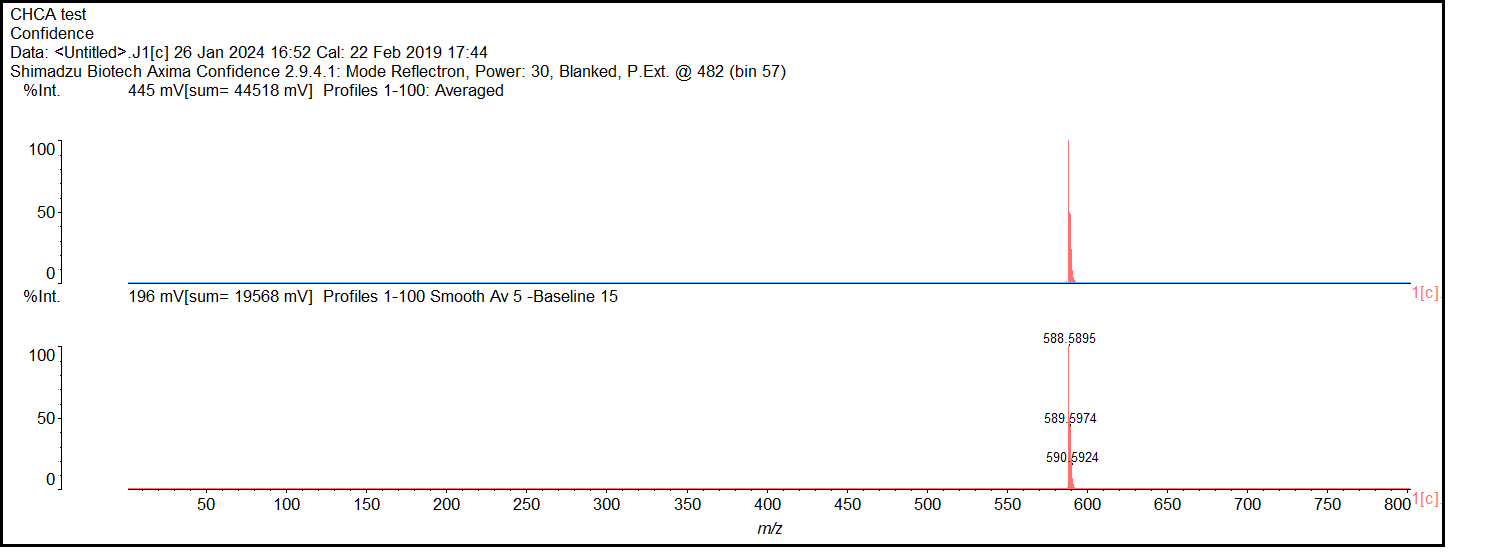


**Figure S33**. HRMS spectrum of **8**.

4. Single crystal X-ray diffraction

Single crystals were obtained by slow solvent evaporation from THF/Hexane or toluene solutions at ambient temperature. **6** and **7** form crystals with the triclinic P-1 space group, and **8** belongs to a monoclinic system with space group P2_1_/c. All three molecules exhibit layered packing, with each layer having a molecular height of 2.63, 2.77 and 1.69 nm corresponding to **6**, **7** and **8**, respectively (**Figure S34**). From the molecular crystal structure, a few molecules in close proximity were selected to evaluate the distance between S atoms. It can be seen that the S-S distance in molecule **8** (d_s-s_=5.53 Å) is greater than that in **6** and **7** molecules (d_s-s_=4.28 Å for **6**, d_s-s_=4.36 Å for **7**), and the BTBT structures of **8** molecule exhibit a sliding stacking state.

Single-crystal structural analysis reveals that compound **8** does not undergo UV-induced cross-linking due to its unfavorable molecular packing and the large separation between its reactive groups. Compared to compounds **6** and **7**, which exhibit close intermolecular distances and favorable alignment of diacetylene (C≡C–C≡C) groups for topochemical 1,4-coupling, compound **8** displays much larger intermolecular spacings (11.57 Å and 8.10 Å). More importantly, the distance between adjacent reactive carbons (C1 and C4') in the diacetylene groups of compound **8** is 4.77 Å, which greatly exceeds the distance required for efficient cross-linking. This excessive separation prevents effective orbital overlap and molecular interaction, thus inhibiting the cross-linking reaction under UV irradiation.


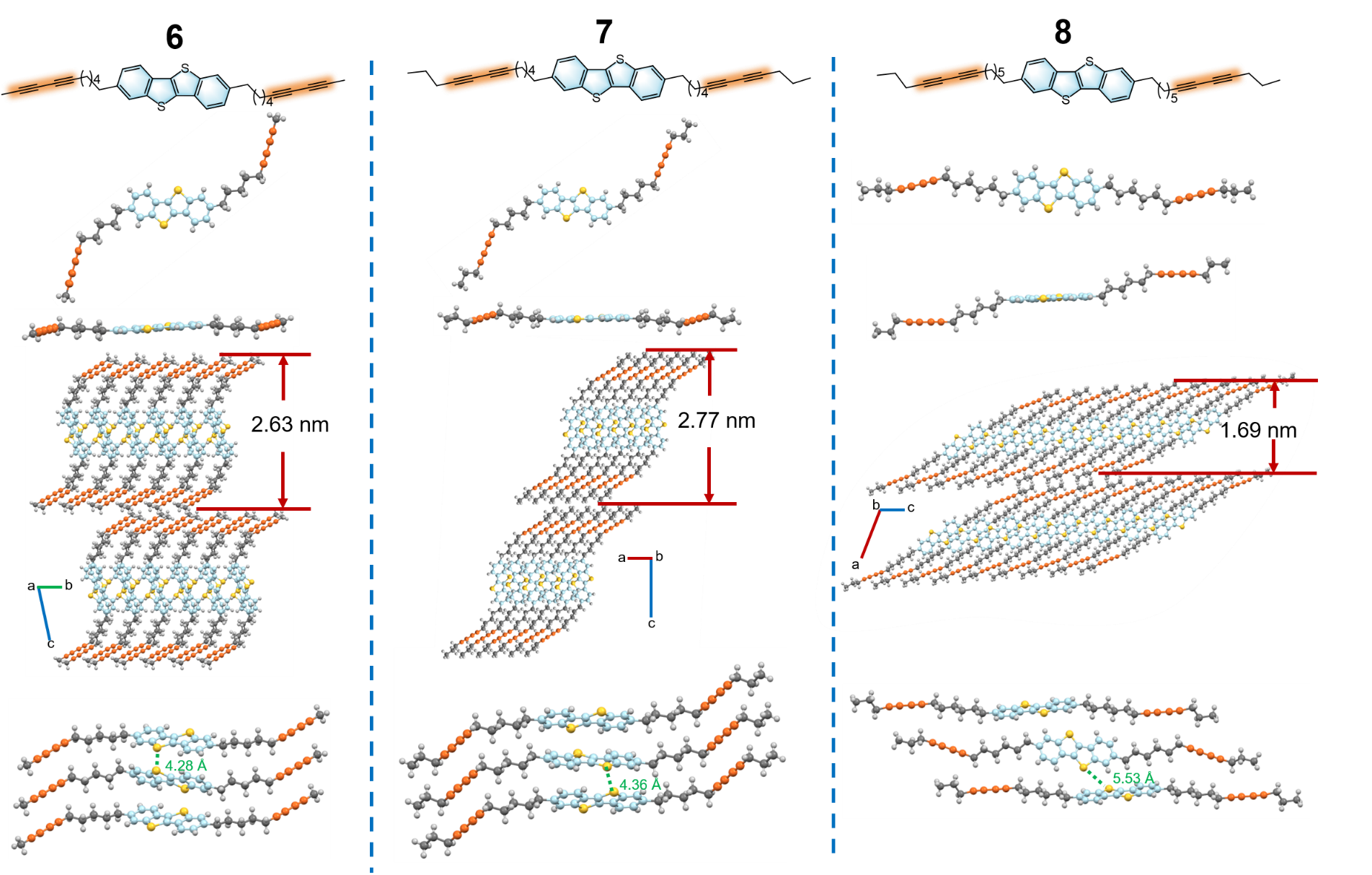


**Figure S34**. Molecular and packing structures of **6**, **7** and **8** were obtained by single-crystal X-ray diffraction.


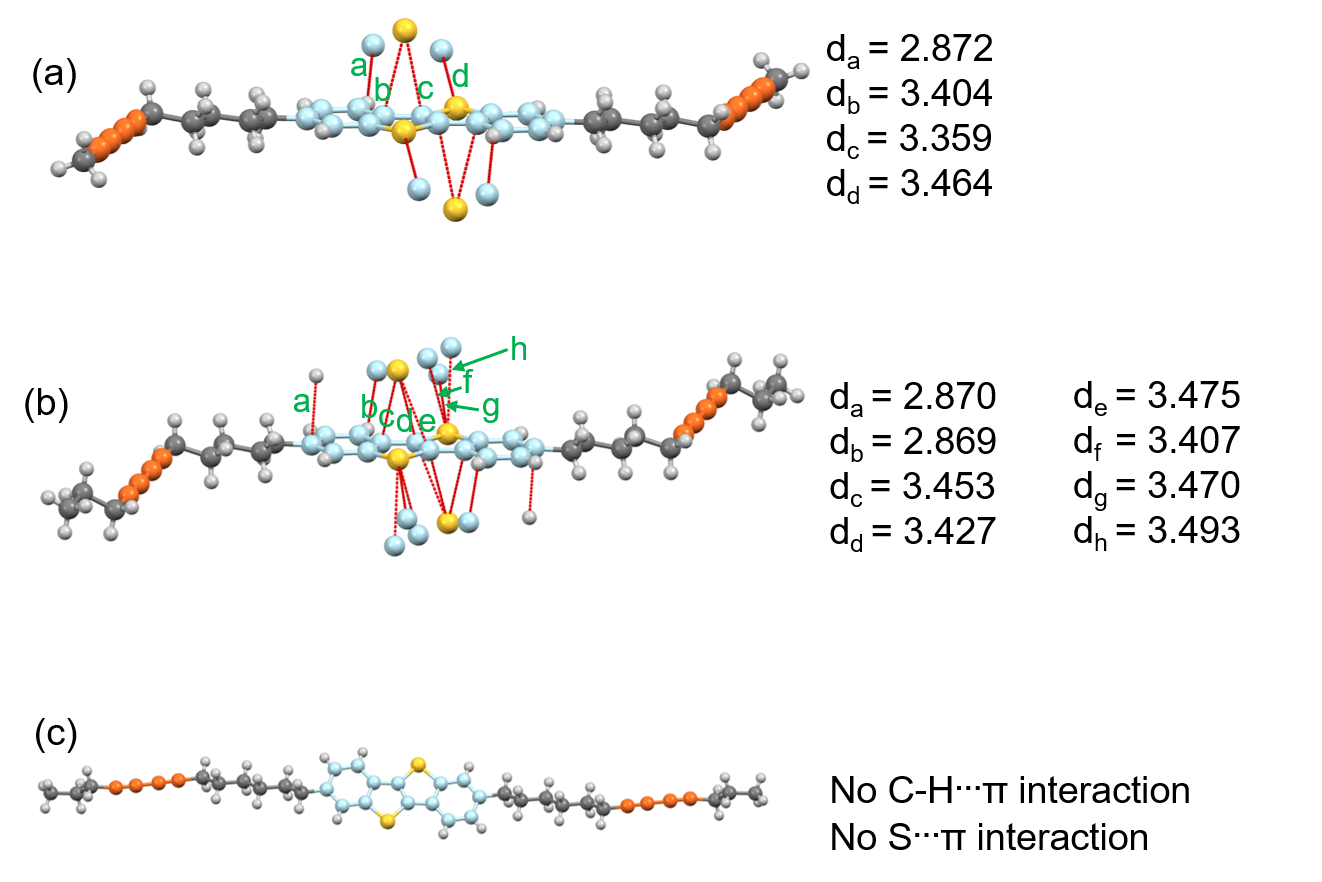


**Figure S35**. Multiple intermolecular interactions for **6** (a), **7** (b) and **8** (c). The unit of data is Å.

**Table S1.** Crystal data and structure refinement of **6**, **7** and **8**.

| Identification code | **6** | **7** | **8** |
| --- | --- | --- | --- |
|  | C_34_H_32_S_2_ | C_38_S_2_H_40_ | C_40_H_44_S_2_ |
| Formula weight | 504.71 | 560.82 | 588.87 |
| Temperature/K | 293(2) | 100 | 293(2) |
| Crystal system | triclinic | triclinic | monoclinic |
| Space group | P-1 | P-1 | P2_1_/c |
| a/Å | 10.3710(5) | 6.3998(4) | 19.1010(5) |
| b/Å | 10.6103(5) | 8.0868(5) | 8.10207(18) |
| c/Å | 27.6709(14) | 30.041(2) | 11.5671(3) |
| α/° | 79.101(4) | 89.137(3) | 90 |
| β/° | 82.019(4) | 85.725(3) | 106.386(3) |
| γ/° | 70.123(5) | 89.985(3) | 90 |
| Volume/Å^3^ | 2802.7(3) | 1550.24(17) | 1717.40(8) |
| Z | 4 | 2 | 2 |
| ρ_calc_g/cm^3^ | 1.196 | 1.201 | 1.139 |
| μ/mm^‑1^ | 1.858 | 1.133 | 1.580 |
| F(000) | 1072.0 | 600.0 | 632.0 |
| Crystal size/mm^3^ | 0.38 × 0.13 × 0.02 | 0.15 × 0.14 × 0.1 | 0.26 × 0.1 × 0.03 |
| Radiation | CuKα (λ = 1.54184) | GaKα (λ = 1.34139) | CuKα (λ = 1.54184) |
| 2Θ range for data collection/° | 8.968 to 134.16 | 5.134 to 114.92 | 9.652 to 134.134 |
| Index ranges | -11 ≤ h ≤ 12, -8 ≤ k ≤ 12, -33 ≤ l ≤ 33 | -7 ≤ h ≤ 7, -10 ≤ k ≤ 10, -37 ≤ l ≤ 37 | -22 ≤ h ≤ 22, -9 ≤ k ≤ 8, -13 ≤ l ≤ 10 |
| Reflections collected | 19959 | 24791 | 6011 |
| Independent reflections | 10020 [R_int_ = 0.0397, R_sigma_ = 0.0563] | 6097 [R_int_ = 0.0446, R_sigma_ = 0.0449] | 3048 [R_int_ = 0.0316, R_sigma_ = 0.0451] |
| Data/restraints/parameters | 10020/9/661 | 6097/0/363 | 3048/5/202 |
| Goodness-of-fit on F^2^ | 1.005 | 1.227 | 1.057 |
| Final R indexes [I>=2σ (I)] | R_1_ = 0.0832, wR_2_ = 0.2274 | R_1_ = 0.0675, wR_2_ = 0.2126 | R_1_ = 0.0752, wR_2_ = 0.1986 |
| Final R indexes [all data] | R_1_ = 0.1323, wR_2_ = 0.2885 | R_1_ = 0.0726, wR_2_ = 0.2161 | R_1_ = 0.0940, wR_2_ = 0.2244 |
| Largest diff. peak/hole / e Å^-3^ | 0.77/-0.28 | 1.10/-0.45 | 0.50/-0.26 |

5. TGA and DSC curves

The thermal stability of **6**, **7** and **8** was determined by thermogravimetric analysis (TGA). The initial amount of weight loss of 5% (Td) is high up to 453°C, 446°C and 432°C for **6**, **7** and **8**, respectively, indicating their high thermal stability (**Figure S36**). Differential scanning calorimetry (DSC) was performed under nitrogen atmosphere with a ramp-up rate of 10°C /min. Different melting points were observed at 144°C, 108°C and 136°C for **6**, **7** and **8**, respectively (**Figure S37**).

**Figure S36**. TGA curves of **6**, **7** and **8**.

**Figure S37.** DSC curves of **6**, **7** and **8**.

6. Optical absorption, electrochemical properties and DFT calculations

The optical properties of **6**, **7** and **8** were investigated by UV-Vis-NIR spectroscopy at room temperature in DCM. The absorption spectra of **6**, **7** and **8** showed the same absorption, with intense absorption bands in the UV region (< 341 nm) (**Figure S38**). The corresponding optical energy gaps *Eg*^Opt^ are 3.63 eV for **6**, **7** and **8**^[1]^.

**Figure S38**. Normalized UV/Vis absorption spectra of **6**, **7** and **8** in DCM.

**Figure S39**. Normalized UV/Vis absorption spectra of DCM solution (black) and thin film on quartz plates (red) of **6** and **8**.

Before FT-IR measurements, the three molecules were dried in vacuum at 50 ^o^C for 48 hours, and the potassium bromide (KBr) was grinded to fine powders using an agate mortar then dried in oven at 150^o^C for 4 hours. Then the mixture of small molecules (~1 mg) and KBr (~50 mg) was grinded for 2-4 minutes under an infrared lamp. Finally, thin pellets were obtained by pressing the mixture under 10 MPa for 1 minute. According to FTIR in **Figure S40** the peaks at 3018, 3041 and 3053 cm^-1^ are attributed to the aromatic ring C-H stretching vibration; the peaks at 2926, 2933, 2849, 2850 and 2855 cm^-1^are assigned to the saturated C-H stretching vibration; 2191, 2185, 2181 and 2141cm^-1^ are the characteristic absorption peaks of C≡C stretching vibration; the peaks at 1595, 1597, 1600, 1549, and 1550cm^-1^ belong to the characteristic peak of skeleton vibration of aromatic ring^[1,2]^. These results support the successful synthesis of diacetylene semiconductor molecule.


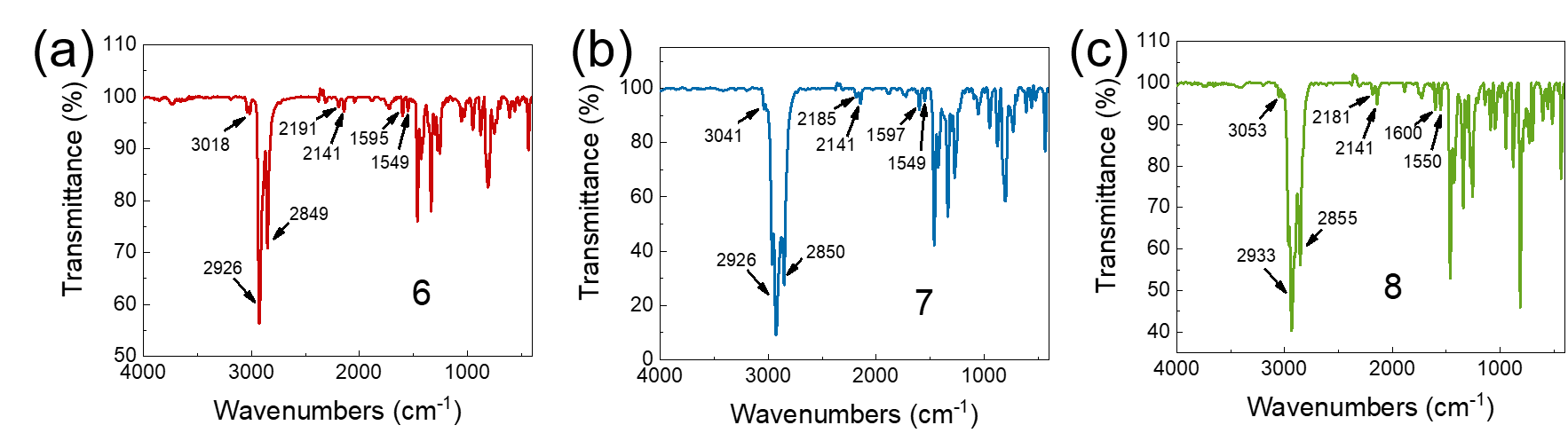


**Figure S40**. FTIR spectra of **6** (a), **7** (b) and **8** (c).

Cyclic voltammograms were measured in dry DCM with 0.1 M Bu_4_NPF_6_ as supporting electrolyte, Ag/AgCl as reference electrode, glassy carbon as working electrode, Pt wire as counter electrode, under a scan rate at 100 mV/s.

**Figure S41.** Cyclic voltammograms of ferrocene (a) and **6**, **7** and **8** (b).

All calculations were performed with the Gaussian 16 program suite^[3]^. HOMO, LUMO of **6**, **7** and **8** calculated at the UB3LYP/6-31G(d) level^[4–7]^.

|  | HOMO | LUMO |
| --- | --- | --- |
| **6** | 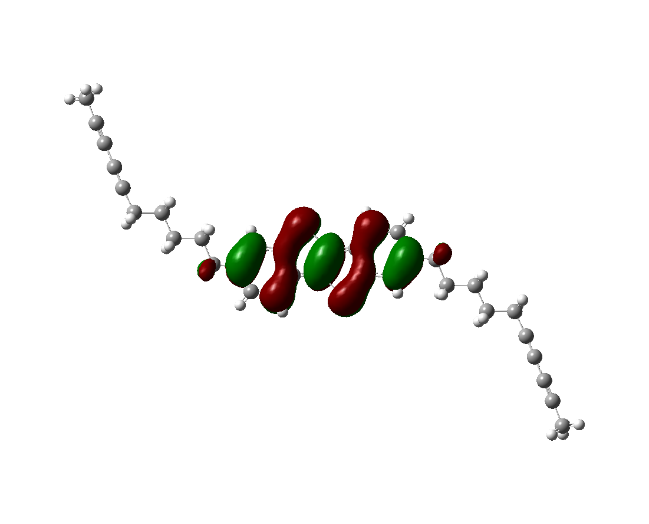 | 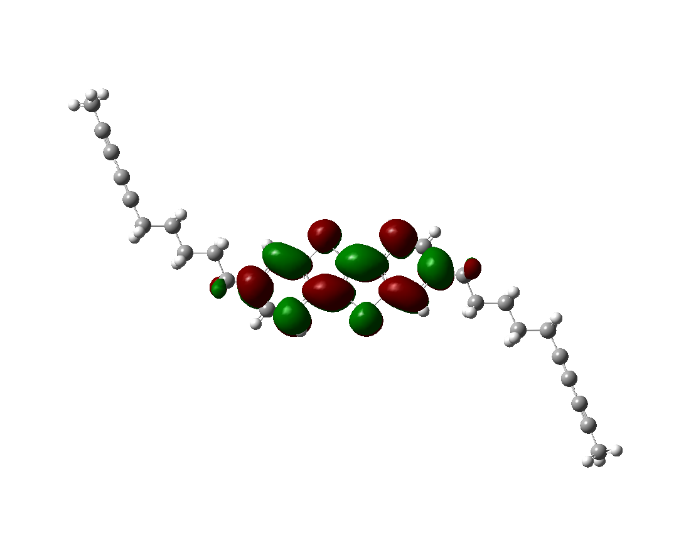 |
| **7** | 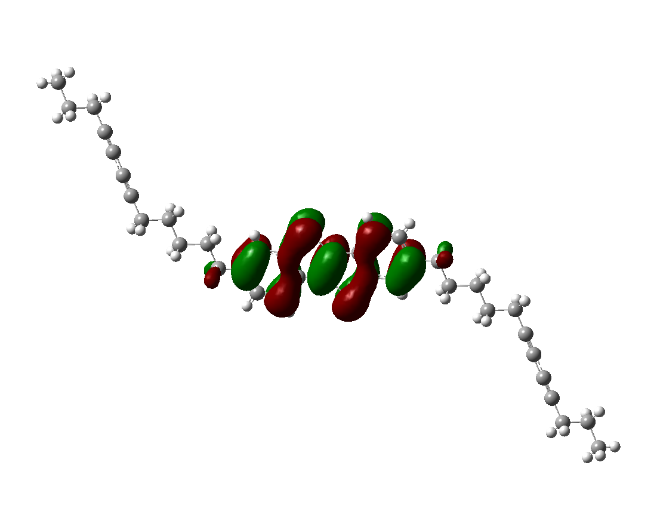 | 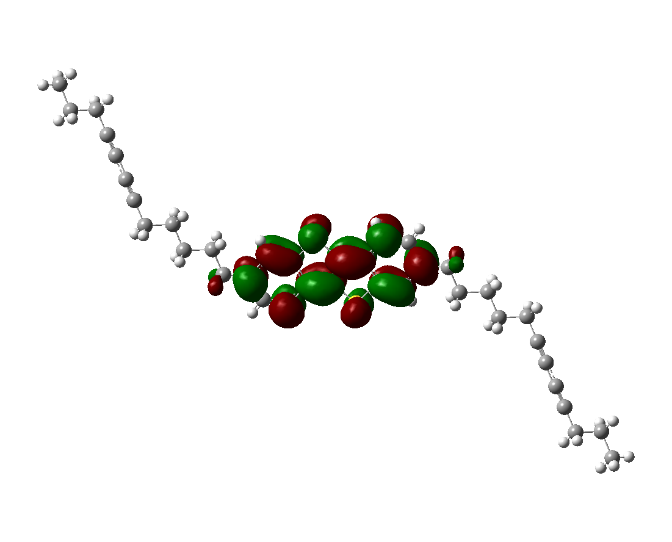 |
| **8** | 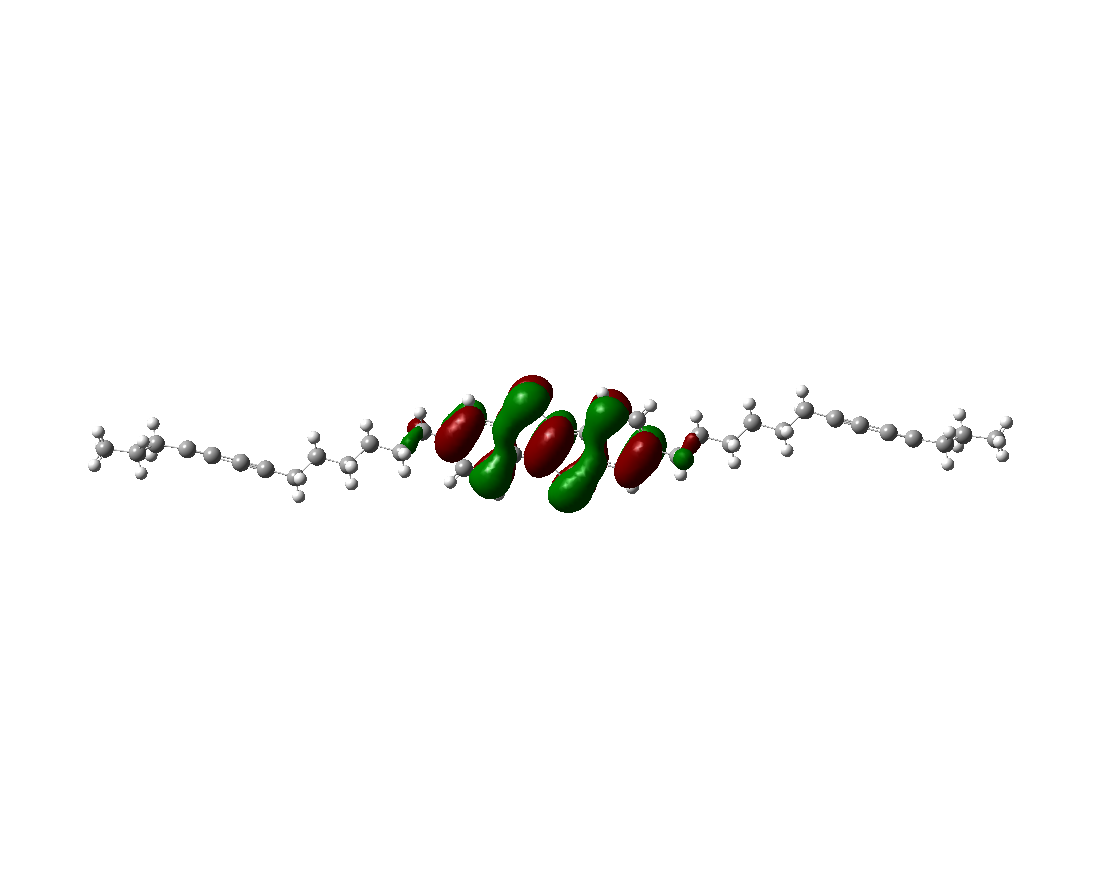 | 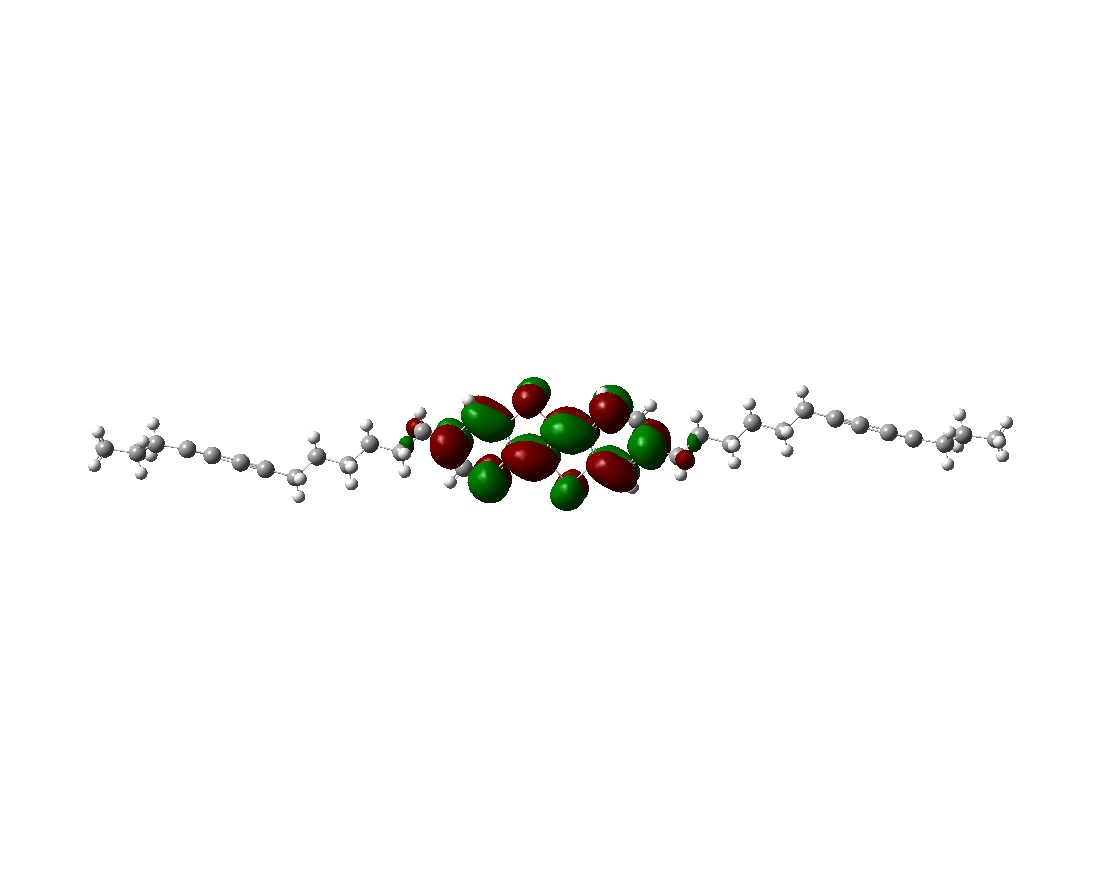 |

**Figure S42.** HOMO, LUMO of **6**, **7** and **8** calculated at the UB3LYP/6-31G(d) level.

7. Preparation and patterning of organic small molecule films

**Solubility test**

As shown in **Table S2**, compounds **6**, **7** and **8** exhibit excellent solubility in chloroform at room temperature, with values of 90, 120 and greater than 150 g/L, respectively. For comparison, the classical soluble BTBT derivative C8-BTBT shows a solubility of 80 g/L in chloroform^[8]^. The new molecules thus demonstrate solubility equal to or surpassing that of C8-BTBT, making them highly suitable for solution processing in organic electronics.

**Table S2**. Solubility of compounds **6**, **7**, **8** and C8-BTBT in chloroform at room temperature.

| Compound | Solubility in Chloroform (g/L) |
| --- | --- |
| **6** | 90 |
| **7** | 120 |
| **8** | >150 |
| C8-BTBT | 80^[8]^ |

**Preparation of films**

The SiO_2_/Si substrate used were washed successively with deionized water, acetone, isopropanol, anhydrous ethanol, and piranha solution (H_2_SO_4_: H_2_O_2_=7:3, 70^o^C, 20 min), deionized water, isopropanol and anhydrous ethanol, then dried them with nitrogen gas. Then, based on the literature, substrate PS modification was carried out^[9]^. The resulting PS-grafted SiO₂/Si substrates were used for all film deposition and device fabrication in this work.

Molecule **6** was dissolved in a mixture of toluene and chlorobenzene (toluene: chlorobenzene V:V = 4:1), and the molecule **8** was dissolved in a mixture of chloroform and chlorobenzene (chloroform: chlorobenzene V:V = 4:1) to form 2 mg/mL solutions of the small molecule semiconductors^[10,11]^. The films were formed by blade coating in ambient atmosphere (20–30°C, relative humidity ~40%), and the coating speeds for the two molecules were set to 20–200 μm/s, respectively (**Figures S46–S49**).

**Patterning of films**

The blade-coated films (prepared by solution shearing from a 2 mg/mL solution of compound **6** in toluene:chlorobenzene = 4:1 v/v onto PS-grafted SiO₂/Si substrates pre-heated to 50°C, at a shearing speed of 120 μm/s) were covered with a skeletonized mask plate and exposed to 254 nm UV light (1 mW cm⁻²). Notably, for film quality characterization, the UV exposure time was 7 min in nitrogen atmosphere, whereas for device fabrication and patterning tests, the exposure time was 1 min under the same conditions. After exposure, the films were developed by immersing in acetonitrile for 2 minutes to remove unexposed areas, followed by nitrogen drying to complete the lithography process. Due to the limitations of the mask plate machining accuracy, micron-scale patterns could not be achieved; nevertheless, the edges of the patterned films were consistent with the mask design and exhibited high clarity after rinsing (**Figure S43**, **S44**), confirming the potential of compound **6** for high-precision patterning.


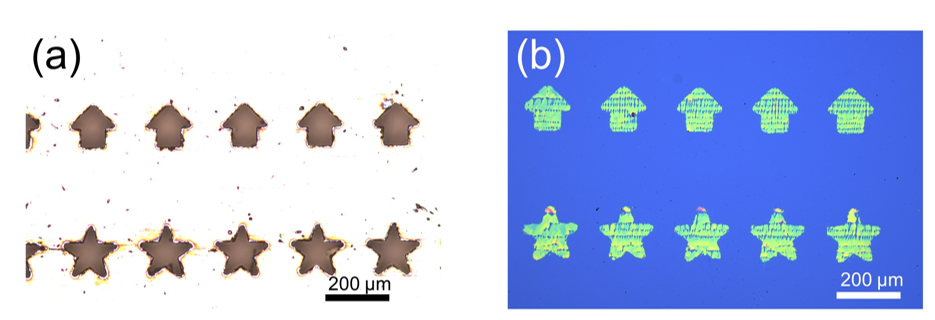


**Figure S43.** Optical microscope images of skeleton mask (a), and the corresponding patterned film **6** (b).


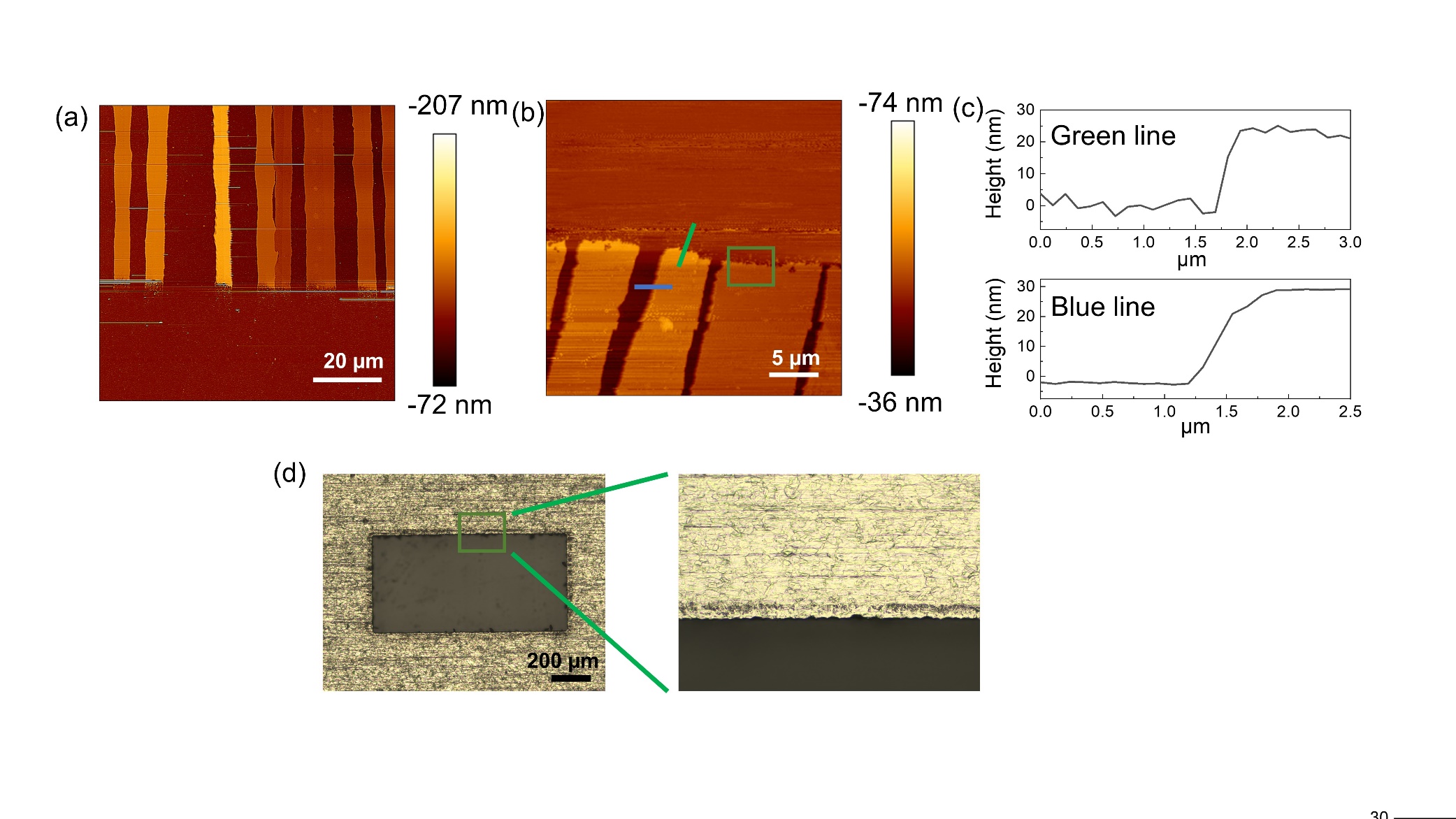


**Figure S44.** (a, b) AFM images of patterned i-PSSC film **6**, with the green box indicating the selected area for LER analysis. The average LER was calculated to be ~65 nm. (c) Height profiles taken along the green and blue lines in (b). (d) Optical images of the shadow mask used for patterning.

To compare the effects of different developing solvents, we performed a series of rinsing experiments after UV patterning. As shown in **Figure S45**, when acetonitrile was used as the rinsing solvent, the patterned regions were well preserved and the un-crosslinked areas were selectively removed, resulting in clear and high-fidelity patterns. In contrast, when chloroform or toluene was used, no patterned film remained on the substrate—both solvents completely dissolved not only the un-crosslinked molecules, but also the cross-linked regions. These results demonstrate that acetonitrile provides optimal selectivity for developing the patterned films, whereas chloroform and toluene are not suitable due to their strong solubility for both cross-linked and un-crosslinked materials.


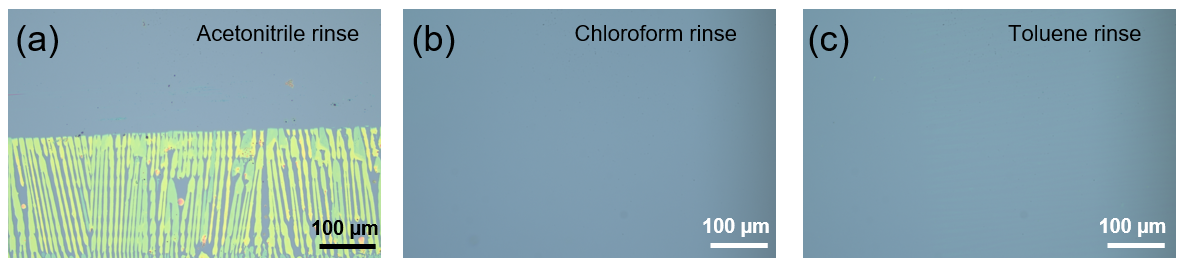


**Figure S45**. Optical microscope images of the patterned films after rinsing with different solvents: (a) acetonitrile, (b) chloroform and (c) toluene. The substrate appears as a light blue background in the optical microscopy images.

8. Film quality characterization

Organic thin films were prepared using the scraping method and the morphology and packing mode of molecules **6** and **8** were investigated at different coating speeds. At a coating speed of 120 μm/s for **6** and 140 μm/s for **8**, the films are most uniform, and at these speeds, AFM images show the lowest film thickness (~40 nm). During solution shearing, the small-molecule semiconductors exhibited anisotropic crystallization behavior, leading to the formation of ribbon-like crystalline domains aligned along the coating direction. These domains can be clearly observed in the AFM, TEM and POM images (**Figure S46-S49** and **Figure S51**, **S58**). It should be emphasized that these stripe-like features are not the result of photopatterning, but are instead formed spontaneously due to directional crystal growth during film deposition. Their presence reflects the high crystallinity and long-range molecular ordering achievable via the blade-coating process.

The thin films of **6** prepared at different coating speeds exhibit the same three diffraction peaks at 2θ = 3.26^o^，6.54^o^ and 9.81^o^，respectively, with the corresponding spacing of 27.1 Å, 13.5 Å and 9.0 Å. Similarly, the films **8** exhibit the same three diffraction peaks at 2θ=4.81^o^, 9.64^o^ and 14.49^o^, with corresponding spacing of 18.3 Å, 9.2 Å and 6.1Å. The spaces of 27.1Å and 18.3 Å are in agreement with the heights of molecular terraces (2.66, 1.65 nm for **6** and **8**, respectively) in AFM (**Figure S48c** and **Figure S49c**), which are consistent with the height of each layer molecule in the single crystals (**Figure S34**).


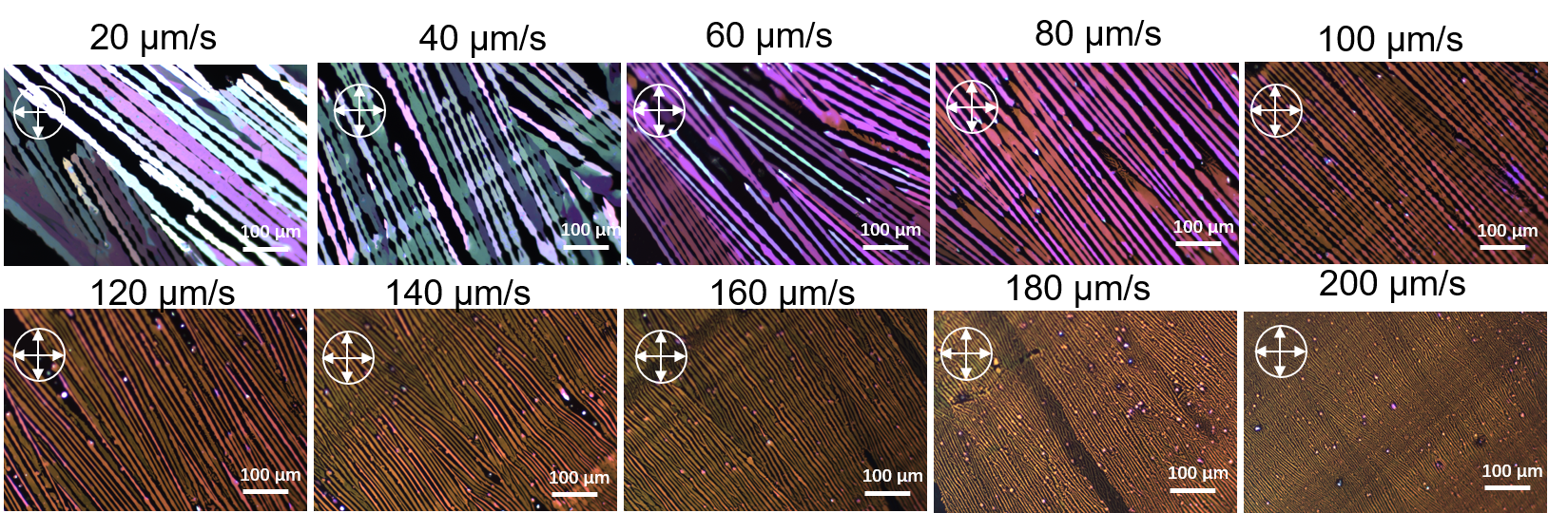


**Figure S46.** POM images of thin film **6** on SiO_2_ from mixed solution (Toluene: chlorobenzene V:V = 4:1).


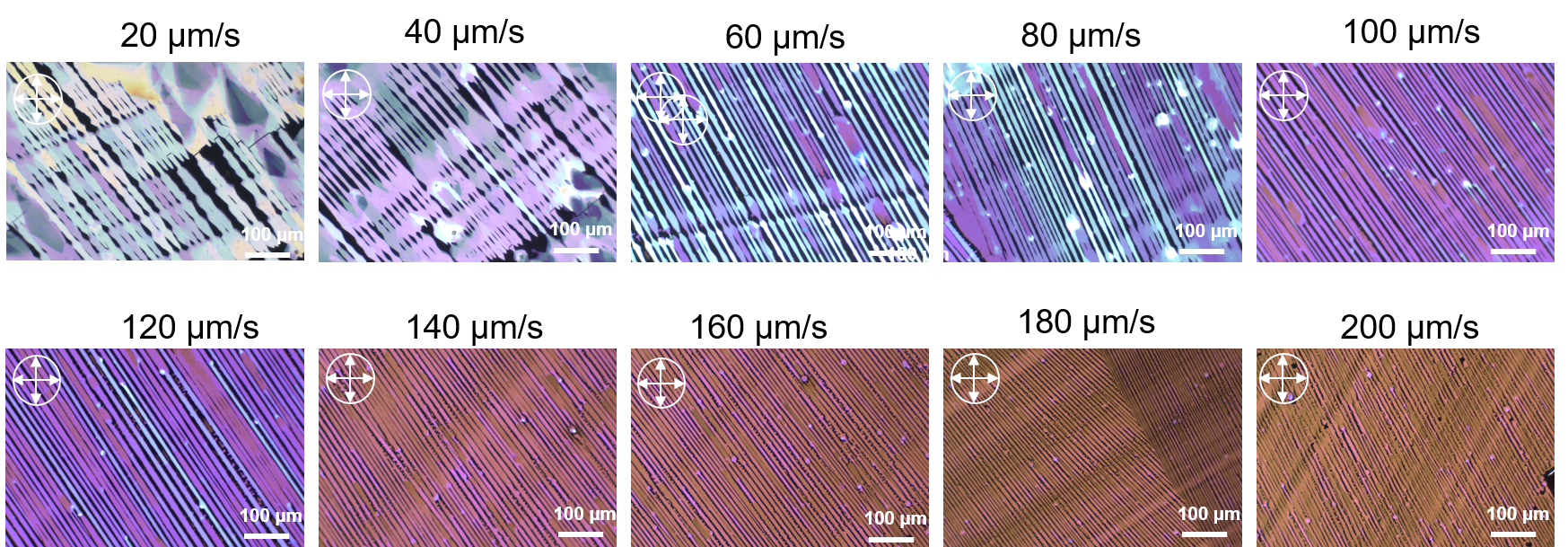


**Figure S47.** POM images of thin film **8** on SiO_2_ from mixed solution (Chloroform: Chlorobenzene V:V = 4:1).


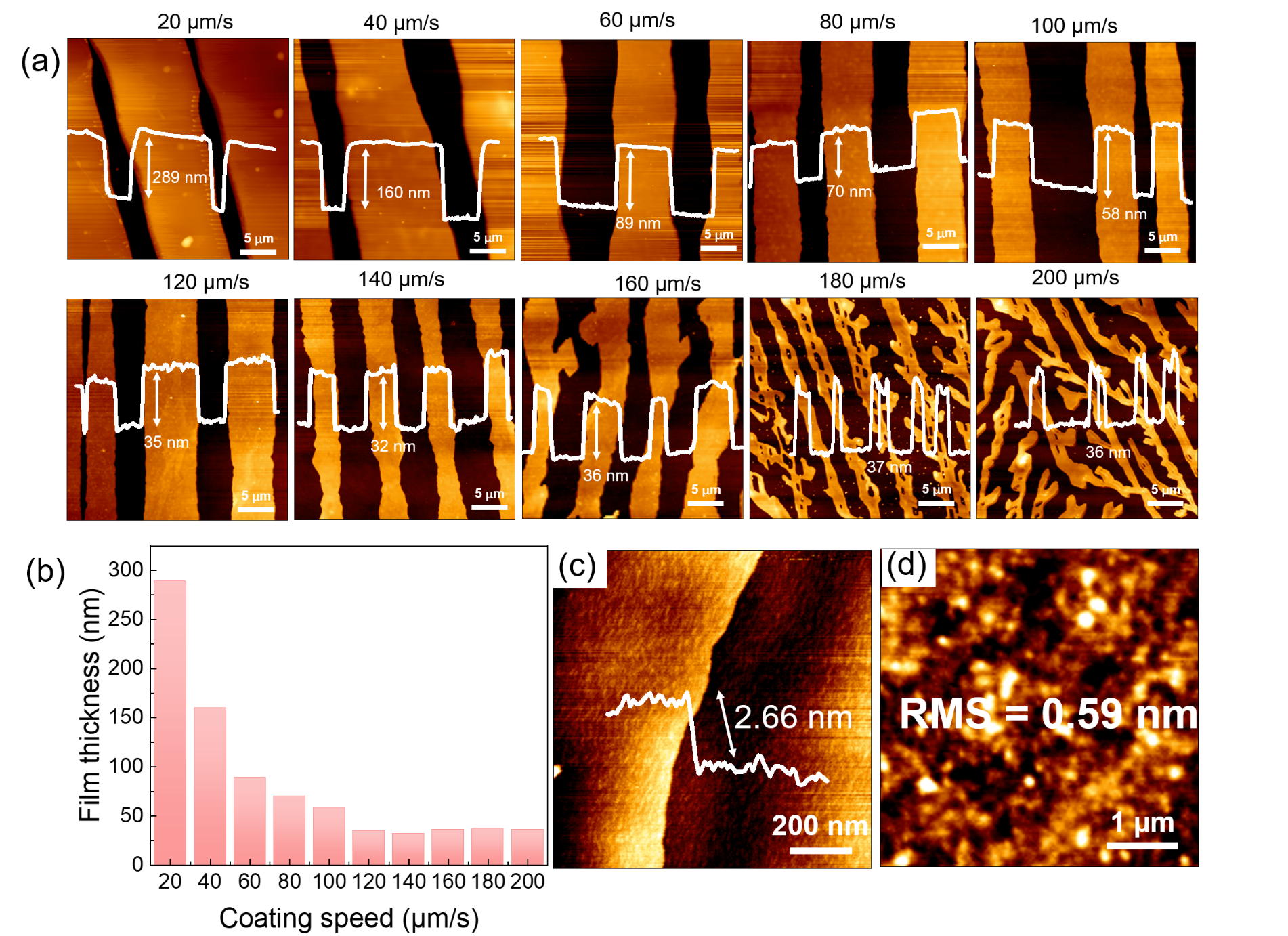


**Figure S48.** AFM measurements of **6**. (a) Tapping-mode (30 × 30 µm) AFM topography. (b) The statistics of film thickness at different coating speeds. (c) AFM height map of monolayer **6**. (d) Localized enlarged surface morphology image.


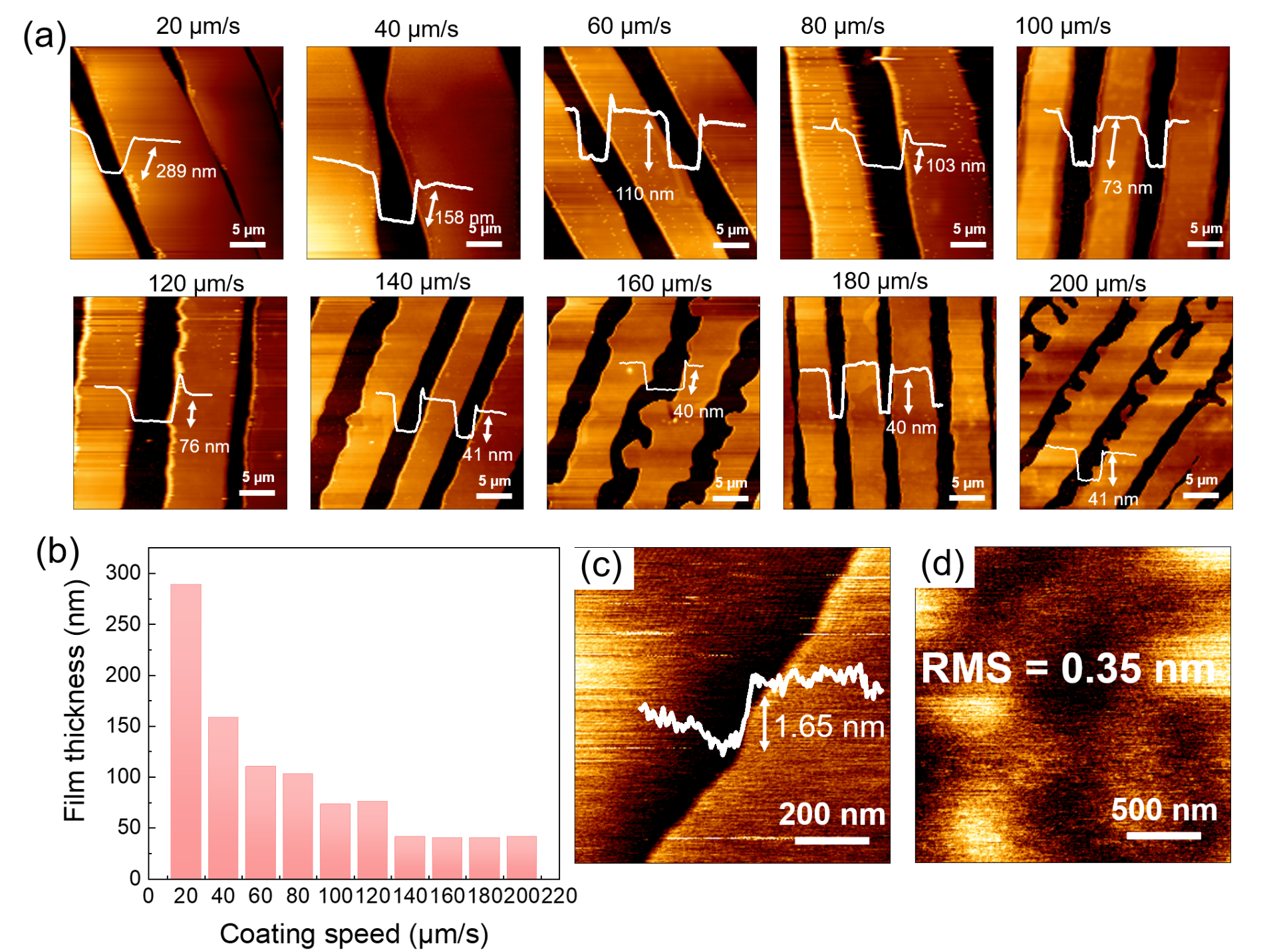


**Figure S49.** AFM measurements of **8**. (a) Tapping-mode (30 × 30 µm) AFM topography. (b) The statistics of film thickness at different coating speeds. (c) AFM height map of monolayer **8**. (d) Localized enlarged surface morphology image.


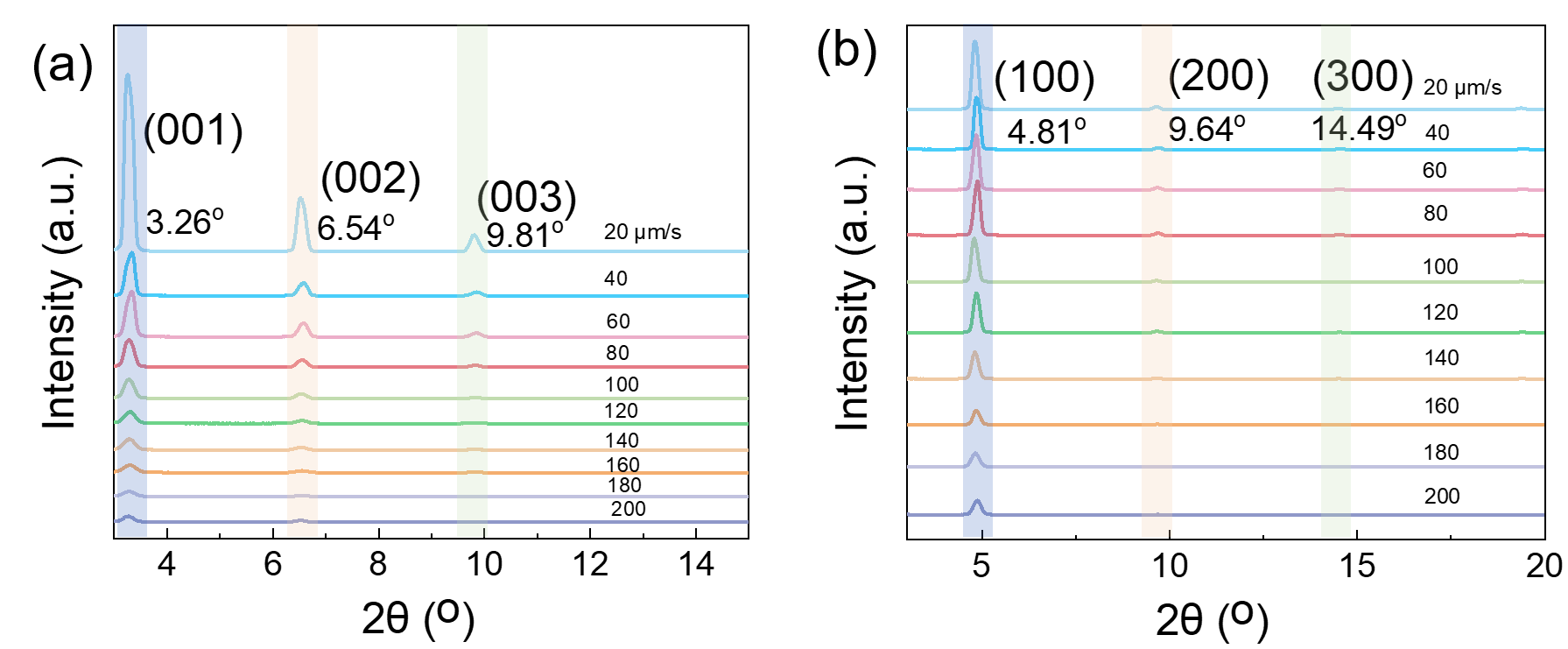


**Figure S50.** XRD diagrams of **6** (a) and **8** (b).


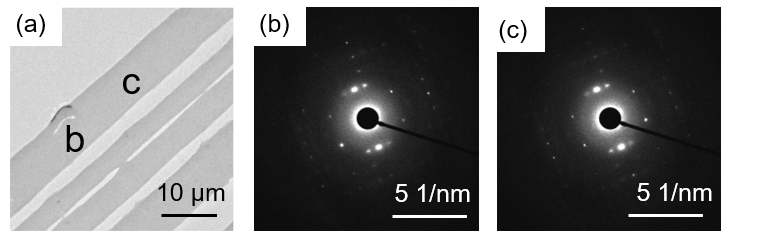


**Figure S51.** TEM image of blade-coated film **8** showing anisotropic ribbon-like domains formed via directional crystallization, and its corresponding SAED patterns recorded from the different positions marked in (a).


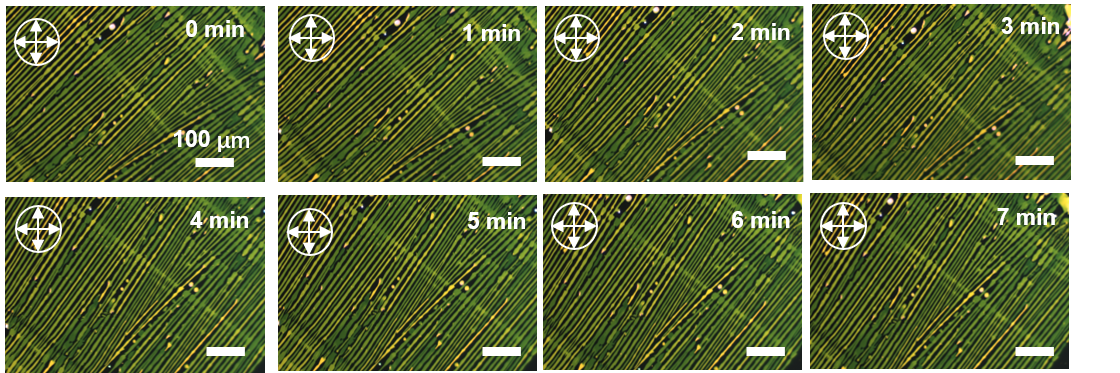


**Figure S52.** POM images of film **6** upon UV illumination for 0-7 min.

**Figure S53.** XRD patterns of film **6** before and after illumination.


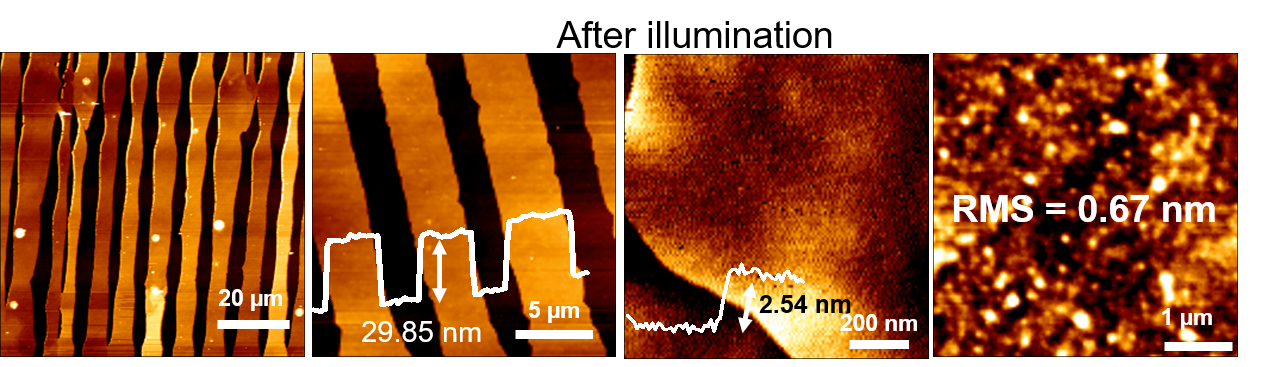


**Figure S54.** AFM images of **6** after illumination.


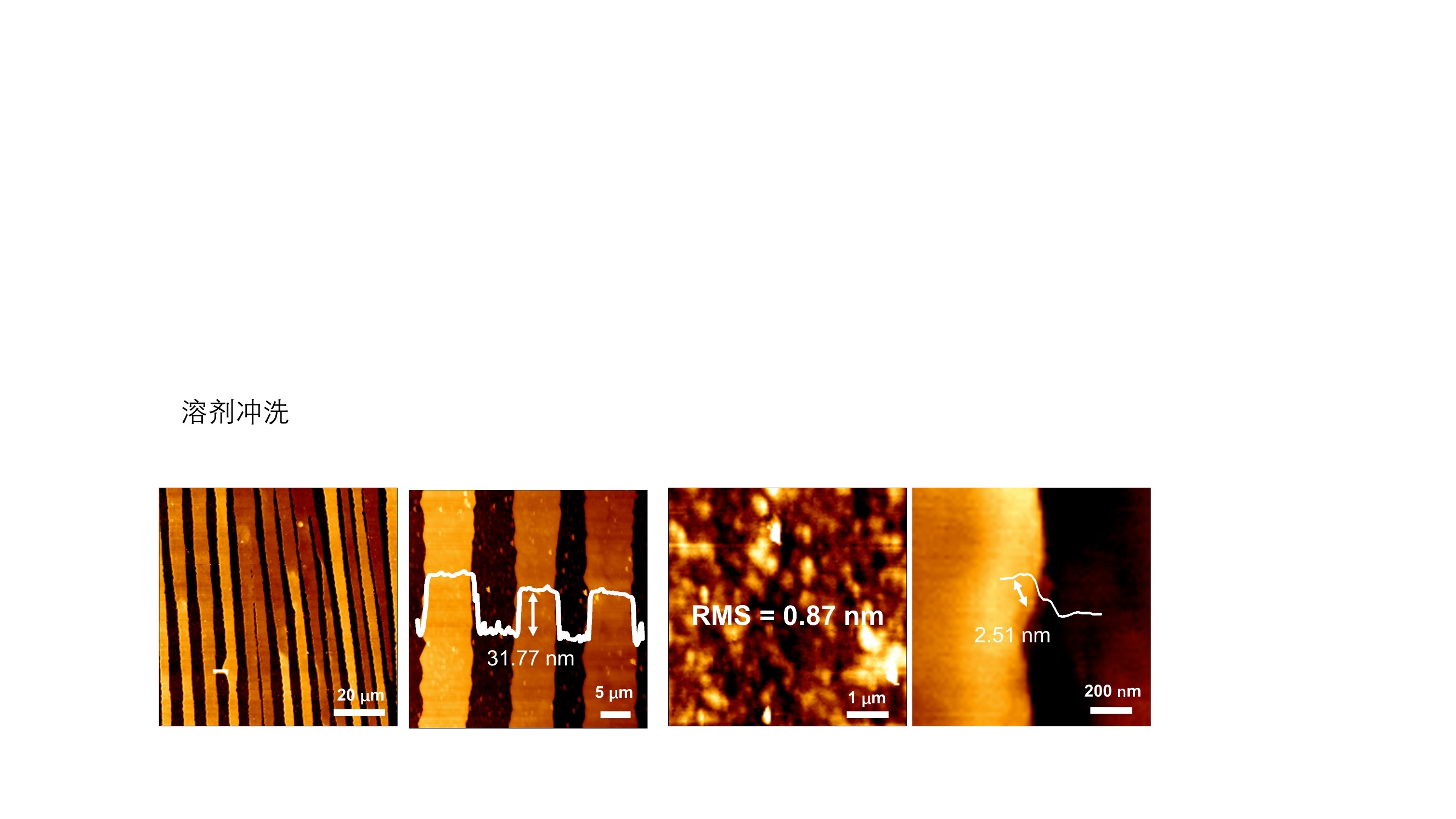


**Figure S55.** AFM images of solvent washed film **6**.

**Figure S56.** UV-vis spectra of film **8** under illumination for 0-20 minutes, and Raman spectra for 0 and 20 minutes of illumination.


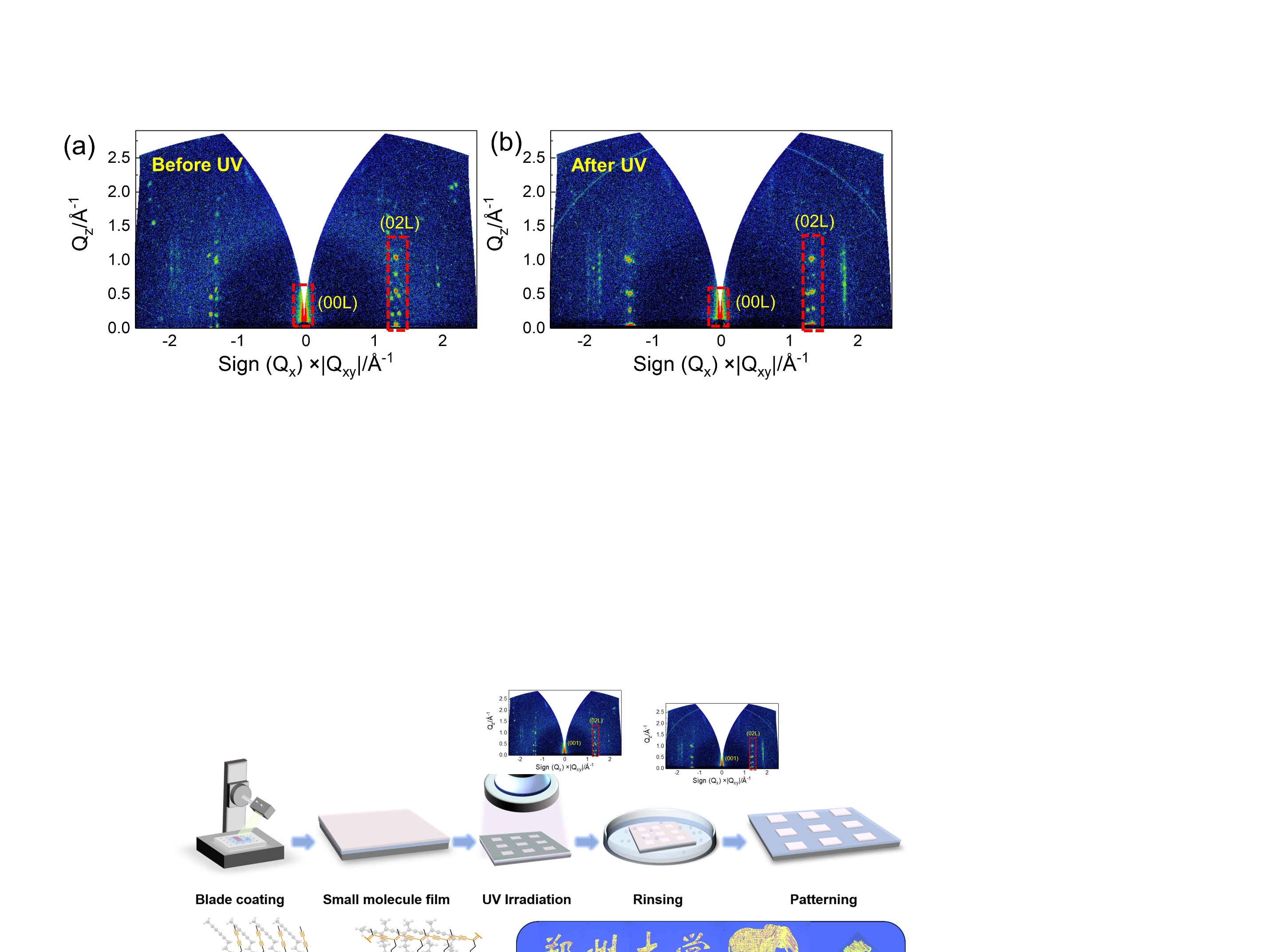


**Figure S57.** GIWAXS images of film **6** with incident X-ray beams perpendicular to the coating direction of the films.


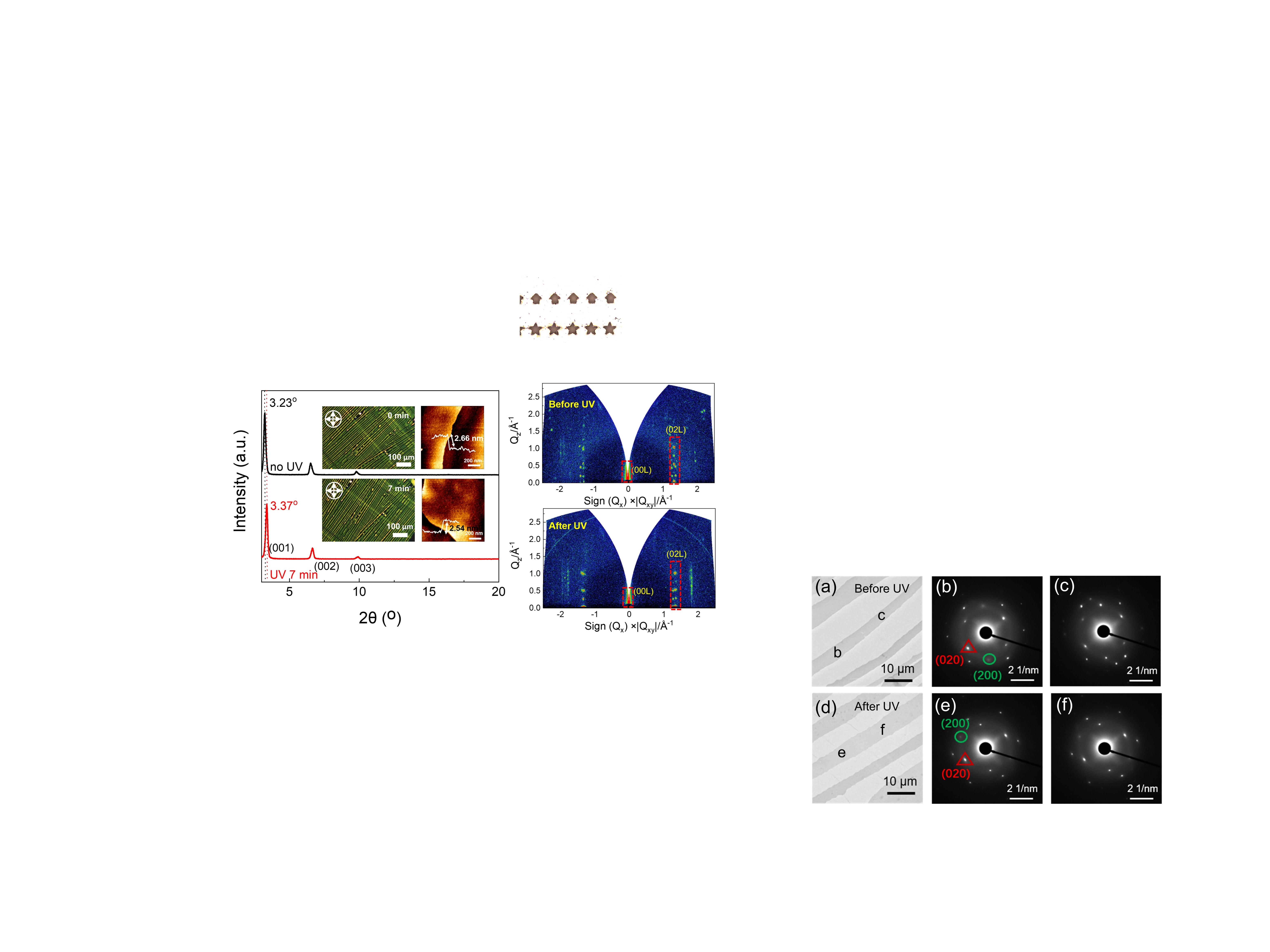


**Figure S58.** (a-c) TEM images of film **6** before illumination and its corresponding SAED patterns recorded from the different positions marked in (a). (d-f) TEM images of film **6** after illumination and its corresponding SAED patterns recorded from the different positions marked in (d).

9. Fabrication of the OTFT devices

**BGTC OTFTs of pristine film**

The unpatterned pristine film was aligned with the skeletonized electrode mask plate and placed in a vaporizer to thermally evaporate Ag (80 nm) as the source-drain electrode.

The charge carrier mobility of TFTs were calculated in the saturation regime by the equation *μ* = (2*L*/*WC_i_*) × (*d|I_d_|*^1/2^/*dV_g_*)^2^. The capacitance of the 300 nm SiO₂ gate dielectric was assumed to be 1.151 ×10-8 F cm⁻². For the patterned film devices, the field-effect mobility was calculated as the average value in the gate voltage range of –60 V to –80 V; for the pristine film devices, mobility was averaged over –70 V to –80 V. These ranges were selected based on the regions where the transfer characteristics showed relatively stable and representative performance (**Figure S67**).

**Patterned BGTC OTFTs of 6**

The electrode mask plate and patterned organic film were pasted into a vaporizer, and the vacuum was set to 8 × 10^-4^ Pa with a vaporization rate of 0.2 Å/s. An 80 nm-thick source and drain were prepared on the surface of the organic semiconductor film using Ag, resulting in an organic thin-film transistor with a bottom-gate top-contact structure.

The electrical performance of the devices was measured under ambient atmosphere (20–30°C, relative humidity ~40%).


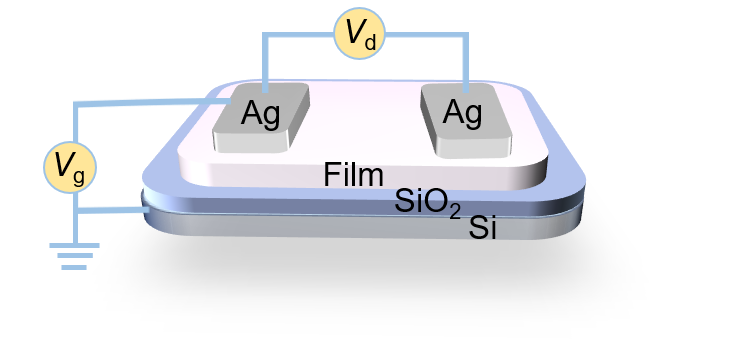


**Figure S59.** Schematic configuration of OTFT.

The appropriate coating speed for each compound was selected by considering both the surface morphology of the films and the resulting device performance, in order to achieve optimal film quality and electrical properties. For compound **6**, all device fabrication and cross-linking experiments, were conducted using films coated at 120 μm/s, this coating speed provided the highest initial mobility. For compound **8**, a coating speed of 140 μm/s was chosen, it resulted in the optimal mobility for this material (**Figure S60**).

**Figure S60.** Mobility statistics corresponding to films prepared at different coating speeds for **6** and **8**.


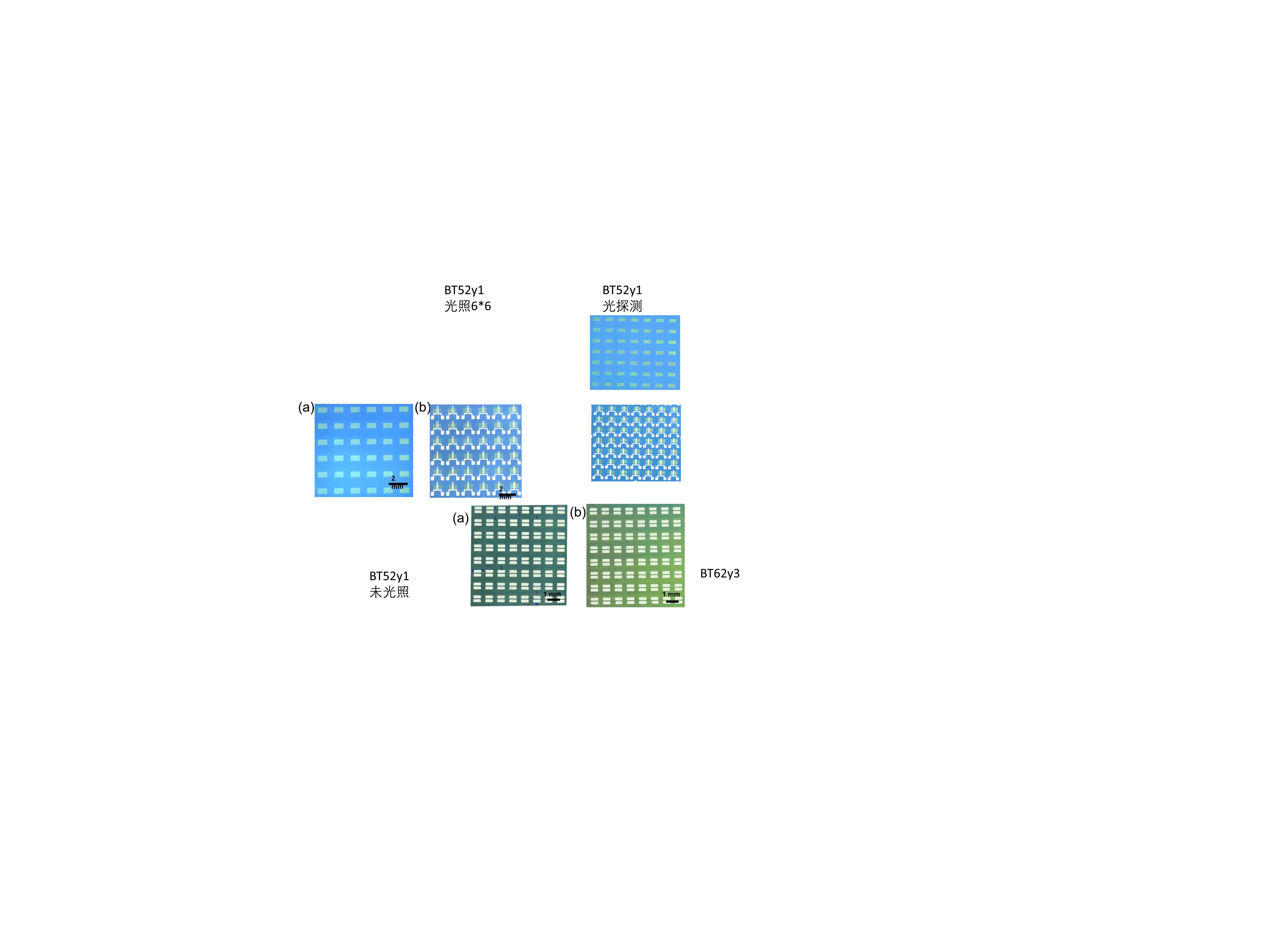


**Figure S61.** POM images of the pristine thin films of **6** (coating speed:120µm/s) (a) and **8** (coating speed:140µm/s) (b) based OTFTs arrays.

**Figure S62.** Typical transfer and output curves of OTFTs based on the pristine thin film of **8**.

To optimize the UV exposure time for patterned device fabrication, we measured the mobility of devices prepared with different exposure times. Each data point represents the average mobility from 5 devices in the same batch. After 0.5 min of UV exposure, devices showed poor mobility following development due to insufficient crosslinking and film removal. At 1 min exposure, the mobility before and after development was nearly unchanged (0.22 cm² V⁻¹ s⁻¹), indicating effective crosslinking and film retention. For longer exposures, mobility declined rapidly, likely due to over-crosslinking or photo-damage. Therefore, 1 min UV exposure was chosen for all device fabrication. The mobility values reported in **Table 1** are based on 36 devices, while **Figure S63** data come from a separate batch (n = 5 per point).

**Figure S63.** Statistics on device mobility for film **6** under different UV exposure times, and whether it is developed or not after illumination.


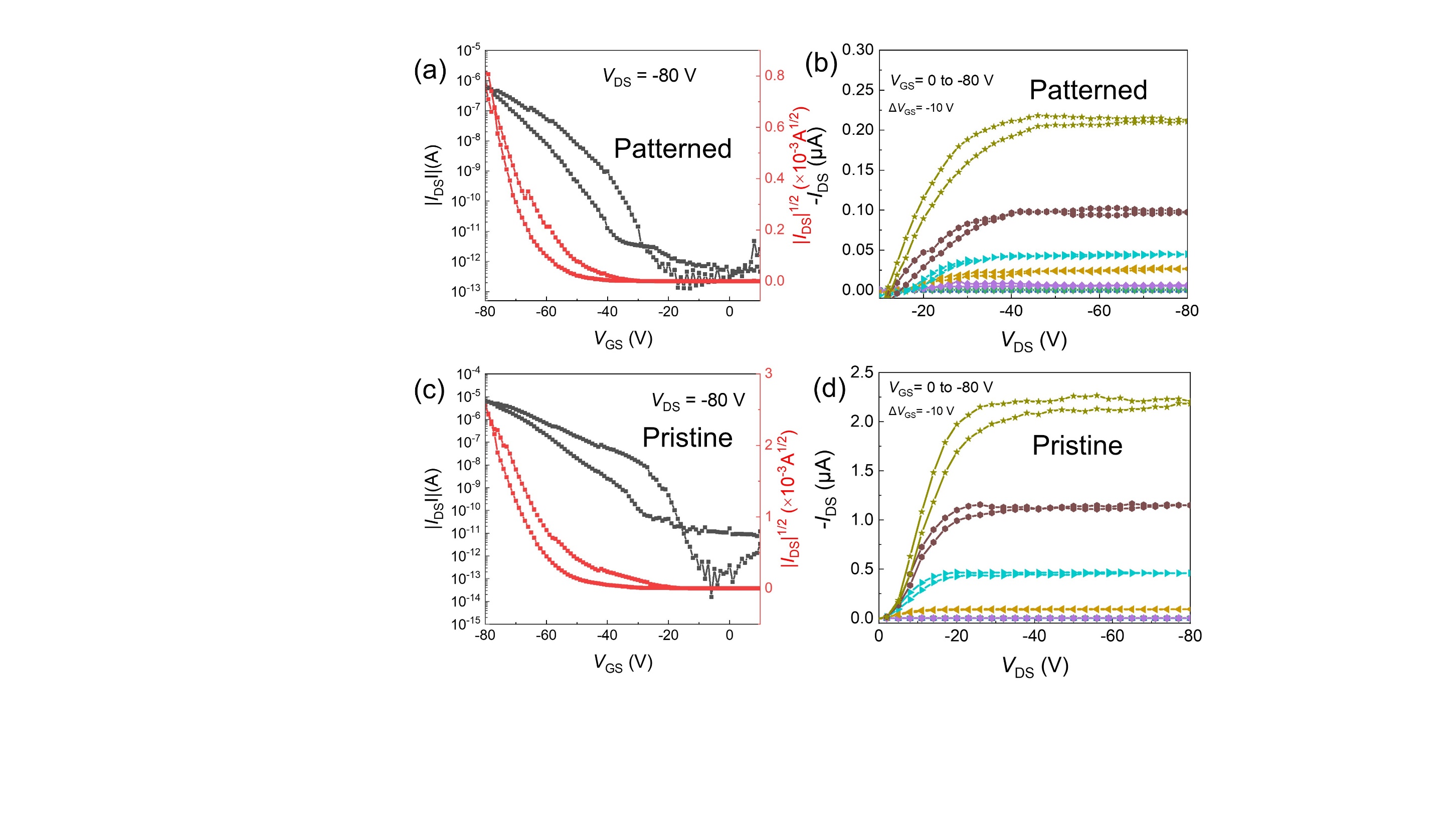


**Figure S64.** Transfer and output characteristics of OTFTs based on (a, b) patterned and (c, d) pristine films **6**, measured in both forward and backward sweep directions. Noticeable hysteresis is observed in both device types.


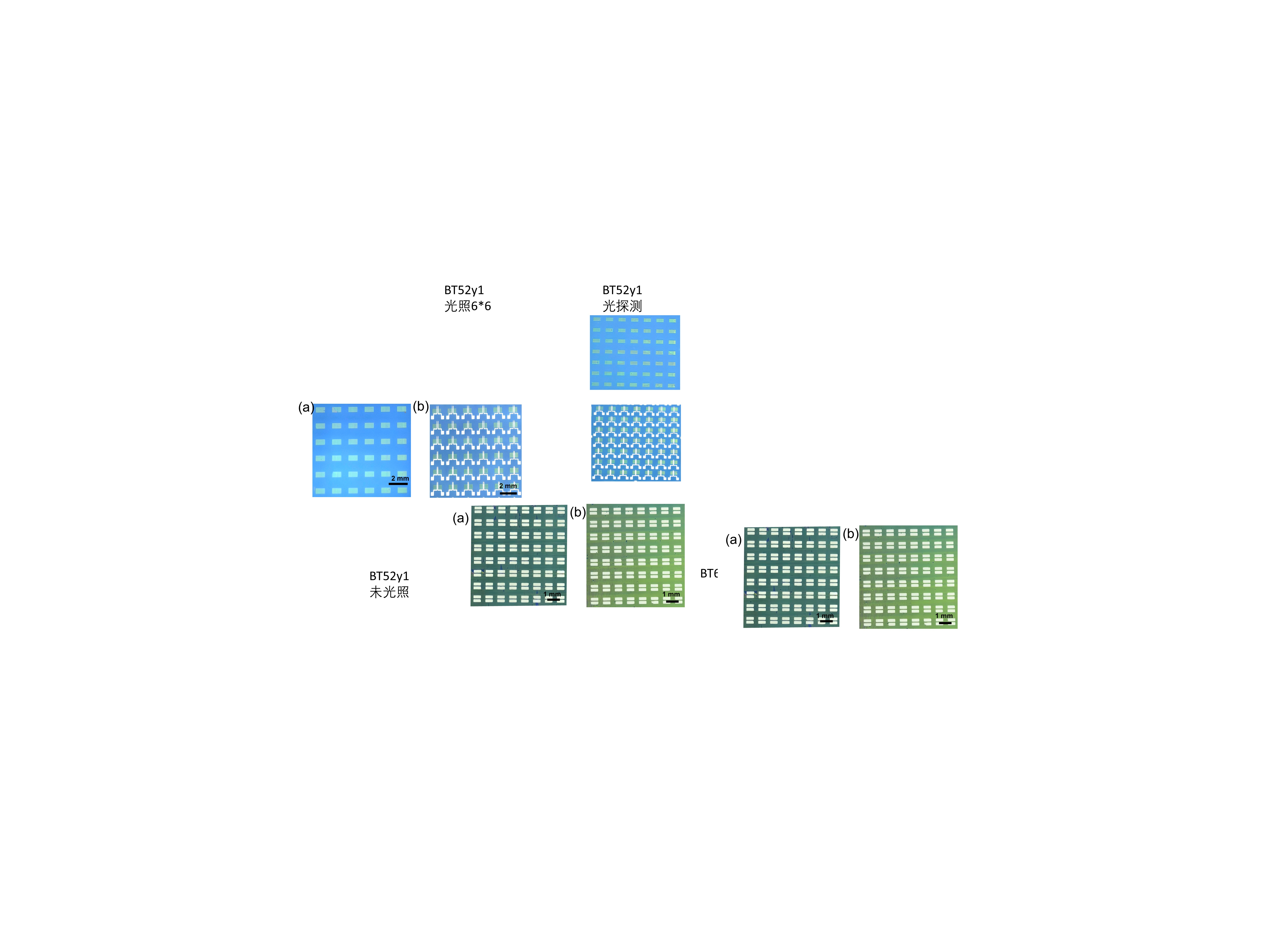


**Figure S65.** POM images of patterned thin film **6** (a) and corresponding OTFTs arrays (b) (light source: 1 mW/cm^2^, illumination time: 1 minute).

**Table S3.** Benchmarking patterning methods and resolution for organic and hybrid electronic materials.

| Patterning Method | Material System | Achieved Resolution | Reference |
| --- | --- | --- | --- |
| Photolithography | Organic light-emitting polymer | 470 nm | 12 |
| photolithography | Chemically amplified resist | 20 nm (LER~3.9 nm) | 13 |
| Photolithography | Self-immolative polymers | 18 nm (LER~1.8 nm) | 14 |
| Photolithography | PMMA photoresist doped with HfOx | 30 nm (LER<10 nm) | 15 |
| Photolithography | PEDOT:PSS | 750 nm | 16 |
| laser writing | Perovskite quantum dots (PQDs) | 1.58 μm | 17 |
| laser writing | PQDs | 2 μm | 18 |
| laser writing | Two-photon polymerization resists | 36 nm | 19 |
| laser writing | Evaporable materials | 50 nm | 20 |
| Photo-Crosslinking | Perovskite–based crosslinking | 5 μm | 21 |
| Photo-Crosslinking | Heavy-metal-free quantum dots with n-LiXer (n=2,4,6) | 1 μm | 22 |
| Photo-Crosslinking | Organic semiconductor/insulator blends with 6Bx | <10 μm | 23 |
| Photo-Crosslinking | Organic semiconductor | LER 65 nm | This work |


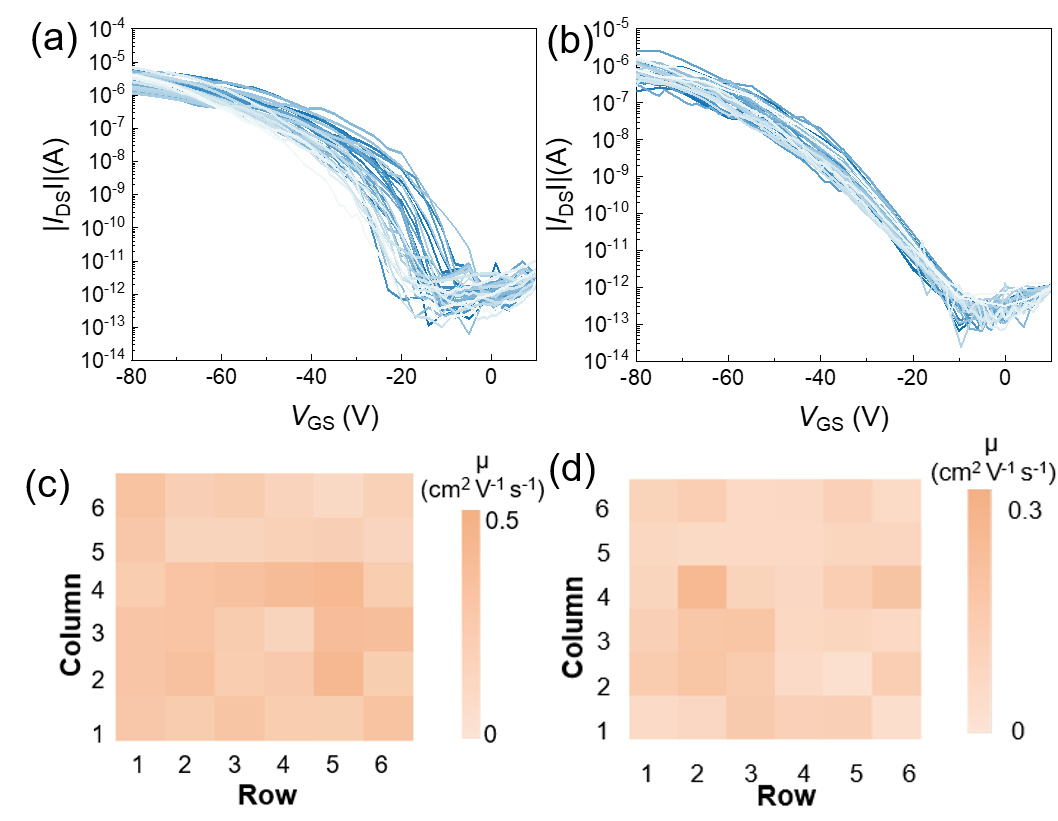


**Figure S66.** The transfer curves and mobility distribution color map of 36 OTFT arrays of pristine (a, c) and patterned (b, d) film **6**.


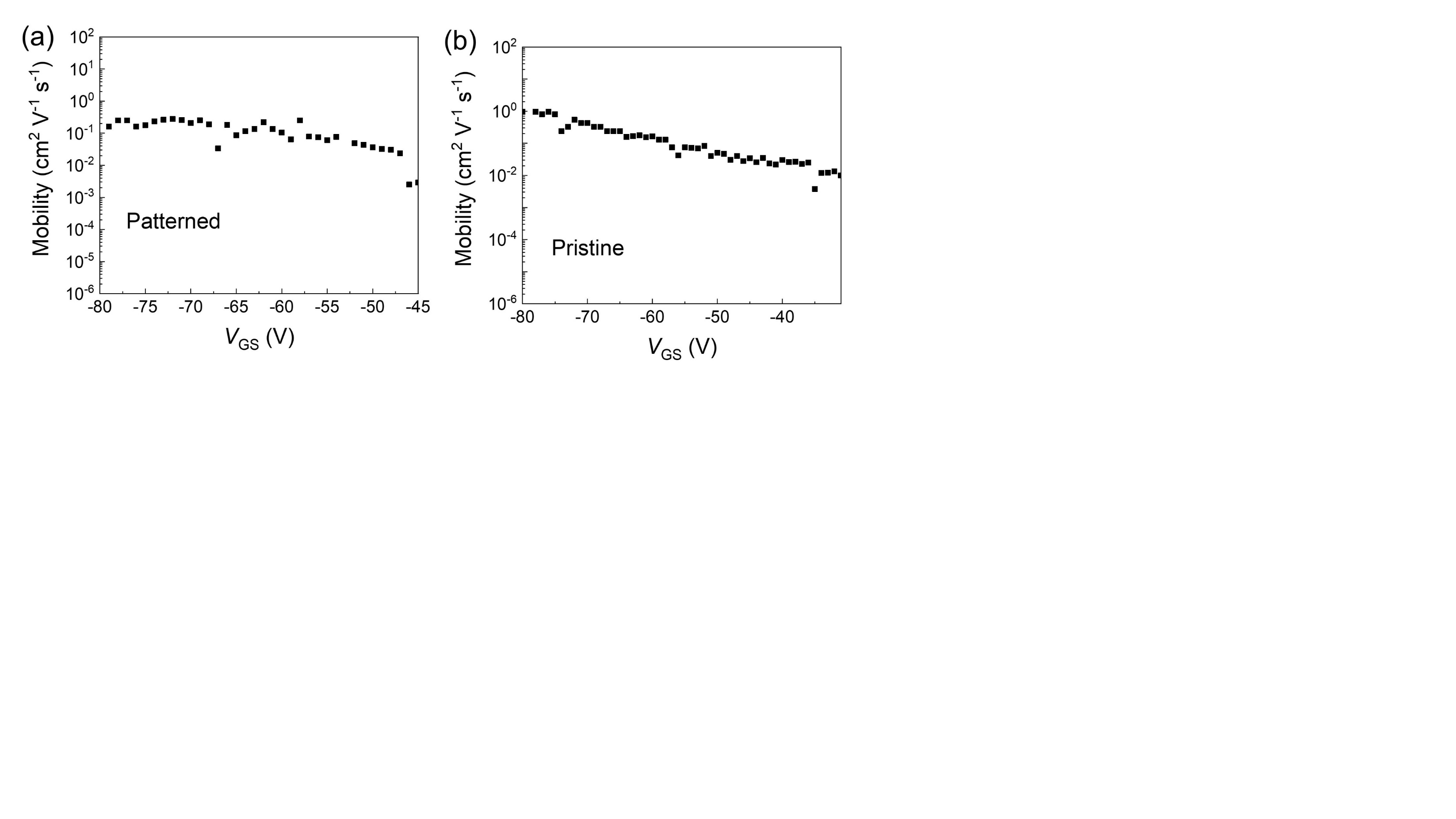


**Figure S67.** Gate voltage dependence of field-effect mobility for (a) patterned and (b) pristine devices, corresponding to **Figures 3a** and **3e** in the main text.

The temperature dependence of both mobility and on/off ratio was investigated for devices based on pristine and patterned film **6** (**Figure S68**). For pristine film device, both mobility and on/off ratio decrease significantly above 60°C, reflecting pronounced thermal sensitivity. Patterned film device exhibit relatively stable mobility and on/off ratio up to 80°C, but a noticeable decrease occurs at higher temperatures. These findings suggest that the crosslinked network in patterned films enhances device thermal stability under moderate conditions, though both device types are susceptible to performance degradation at elevated temperatures.

The breakdown voltage of the gate dielectric was evaluated using a two-terminal Ag/crosslinked organic semiconductor **6**/SiO₂/Si structure. A silver electrode was thermally evaporated onto the crosslinked organic semiconductor film, which was coated on a Si/SiO₂ substrate. During the measurement, a bias voltage was swept from 0 to 200 V between the Ag top electrode and Si substrate (serving as the bottom electrode), while recording the current. The current remained very low up to 124 V, above which an increase indicated the onset of breakdown (**Figure S69a**). No destructive failure was observed, confirming stable device operation. For practical use, the absolute value of the gate voltage was kept at or below 80 V to ensure long-term device reliability. Long-term bias stress stability was evaluated under a gate bias of -10 V (**Figure S69b**) using OTFT devices based on patterned films. The results show that the devices maintain stable mobility and exhibit only minor shifts in threshold voltage over 1 hour, demonstrating reasonable bias stress stability.
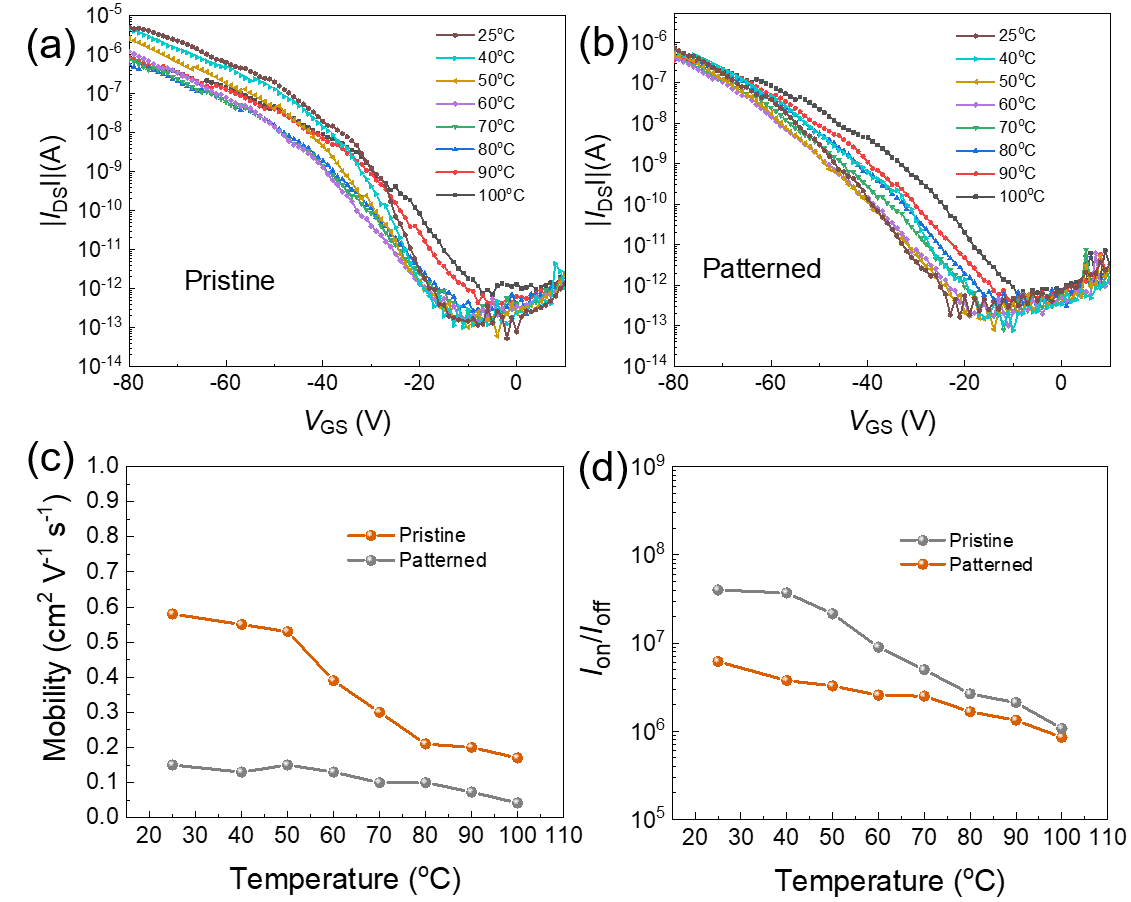


**Figure S68.** Transfer curves of OTFT devices based on (a) pristine film **6** and (b) patterned film **6** measured at different temperatures. Mobility (c) and on/off ratio (d) of both device types measured at temperatures from 25°C to 100°C.


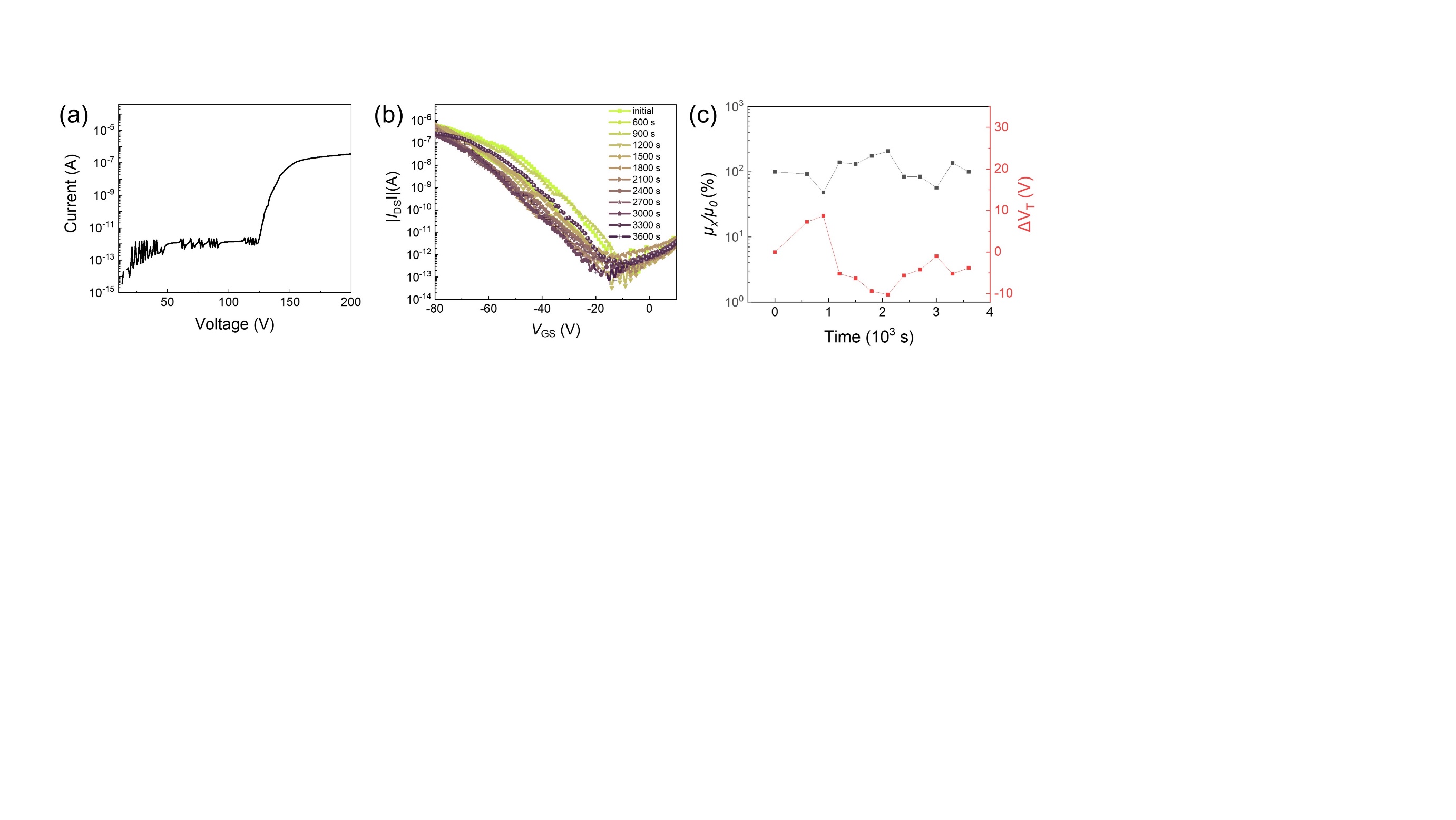


**Figure S69.** (a) I–V curve of the Ag/crosslinked organic semiconductor **6**/SiO₂/Si device. (b) The transfer characteristics under different negative gate voltage stress times. (c) The plot of mobility and threshold voltage with gate bias voltage times.


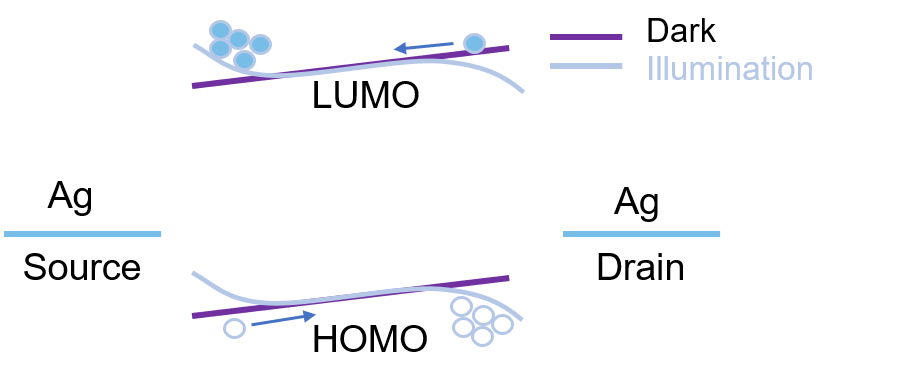


**Figure S70.** Energy band diagram of electrode/semiconductor interface in dark and under illumination.


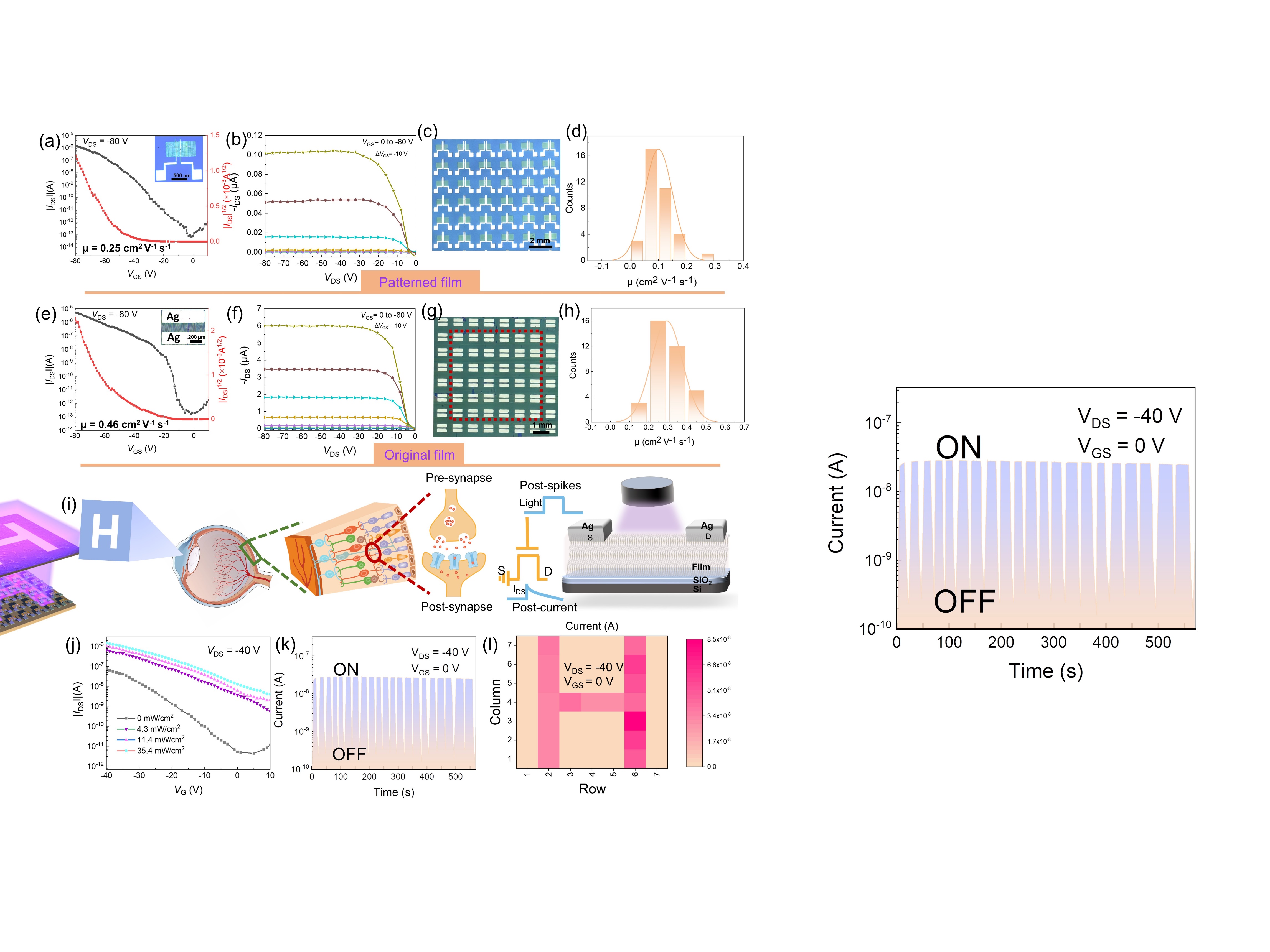


**Figure S71.** Photo-response measurements of the device in dark and under illumination.

The OTFT devices in this work used a 300 nm t SiO₂ dielectric layer, resulting in a relatively low gate capacitance and therefore requiring high gate voltages (|V_GS_| up to 80 V) to induce sufficient charge carriers in the channel. The observed low on-current (~10⁻⁶–10⁻⁷ A) is attributed to the combination of low field-effect mobility, thick gate dielectric and large channel length (>100 μm) in the device structure.

For future optimization, the operational gate voltage can be significantly reduced and on-current improved by using high-k or ultrathin gate dielectrics, molecular engineering of the semiconductor to enhance charge transport and optimizing device geometry and electrode interfaces. It should be noted that for bio-inspired imaging arrays and neuromorphic vision sensors, as demonstrated in this work, low operating currents and high stability are often more critical than high current drive, making the present device performance suitable for proof-of-concept demonstrations.


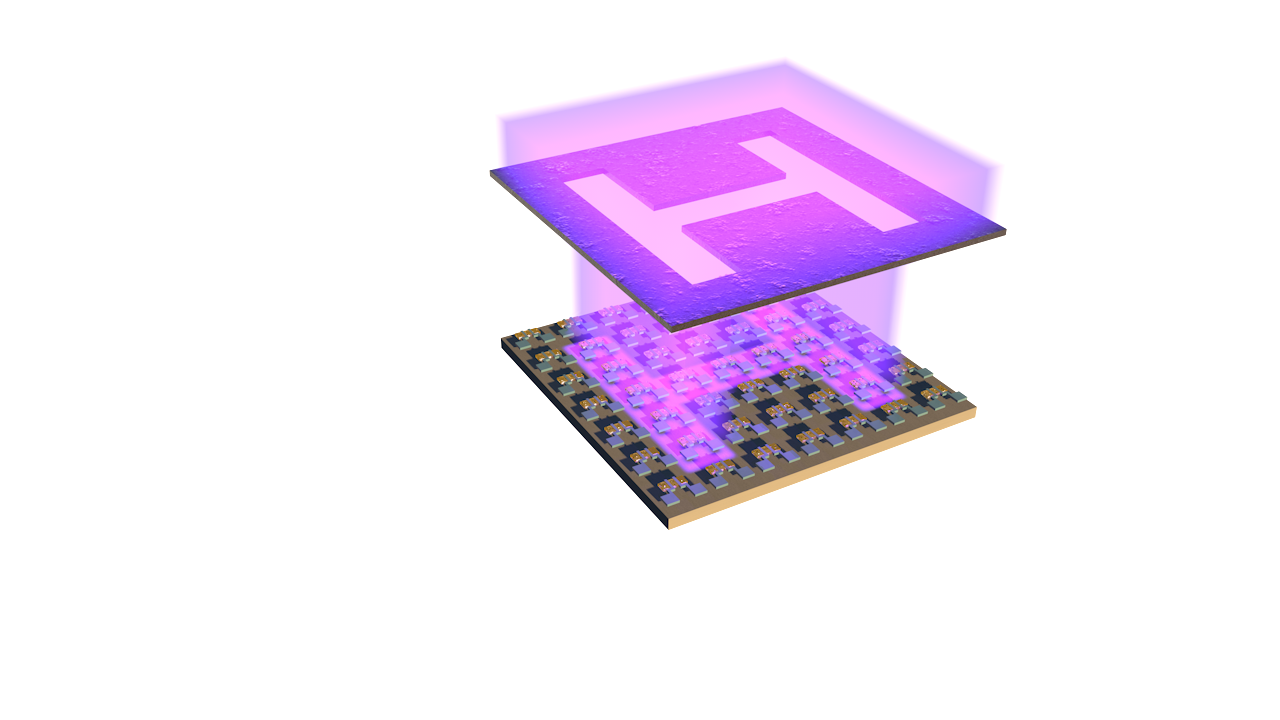


**Figure S72.** The illustration of an OTFT array used for pattern recognition by using UV irradiation through a photomask.

Recent advances in photo-crosslinkable polymer semiconductors, especially those based on DPP derivatives^[24,25]^, have demonstrated impressive device mobilities, robust solvent resistance, and uniform patterned films. These polymeric systems benefit from mechanical flexibility and excellent processability, making them suitable for a variety of flexible and large-area device applications.

In contrast, intrinsic photo-crosslinkable small-molecule OSCs, as demonstrated in this work, offer unique advantages that complement those of polymer-based systems. Small molecules have well-defined structures and low dispersity, enabling highly reproducible film formation and consistent device performance. Their rigid π-conjugated backbones and tailored side chains facilitate long-range crystalline packing and well-defined molecular arrangements, which contribute to reliable charge transport and tunable defect engineering.

10. Supporting references

[1] S. Otep, K. Ogita, N. Yomogita, K. Motai, Y. Wang, Y.-C. Tseng, C.-C. Chueh, Y. Hayamizu, H. Matsumoto, K. Ishikawa, T. Mori, T. Michinobu, *Macromolecules* **2021**, *54*, 4351–4362.

[2] X. Hu, J. Ding, H. Chen, Y. Hou, H. Li, Y. Sun, X. Liu, *Sci. Bull.* **2024**, *69*, 2675–2678.

[3] G. W. T. M. J. Frisch, H. B. Schlegel, G. E. Scuseria, ; M. A. Robb, J. R. C., G. Scalmani, V. Barone, ; G. A. Petersson, H. N., X. Li, M. Caricato, A. V. Marenich, ; J. Bloino, B. G. J., R. Gomperts, B. Mennucci, H. P. Hratchian, ; J. V. Ortiz, A. F. I., J. L. Sonnenberg, D. Williams-Young, ; F. Ding, F. L., F. Egidi, J. Goings, B. Peng, A. Petrone, ; T. Henderson, D. R., V. G. Zakrzewski, J. Gao, N. Rega, ; G. Zheng, W. L., M. Hada, M. Ehara, K. Toyota, R. Fukuda, ; J. Hasegawa, M. I., T. Nakajima, Y. Honda, O. Kitao, H. Nakai, ; T. Vreven, K. T., J. A. Montgomery, Jr., J. E. Peralta, ; F. Ogliaro, M. J. B., J. J. Heyd, E. N. Brothers, K. N. Kudin, ; V. N. Staroverov, T. A. K., R. Kobayashi, J. Normand, ; K. Raghavachari, A. P. R., J. C. Burant, S. S. Iyengar, ; J. Tomasi, M. C., J. M. Millam, M. Klene, C. Adamo, R. Cammi, ; J. W. Ochterski, R. L. M., K. Morokuma, O. Farkas, ; J. B. Foresman, and D. J. Fox, *Gaussian 16 Revision A. 03. 2016; Gaussian Inc. Wallingford CT 2016.*

[4] G. A. Petersson, A. Bennett, T. G. Tensfeldt, M. A. Al‐Laham, W. A. Shirley, J. Mantzaris, *J. Chem. Phys.* **1988**, *89*, 2193–2218.

[5] G. A. Petersson, M. A. Al‐Laham, *J. Chem. Phys.* **1991**, *94*, 6081–6090.

[6] P. J. Stephens, F. J. Devlin, C. F. Chabalowski, M. J. Frisch, *J. Chem. Phys.* **1994**, *98*, 11623–11627.

[7] A. D. Becke, *J. Chem. Phys.* **1993**, *98*, 5648–5652.

[8] H. Ebata, T. Izawa, E. Miyazaki, K. Takimiya, M. Ikeda, H. Kuwabara, T. Yui, *J. Am. Chem. Soc.* **2007**, *129*, 15732–15733.

[9] S. Lee, M. Jang, H. Yang, *ACS Appl. Mater. Interfaces* **2014**, *6*, 20444–20451.

[10] S. B. Lee, S. Lee, D. G. Kim, S. H. Kim, B. Kang, K. Cho, *Adv. Funct. Mater.* **2021**, *31*, 2100196.

[11] Z. Zhang, B. Peng, X. Ji, K. Pei, P. K. L. Chan, *Adv. Funct. Mater.* **2017**, *27*, 1703443.

[12] J. Li, Y. Guo, K. Wang, W. Huang, H. Su, W. Li, X. Zhou, Y. Zhang, T. Guo, C. Wu, *Nano-Micro Lett.* **2025**, *17*, 228.

[13] V. S. V. Satyanarayana, F. Kessler, V. Singh, F. R. Scheffer, D. E. Weibel, S. Ghosh, K. E. Gonsalves, *ACS Appl. Mater. Interfaces* **2014**, *6*, 4223–4232.

[14] J. Cen, W. Liu, J. Xu, X. Wang, J. Zhang, J. Zhang, Z. Deng, C. Zhou, J. Hu, S. Liu, *Angew. Chem. Int. Ed.* **2025**, *64*, e202415588.

[15] M. Wang, M. Yu, N. Guan, Y. Wu, W. Zhu, Y. Wu, S. Zhu, R. Tai, J. Zhao, Y. Xu, *ACS Appl. Polym. Mater.* **2025**, *7*, 6542–6551.

[16] Y. Ni, C. Xue, X. Zhao, P. Xue, Y. Tong, Q. Tang, Y. Liu, *IEEE Electron Device Lett.* **2025**, *46*, 604–607.

[17] S.-Y. Liang, Y.-F. Liu, S.-Y. Wang, H. Xia, H.-B. Sun, *Nanoscale* **2022**, *14*, 1174–1178.

[18] S.-Y. Liang, Y.-F. Liu, Z.-K. Ji, S.-Y. Wang, H. Xia, H.-B. Sun, *Nano Lett.* **2023**, *23*, 3769–3774.

[19] M. He, Z. Zhang, C. Cao, Y. Qiu, X. Shen, G. Zhou, Z. Cai, X. Sun, X. He, L. Xu, X. Liu, C. Ding, Y. Cao, C. Kuang, X. Liu, *PhotoniX* **2022**, *3*, 25.

[20] S. Zeng, T. Tian, J. Oh, Z.-H. Lin, C.-J. Shih, *Nat. Commun.* **2025**, *16*, 3436.

[21] D. Liu, K. Weng, S. Lu, F. Li, H. Abudukeremu, L. Zhang, Y. Yang, J. Hou, H. Qiu, Z. Fu, X. Luo, L. Duan, Y. Zhang, H. Zhang, J. Li, *Sci. Adv.* **2022**, *8*, eabm8433.

[22] J. Yang, M. Lee, S. Y. Park, M. Park, J. Kim, N. Sitapure, D. Hahm, S. Rhee, D. Lee, H. Jo, Y. H. Jo, J. Lim, J. Kim, T. J. Shin, D. C. Lee, K. Kwak, J. S. Kwon, B. Kim, W. K. Bae, M. S. Kang, *Adv. Mater.* **2022**, *34*, 2205504.

[23] M. Lee, B. Choi, P. Ahn, Y. Y. Choi, Y. Heo, J. Kim, J. H. Min, T. J. Shin, K. Kim, H. Choi, H. Kweon, D. H. Ho, J. I. Yoon, H. Kim, E. Lee, D. H. Kim, K. Kwak, M. S. Kang, J. H. Cho, B. Kim, *Chem. Mater.* **2022**, *34*, 10409–10423.

[24] X. Xue, C. Li, Q. Zhou, X. Yu, C. Gao, K. Chenchai, J. Liao, Z. Shangguan, X. Zhang, G. Zhang, D. Zhang, *Adv. Mater.* **2024**, *36*, 2407305.

[25] C. Gao, D. Shi, C. Li, X. Yu, X. Zhang, Z. Liu, G. Zhang, D. Zhang, *Adv. Sci.* **2022**, *9*, 2106087.

**Coordinates for calculated geometries:**

**6**

S -0.613625 1.585794 -1.216030

C -0.035665 0.665805 0.150582

C -0.794387 0.115952 -2.147958

C -0.415950 -1.041746 -1.443798

C -0.559366 -2.294145 -2.066167

H -0.316356 -3.070576 -1.615776

C -1.057645 -2.363075 -3.335551

H -1.141180 -3.194771 -3.743747

C -1.448734 -1.207335 -4.045940

C -1.304717 0.032293 -3.435216

H -1.549893 0.805298 -3.889643

C -2.037606 -1.390123 -5.419593

H -1.339834 -1.739103 -5.995650

H -2.724297 -2.072100 -5.357627

C -2.641019 -0.196575 -6.104794

H -1.938745 0.448619 -6.284545

H -3.277342 0.220735 -5.503821

C -3.342451 -0.529947 -7.406678

H -2.733257 -1.035010 -7.967541

H -4.102449 -1.101461 -7.211707

C -3.823398 0.657190 -8.172316

H -3.057814 1.200525 -8.416551

H -4.390624 1.191291 -7.594797

C -4.595239 0.313036 -9.427616

H -4.053456 -0.275296 -9.977294

H -5.397316 -0.172544 -9.179427

C -4.976410 1.472344 -10.218442

C -5.263624 2.465208 -10.833227

C -5.564557 3.598612 -11.559054

C -5.823365 4.576672 -12.179256

C -6.137739 5.771432 -12.921332

H -6.128567 5.576213 -13.860519

H -5.484697 6.448249 -12.727986

H -7.008512 6.085081 -12.667076

S 0.613625 -1.585794 1.216030

C 0.035665 -0.665805 -0.150582

C 0.794387 -0.115952 2.147958

C 0.415950 1.041746 1.443798

C 0.559366 2.294145 2.066167

H 0.316356 3.070576 1.615776

C 1.057645 2.363075 3.335551

H 1.141180 3.194771 3.743747

C 1.448734 1.207335 4.045940

C 1.304717 -0.032293 3.435216

H 1.549893 -0.805298 3.889643

C 2.037606 1.390123 5.419593

H 1.339834 1.739103 5.995650

H 2.724297 2.072100 5.357627

C 2.641019 0.196575 6.104794

H 1.938745 -0.448619 6.284545

H 3.277342 -0.220735 5.503821

C 3.342451 0.529947 7.406678

H 2.733257 1.035010 7.967541

H 4.102449 1.101461 7.211707

C 3.823398 -0.657190 8.172316

H 3.057814 -1.200525 8.416551

H 4.390624 -1.191291 7.594797

C 4.595239 -0.313036 9.427616

H 4.053456 0.275296 9.977294

H 5.397316 0.172544 9.179427

C 4.976410 -1.472344 10.218442

C 5.263624 -2.465208 10.833227

C 5.564557 -3.598612 11.559054

C 5.823365 -4.576672 12.179256

C 6.137739 -5.771432 12.921332

H 6.128567 -5.576213 13.860519

H 5.484697 -6.448249 12.727986

H 7.008512 -6.085081 12.667076

**7**

S 2.883306 -0.011297 16.088598

C 1.705509 -0.088727 14.810161

C 1.705072 -0.694578 13.513752

C 2.667755 -1.464868 12.842183

H 3.514791 -1.627745 13.240451

C 2.370662 -1.984298 11.592202

H 3.024193 -2.510934 11.147295

C 1.127082 -1.757752 10.956578

C 0.178104 -0.976475 11.605981

H -0.656502 -0.792285 11.190878

C 0.462527 -0.464038 12.874833

C 0.851732 -2.442501 9.629915

H 0.961320 -3.417955 9.755901

H 1.539350 -2.148630 8.981051

C -0.521976 -2.190543 9.017056

H -0.589281 -1.237911 8.756995

H -1.216703 -2.367655 9.699768

C -0.796228 -3.061382 7.791338

H -0.051026 -2.960423 7.147237

H -0.831988 -4.011220 8.068292

C -2.106317 -2.690047 7.104492

H -2.015954 -1.789870 6.701611

H -2.825952 -2.646777 7.783010

C -2.508392 -3.691531 6.009973

H -1.770150 -3.768620 5.354549

H -2.644775 -4.581940 6.419773

C -3.733791 -3.291300 5.308749

C -4.723748 -2.927152 4.735130

C -5.856892 -2.518428 4.060865

C -6.839976 -2.157677 3.476462

C -8.046909 -1.748149 2.748280

H -7.831294 -0.963060 2.185295

H -8.737435 -1.471200 3.401188

C -8.619153 -2.852452 1.860444

H -7.929825 -3.141621 1.211160

H -8.858186 -3.632992 2.420494

C -9.854188 -2.372948 1.107100

H -9.617650 -1.603752 0.547709

H -10.546520 -2.109504 1.748745

H -10.192770 -3.097157 0.540251

S -0.643941 0.463177 13.865412

C 0.533856 0.540607 15.143849

C 0.534292 1.146458 16.440259

C -0.428390 1.916748 17.111827

H -1.275427 2.079625 16.713559

C -0.131297 2.436178 18.361808

H -0.784829 2.962814 18.806715

C 1.112283 2.209632 18.997432

C 2.061260 1.428355 18.348029

H 2.895867 1.244165 18.763132

C 1.776838 0.915918 17.079178

C 1.387633 2.894381 20.324096

H 1.278044 3.869835 20.198109

H 0.700015 2.600510 20.972959

C 2.761341 2.642423 20.936955

H 2.828646 1.689791 21.197015

H 3.456067 2.819535 20.254243

C 3.035593 3.513262 22.162673

H 2.290391 3.412303 22.806774

H 3.071353 4.463100 21.885718

C 4.345682 3.141927 22.849518

H 4.255318 2.241750 23.252400

H 5.065317 3.098657 22.171000

C 4.747756 4.143411 23.944038

H 4.009515 4.220500 24.599461

H 4.884140 5.033820 23.534237

C 5.973156 3.743180 24.645261

C 6.963112 3.379032 25.218880

C 8.096257 2.970308 25.893145

C 9.079341 2.609557 26.477548

C 10.286274 2.200029 27.205730

H 10.070659 1.414940 27.768715

H 10.976800 1.923080 26.552822

C 10.858518 3.304332 28.093567

H 10.169190 3.593501 28.742850

H 11.097551 4.084872 27.533517

C 12.093553 2.824828 28.846910

H 11.857015 2.055632 29.406301

H 12.785884 2.561384 28.205265

H 12.432134 3.549037 29.413760

**8**

C 8.950635 4.283564 -0.226384

C 9.298129 4.710543 2.206139

C 10.388522 4.047794 1.614654

C 11.490772 3.763412 2.431414

H 12.236517 3.342104 2.070752

C 11.475012 4.101268 3.757538

H 12.213939 3.899526 4.284659

C 10.374735 4.742952 4.342365

C 9.285140 5.041108 3.545580

H 8.543167 5.464846 3.912900

C 10.370325 5.146435 5.799438

H 10.896843 5.955021 5.892655

H 9.457717 5.363570 6.051346

C 10.891513 4.132866 6.768230

H 11.780133 3.860636 6.487469

H 10.320910 3.349396 6.740487

C 10.963697 4.637625 8.198669

H 11.520324 5.431628 8.221974

H 10.072188 4.895271 8.482760

C 11.509995 3.633778 9.173010

H 12.403088 3.379373 8.890030

H 10.956258 2.838155 9.146377

C 11.575845 4.132056 10.599011

H 10.693982 4.431832 10.869784

H 12.173093 4.895271 10.640070

C 12.056595 3.076356 11.568913

H 11.483159 2.298557 11.492341

H 12.952990 2.804937 11.313675

C 12.078098 3.509007 12.963841

C 12.085532 3.844432 14.101312

C 12.107609 4.211456 15.416339

C 12.142994 4.546071 16.562688

C 12.407685 5.050830 17.962055

H 13.333510 5.332782 18.041955

H 11.839652 5.812425 18.152928

C 12.126352 3.949759 18.925298

H 12.690757 3.191405 18.707792

H 11.204865 3.668617 18.817655

C 12.359303 4.334607 20.400127

H 12.874091 5.144004 20.442296

H 12.834279 3.628917 20.844018

H 11.511932 4.470722 20.832921

S 8.028629 5.047752 1.033157

C 10.150365 3.818506 0.226384

C 9.802871 3.391527 -2.206139

C 8.712478 4.054276 -1.614654

C 7.610228 4.338658 -2.431414

H 6.864483 4.759966 -2.070752

C 7.625988 4.000802 -3.757538

H 6.887061 4.202544 -4.284659

C 8.726265 3.359118 -4.342365

C 9.815860 3.060962 -3.545580

H 10.557833 2.637224 -3.912900

C 8.730675 2.955635 -5.799438

H 8.204157 2.147049 -5.892655

H 9.643283 2.738500 -6.051346

C 8.209487 3.969204 -6.768230

H 7.320867 4.241434 -6.487469

H 8.780090 4.752674 -6.740487

C 8.137303 3.464445 -8.198669

H 7.580676 2.670442 -8.221974

H 9.028812 3.206799 -8.482760

C 7.591005 4.468292 -9.173010

H 6.697912 4.722697 -8.890030

H 8.144742 5.263915 -9.146377

C 7.525155 3.970014 -10.599011

H 8.407018 3.670238 -10.869784

H 6.927907 3.206799 -10.640070

C 7.044405 5.025714 -11.568913

H 7.617841 5.803513 -11.492341

H 6.148010 5.297133 -11.313675

C 7.022902 4.593063 -12.963841

C 7.015468 4.257638 -14.101312

C 6.993391 3.890614 -15.416339

C 6.958006 3.555999 -16.562688

C 6.693315 3.051240 -17.962055

H 5.767490 2.769288 -18.041955

H 7.261348 2.289645 -18.152928

C 6.974648 4.152311 -18.925298

H 6.410243 4.910665 -18.707792

H 7.896135 4.433453 -18.817655

C 6.741697 3.767463 -20.400127

H 6.226909 2.958066 -20.442296

H 6.266721 4.473153 -20.844018

H 7.589068 3.631348 -20.832921

S 11.072371 3.054318 -1.033157
